# From perfluoroalkyl aryl sulfoxides to *ortho* thioethers

**DOI:** 10.3762/bjoc.20.181

**Published:** 2024-08-23

**Authors:** Yang Li, Guillaume Dagousset, Emmanuel Magnier, Bruce Pégot

**Affiliations:** 1 Université Paris-Saclay, UVSQ, CNRS, UMR 8180, Institut Lavoisier de Versailles, 78035 Versailles Cedex, France

**Keywords:** *ortho* functionalization, rearrangement, sulfoxide

## Abstract

Access to original *ortho* thioether derivatives was achieved through a [3,3]-rearrangement in a one-pot two-step protocol. Several aryl-SCF_3_ compounds are reported by variation of the nitrile or of the trifluoroalkyl sulfoxide starting material. The variation of the perfluoroalkyl chain was also possible.

## Introduction

**Scheme 1:**
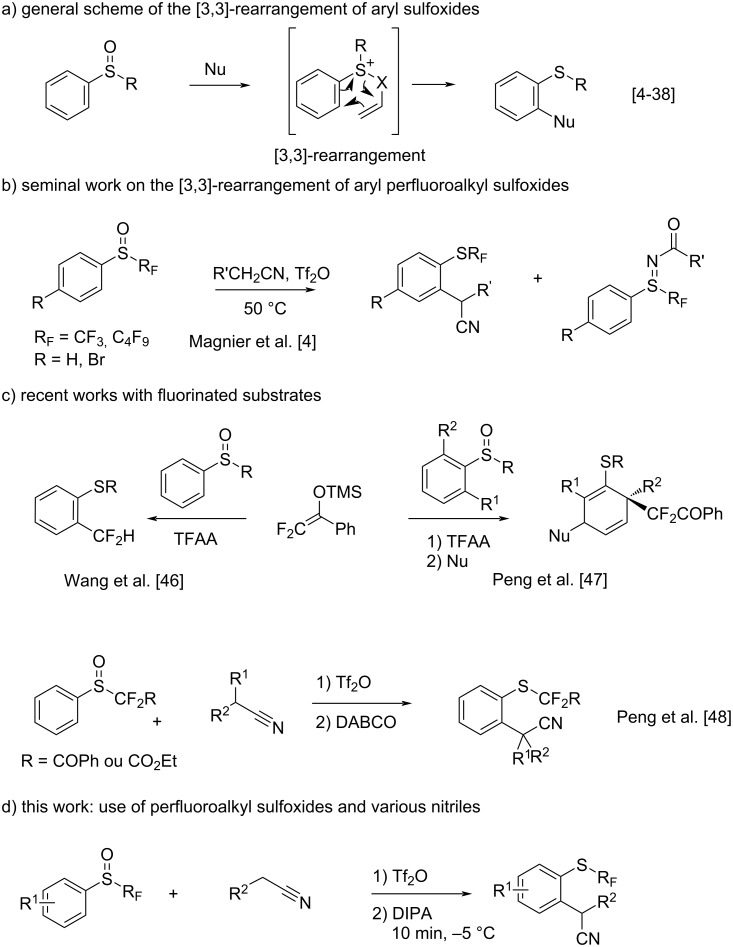
[3,3]-Rearrangement of aryl sulfoxides.

Since decades, sigmatropic rearrangements have established themselves as robust and versatile tools for many transformations in organic synthesis [1–3]. They were widely employed with a wide range of substrates. With a peculiar type of scaffold, *S*-perfluoroalkyl aryl sulfoxides, in 2009, we were the first to demonstrate their ability to be engaged in such a rearrangement [4–5]. Upon activation with trifluoromethanesulfonic anhydride and under heating, we showed their transformation to *ortho* thioethers with a fairly acceptable selectivity towards the pathway of sulfilimine synthesis (Scheme 1b). Following our seminal study, many research groups described a strategy for *ortho*-C–H functionalization of aryl sulfoxides with various nucleophiles via a cascade reaction of interrupted Pummerer reaction/sigmatropic rearrangement (Scheme 1a) [6–11]. A large range of nucleophiles, such as phenols [12–16], anilines [17], carbonyls [18–21], propargyl/allylsilanes [22–34], ynamides [35–37], and alkyl nitriles [38–40], were found to be suitable for this process. Whereas the addition of fluorine atoms to molecules is a well-established strategy to improve or modulate their physicochemical and biological properties [41–45], only few publications have reported a [3,3]-rearrangement with fluorinated molecules (Scheme 1c). In 2020, Wang and co-workers have developed a one-pot [3,3]-sigmatropic rearrangement/Haller–Bauer reaction of aryl sulfoxides with difluoroenoxysilanes as nucleophile under mild reaction conditions [46]. This provided access to organosulfur compounds *ortho*-functionalized by CF_2_H. At the same time Peng and co-workers described the dearomatization of aryl sulfoxides using the same difluoroenol silyl ether with trifluoromethanesulfonic anhydride, allowing the incorporation of two difluoroalkyl groups [47]. By blocking the rearomatization after the [3,3]-rearrangement, external nucleophiles could be trapped to give mono-difluoroalkylated cycles. More recently, in 2019, Peng’s group reported also the *ortho*-cyanoalkylation of benzoyl or ester group-substituted fluoroalkyl aryl sulfoxides with various alkyl nitriles in two steps [48]. The addition of a base in the second step easily enabled the [3,3]-rearrangement, allowing for the addition of two functional groups – the cyano group and difluoromethylthio group – to arenes in good yield.

Inspired and stimulated by this abundant literature, and as part of our research program focused on creating novel perfluoroalkylsulfur derivatives, we became interested in a reappraisal of our previous study with the aim of increasing its scope as well as the yield and selectivity (Scheme 1d). It is important to mention that during the preparation of this paper, a similar study appeared. Peng and co-workers demonstrated the efficient use of acetonitrile as nucleophile with various aryl difluoroalkyl sulfoxides but only one example of an SCF_3_ compound was reported [49].

## Results and Discussion

We started our optimization with the reaction between acetonitrile and phenyl trifluoromethyl sulfoxide (**1a**, Table 1). We firstly chose the same stoichiometry as described in our previous study and tried to reduce the reaction time by the help of microwave heating (Table 1, entry 1). Under these conditions, a significant amount of degradation products was observed and the yield was rather low. The same result was obtained when the reagent was first added at 0 °C and then heated for one hour under microwave irradiation (Table 1, entry 2). To avoid degradation, the temperature was reduced while the reaction time was increased with twice the number of equivalents of acetonitrile (−15 °C to rt, for 12 hours, entry 3 in Table 1) without any significant improvement in the yield. As previously reported, the use of an organic base can improve the yield of this reaction [26,38,40,48]. Therefore, we decided to use 2 equivalents of DIPEA at low temperature. After ten minutes at −15 °C to allow for the reaction between phenyl trifluoromethyl sulfoxide (**1a**) and acetonitrile, the base was added and the reaction was stirred for the same amount of time. To our delight, a good NMR yield of 74% was received under these conditions (Table 1, entry 4). The importance of the temperature was then evaluated (Table 1, entries 5–7). A too low value was deleterious to the yield, whereas −5 °C appeared as the conditions of choice. Finally, by adjusting to 5 equivalents of nitrile and base, resulted in the optimal conditions (Table 1, entry 9). Other organic nitrogenous bases were tested (Table 1, entries 10–12). Et_3_N gave nearly the same result, while DBU seemed less efficient. The use of the inorganic base K_2_CO_3_ resulted in poor outcomes.

**Table 1:** Optimization of the reaction conditions.

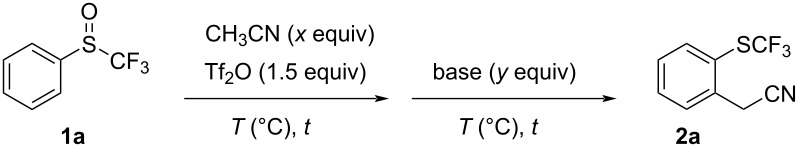						

Entry	*T* (°C)	*t*	*x*	base	*y*	NMR yield (%)^a,b^

1	50 °C (MW)	1 h	1.5	–		21
2	0 to 50 °C (MW)	1 h	1.5	–		21
3	−15 to 20 °C	12 h	3	–		38
4	−15 °C	10 min	3	DIPEA	2	74
5	−30 °C	10 min	3	DIPEA	2	41
6	−5 °C	10 min	3	DIPEA	2	77
7	0 °C	10 min	3	DIPEA	2	75
8	−5 °C	10 min	5	DIPEA	2.5	80
9	−5 °C	10 min	5	DIPEA	5	95 (79)
10	−5 °C	10 min	5	Et_3_N	5	85
11	−5 °C	10 min	5	DBU	5	48
12	−5 °C	10 min	5	K_2_CO_3_	5	2

^a^Experimental conditions: **1a** (0.5 mmol), Tf_2_O (1.5 equiv), *T* (°C), *t* (min or h), then addition of base (*y* equiv) at the same temperature and time as the first step (*T*, *t*). ^b19^F NMR spectroscopic yields, isolated yields in parentheses.

**Scheme 2:**
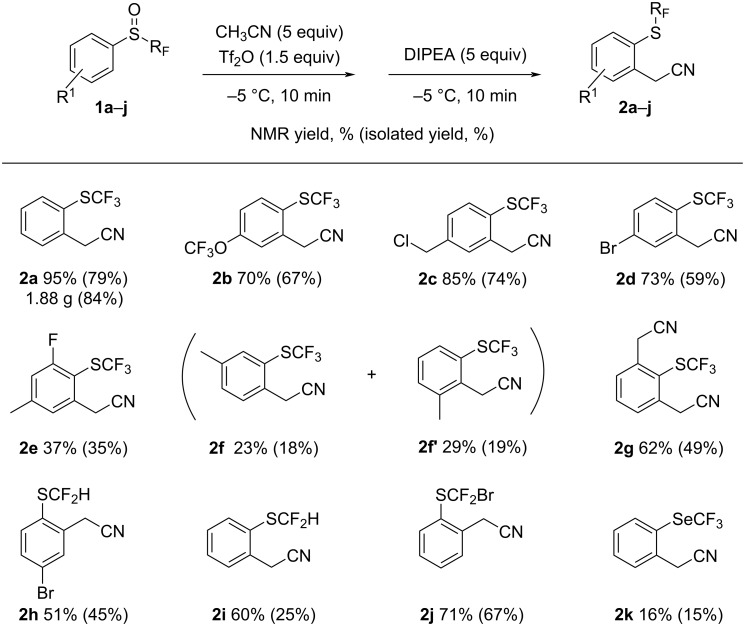
The scope of aryl perfluoromethyl sulfoxides and a selenoxide.

With the optimized conditions in hand, a scale-up was successfully performed, resulting in the production of 1.88 g of product **2a** corresponding to 84% yield (Scheme 2). The reaction with other aryl sulfoxides was then investigated. We observed that the rearrangement product was isolated in good yield (**2b**–**d**) when the sulfoxide is *para*-substituted whereas the *meta* or difunctionalization led to lower yields (**2e**,**f**). The product of rearrangement **2a** was oxidized into the sulfoxide and re-engaged under the optimized conditions to afford the compound of bis-rearrangement **2g** in a good yield of 49%. This compound is then the result of an iterative rearrangement. Difluorinated sulfoxides **1h**–**j** proved also efficient for this rearrangement giving rise to the corresponding thioethers **2h**–**j** in good NMR yields and lower isolated yield in the case of the more volatile adduct **2i**. Finally, trifluoromethyl selenoxide **1k** was tested as a substrate, and the rearranged product was obtained in a low yield of 15%. The main product obtained was phenyl(trifluoromethyl)selane, a reduction product of the selenoxide. Despite a low yield, this result is encouraging because it is the first example of rearrangement with aryl trifluoromethyl selenoxide.

**Scheme 3:**
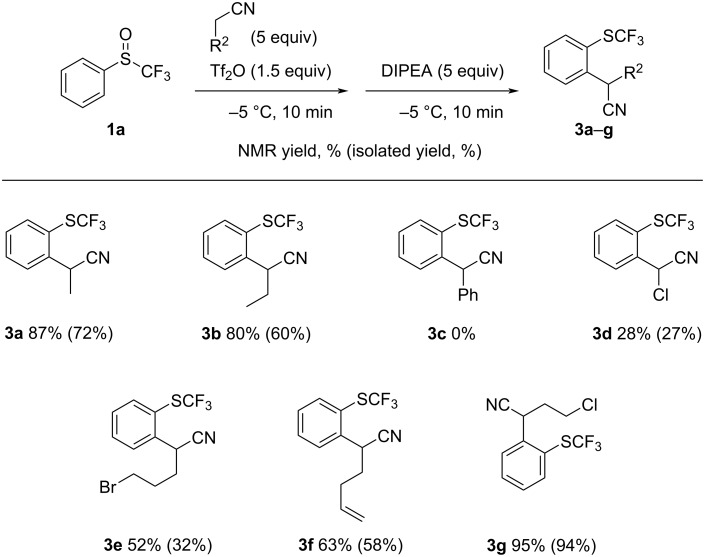
The scope of alkyl nitriles.

We further investigated the generality of the reaction using a series of nitriles with the sulfoxide **1a** (Scheme 3). We noticed that the length of the alkyl chain has no impact on the yield (**3a**,**b**). However, the use of benzyl cyanide is completely deleterious for the reaction as no product was observed (**3c**). The presence of a chlorine atom at the alpha-position of the nitrile is also detrimental to the reaction, resulting in less than 30% yield of the desired product **3d**. Nevertheless, the reaction is compatible with halogens elsewhere in longer nitrile alkyl chains (**3e**,**g**). Finally, it was possible to obtain the terminal alkene **3f** with a yield of 58% using hex-5-enenitrile.

## Conclusion

In summary, fine-tuning of the experimental conditions gave us access to original *ortho*-cyanoalkylated aryl perfluoroalkylsulfur derivatives. We have also shown that structural diversity is possible by varying the substituents on the aromatic ring, the perfluoroalkyl chain, and the alkyl chain linking the cyano functional groups. The [3,3]-sigmatropic rearrangement of perfluoroalkyl selenoxides needs to be optimized to improve the yield and decrease the amount of reduction product. The complete evaluation of the potential of these new compounds will be provided in the future.

## Experimental

### General procedure for the rearrangement process

Sulfoxide (0.5 mmol, 1 equiv), nitrile (5 equiv) and Tf_2_O (1.5 equiv) were added in the described order to a 5 mL flask under an argon atmosphere, maintained at −5 °C. The reaction mixture was stirred for 10 min, then DIPEA (5 equiv) was slowly added to the flask with a syringe and the reaction was stirred for another 10 min. At the end of the reaction, 1 mL of chloroform and a known amount of trifluoromethoxybenzene were added to the flask in order to determine the ^19^F NMR yield. To purify the product, the reaction mixture was mixed with a sufficient volume of a saturated NH_4_Cl solution, then extracted 3 times with diethyl ether. The combined organic layers were dried over MgSO_4_, filtered, concentrated under reduced pressure, and purified by preparative TLC or flash chromatography.

## Supporting Information

File 1 Experimental procedures, characterization data of all isolated products as well as copies of NMR spectra for novel compounds.

## Data Availability

The data that supports the findings of this study is available from the corresponding author upon reasonable request.
