# Supplementary material for: From perfluoroalkyl aryl sulfoxides to ortho thioethers
Source: Beilstein J Org Chem. 2024 Aug 23;20:2108–13. doi: 10.3762/bjoc.20.181 (PMC11346307; doi:10.3762/bjoc.20.181)
Supplement: File 1 — Experimental procedures, characterization data of all isolated products as well as copies of NMR spectra for novel compounds. [file Beilstein_J_Org_Chem-20-2108-s001.pdf]

BEILSTEIN JOURNAL OF ORGANIC CHEMISTRY

Supporting Information

for

From perfluoroalkyl aryl sulfoxides to *ortho* thioethers

Yang Li, Guillaume Dagousset, Emmanuel Magnier and Bruce Pégot

Beilstein J. Org. Chem. doi:

Experimental procedures, characterization data of all isolated products as well as copies of NMR spectra for novel compounds

License and Terms: This is a supporting information file under the terms of the Creative Commons Attribution License (https://creativecommons.org/licenses/by/4.0). Please note that the reuse, redistribution and reproduction in particular requires that the author(s) and source are credited and that individual graphics may be subject to special legal provisions.
The license is subject to the *Beilstein Journal of Organic Chemistry* terms and conditions: (https://www.beilstein-journals.org/bjoc/terms)

Table of contents

General information	S2
General procedure 1: Selective oxidation of trifluoromethyl aryl sulfides	S2
General procedure 2: Rearrangement	S25

General Information

Unless otherwise noted, all commercial reagents and solvents were purchased from commercial sources (Sigma-Aldrich, Alfa Aesar, Fluorochem, Apollo, ABCR, Carlo Erba) and used without further purification. Diisopropylethylamine (DIPEA) was distilled from KOH and stored on KOH. Dichloromethane (DCM) and acetonitrile (MeCN) were purified by distillation. Chloroform was directly drawn from Sure/Seal™ bottles. Thin-layer chromatography (TLC) analysis of reaction mixtures was performed using Merck silica gel 60 F254 TLC plates, and visualized under UV 254 nm then staining with $KMnO_4$. Chromatographic purification of the products was accomplished using flash chromatography on silica gel (SiO_2, 0.04–0.0063 mm) purchased from Sigma-Aldrich, and/or automated flash chromatography machine Puriflash purchased from Advion Interchim, and/or with pre-coated Merck preparative TLC plates (silica gel 60G F254, 20 × 20 cm). Nuclear magnetic resonance (NMR) spectra were recorded using Bruker 200 MHz and/or 300 MHz spectrometers. ^1H and ^{13}C chemical shifts are reported in ppm downfield of tetramethylsilane and referenced to residual solvent peak ($CDCl_3$: δH = 7.26 and δC = 77.16). $^{19}F\{^1H\}$ chemical shifts are reported in ppm and referenced to $CFCl_3$ (δF = 0.00). Multiplicities are reported using the following abbreviations: s = singlet, d = doublet, t = triplet, q = quartet, p = pentuplet, m = multiplet, b = broad. All quantitative ^{19}F NMR spectra were decoupled with ^1H and were recorded in the presence of a known quantity of internal reference (trifluoromethoxybenzene). High-resolution mass spectrometry (HRMS) was recorded on a Mass Spectrometer XEVO-QTOF.

General procedure 1: Selective oxidation of trifluoromethyl aryl sulfides

We used a method previously developed by our group [1] to selectively oxidize trifluoromethyl aryl sulfides to the corresponding sulfoxides.

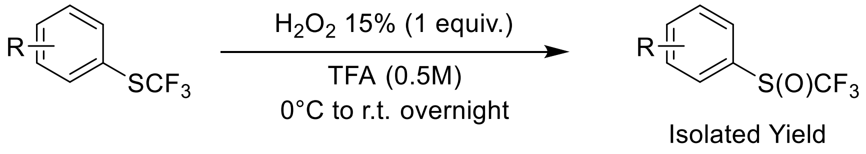

The sulfide (1 mmol, 1 equiv) and TFA (2 mL, 0.5 M) were added into a 5 mL round-bottomed flask equipped with a magnetic stirring bar. Then, 15 mass % aqueous solution of H_2O_2 (1 equiv) were added very slowly through an automatic injector during about 90 min at room temperature. The reaction mixture was stirred overnight at rt. The reaction mixture was poured into water, neutralized with solid $NaHCO_3$ to pH 6–7, then extracted with diethyl ether. The combined organic phase was dried over $MgSO_4$, concentrated and purified by silica gel chromatography, if necessary.

1-(Trifluoromethoxy)-4-((trifluoromethyl)sulfinyl)benzene (1b)

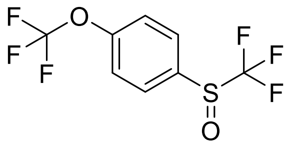

$C_8H_4F_6O_2S$
278.17

Reaction carried out following the general procedure 1 using (4-(trifluoromethoxy)phenyl)(trifluoromethyl)sulfane (524 mg, 2 mmol). Purified by flash chromatography (petroleum ether 100%), affording the product as a slightly yellow oil (261 mg, 0.94 mmol). Yield = 47%. The NMR data matched with the literature.[2]

* **^{1}H NMR (300 MHz, $CDCl_3$) δ (ppm):** 7.86 (dd, *J* = 9, 1 Hz, 2H), 7.46 (dd, *J* = 9, 1 Hz, 2H).

* **^{19}F NMR (282 MHz, $CDCl_3$) δ (ppm):** -58.25 (s, 3F), -74.85 (s, 3F).

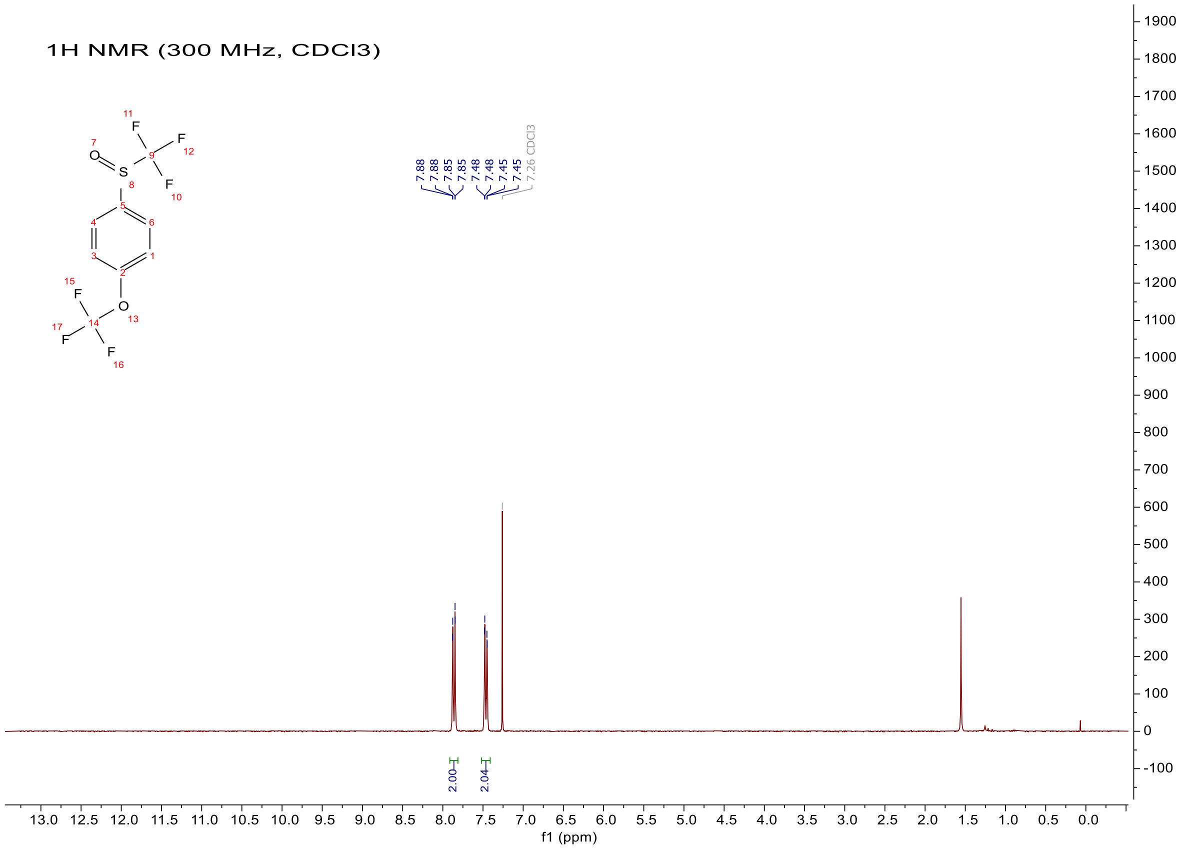
1H NMR (300 MHz, CDCl3)
7.88
7.88
7.85
7.85
7.48
7.48
7.45
7.45
7.26 CDCl3
2.00
2.04
f1 (ppm)

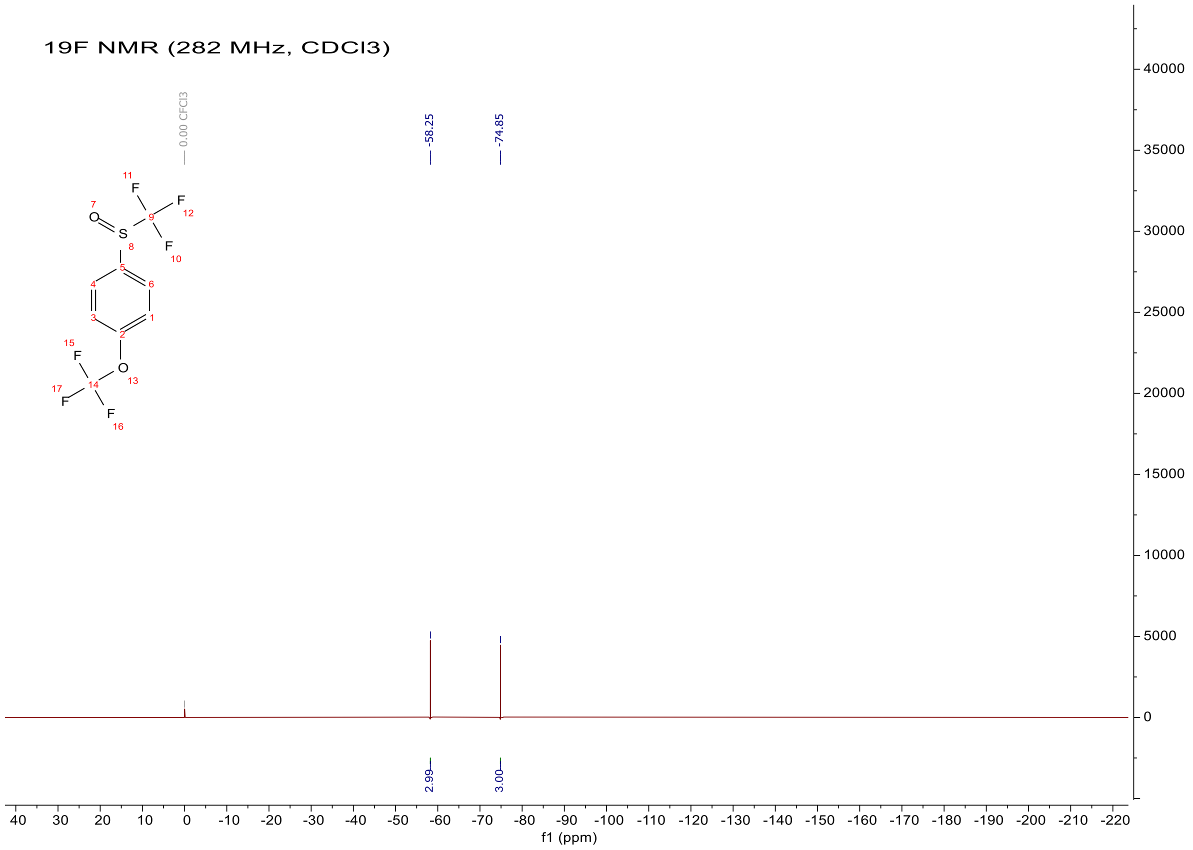
19F NMR (282 MHz, CDCl3)
0.00 CFCl3
-58.25
-74.85
2.99
3.00
f1 (ppm)

1-(Chloromethyl)-4-((trifluoromethyl)sulfinyl)benzene (1c)

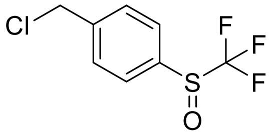

$C_8H_6ClF_3OS$
242.64

Reaction carried out following the general procedure 1 using (4-(chloromethyl)phenyl)(trifluoromethyl)sulfane (453 mg, 2 mmol). Purified by flash chromatography (petroleum ether/diethyl ether 90:10), affording the product as an orange oil (306 mg, 1.26 mmol). Yield = 63%.

* **^{1}H NMR (300 MHz, $CDCl_3$) δ (ppm):** 7.8 (d, ^{3}J = 8 Hz, 2H); 7.64 (d, ^{3}J = 8 Hz, 2H); 4.66 (s, 2H)

* **^{13}C NMR (75 MHz, $CDCl_3$) *δ* (ppm):** 143.3; 135.6; 129.7; 126.4, 124.7 (q, *J* = 336Hz), 44.9

* **^{19}F NMR (282 MHz, $CDCl_3$) δ (ppm):** -74.83

* **HRMS ASAP+TOF**: Calculated for $C_{16}H_{13}Cl_2F_6O_2S_2$[2M+H]$^{+}$: 484.9638; Found [2M+H]$^{+}$: 484.9260

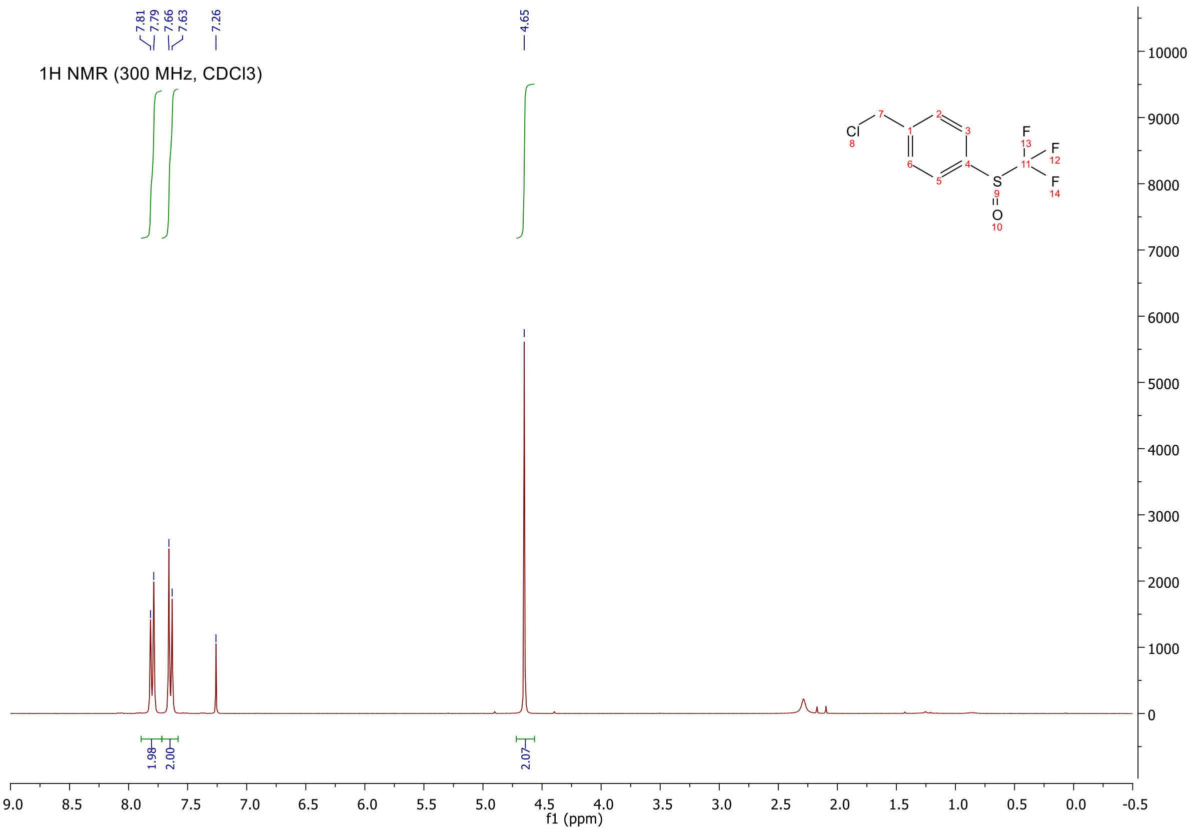
1H NMR (300 MHz, CDCl3)
7.81
7.79
7.66
7.63
7.26
4.65
1.98
2.00
2.07
f1 (ppm)

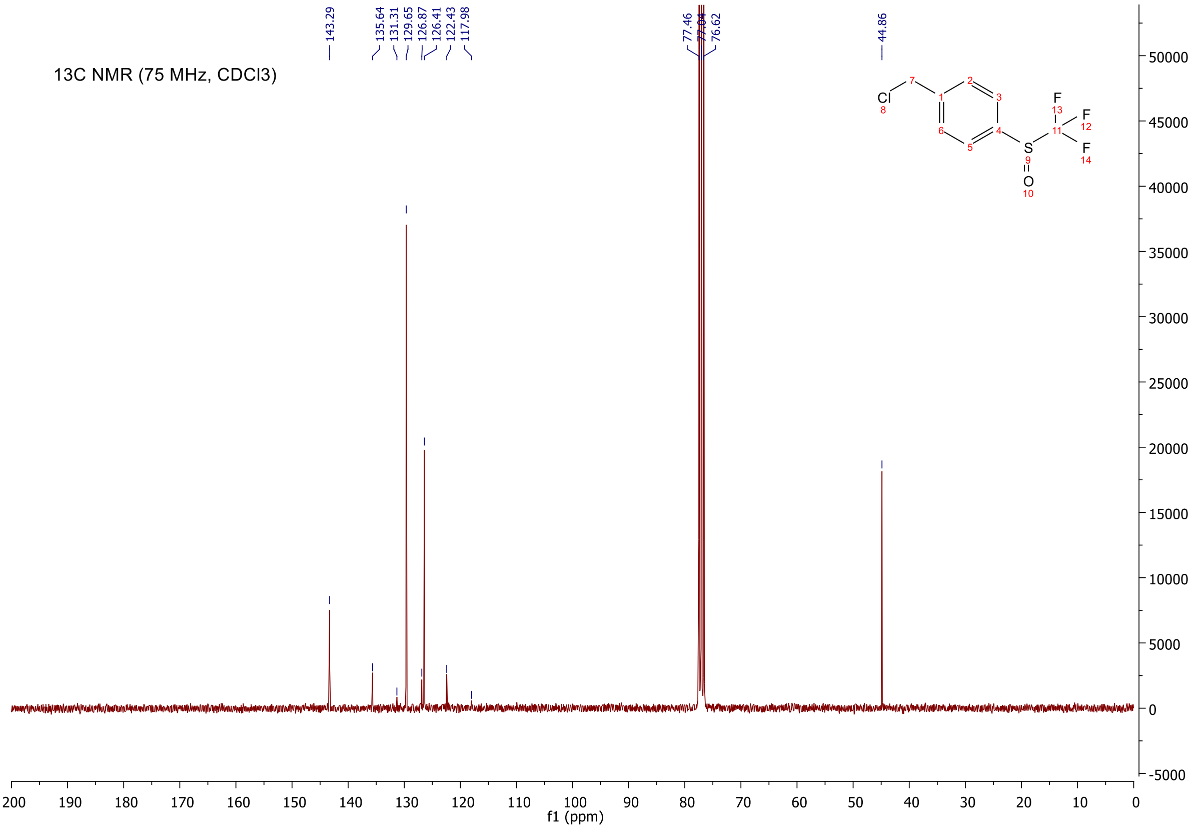
13C NMR (75 MHz, CDCl3)
143.29
135.64
131.31
129.65
126.87
126.41
122.43
117.98
77.46
77.04
76.62
44.86
f1 (ppm)

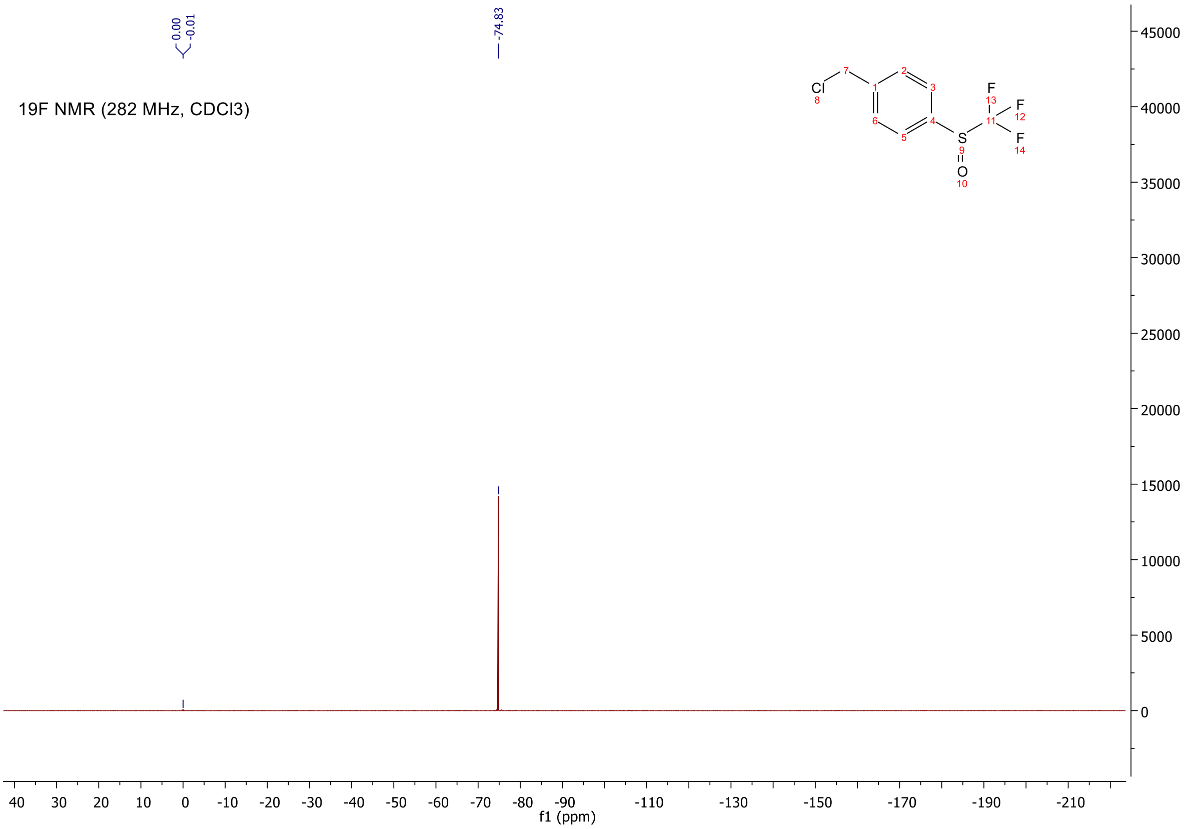

0.00
-0.01
-74.83
19F NMR (282 MHz, CDCl3)
Cl
F
F
F
S
O
45000
40000
35000
30000
25000
20000
15000
10000
5000
0
40
30
20
10
0
-10
-20
-30
-40
-50
-60
-70
-80
-90
-110
-130
-150
-170
-190
-210
f1 (ppm)

4-Bromo-1-((trifluoromethyl)sulfinyl)benzene (1d)

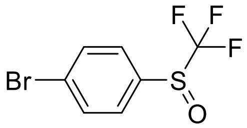

$C_7H_4BrF_3OS$
271,9118

Reaction carried out following the general procedure 1 using (4-(bromophenyl)(trifluoromethyl)sulfane (511 mg, 2 mmol). Purified by flash chromatography (petroleum ether/diethyl ether 90:10), affording the product as an orange oil (482 mg, 1.78 mmol). Yield = 89%. The NMR data matched with the literature.[1]

* **^{1}H NMR (200 MHz CDCl$_3$)** : d 7.81 (d, ^3J = 7,5 Hz, 2H); 7.65 (d, ^3J = 7,5 Hz, 2H); 4.66 (s, 2H)

* **^{19}F NMR (282 MHz, CDCl$_3$) δ (ppm):** -74.81

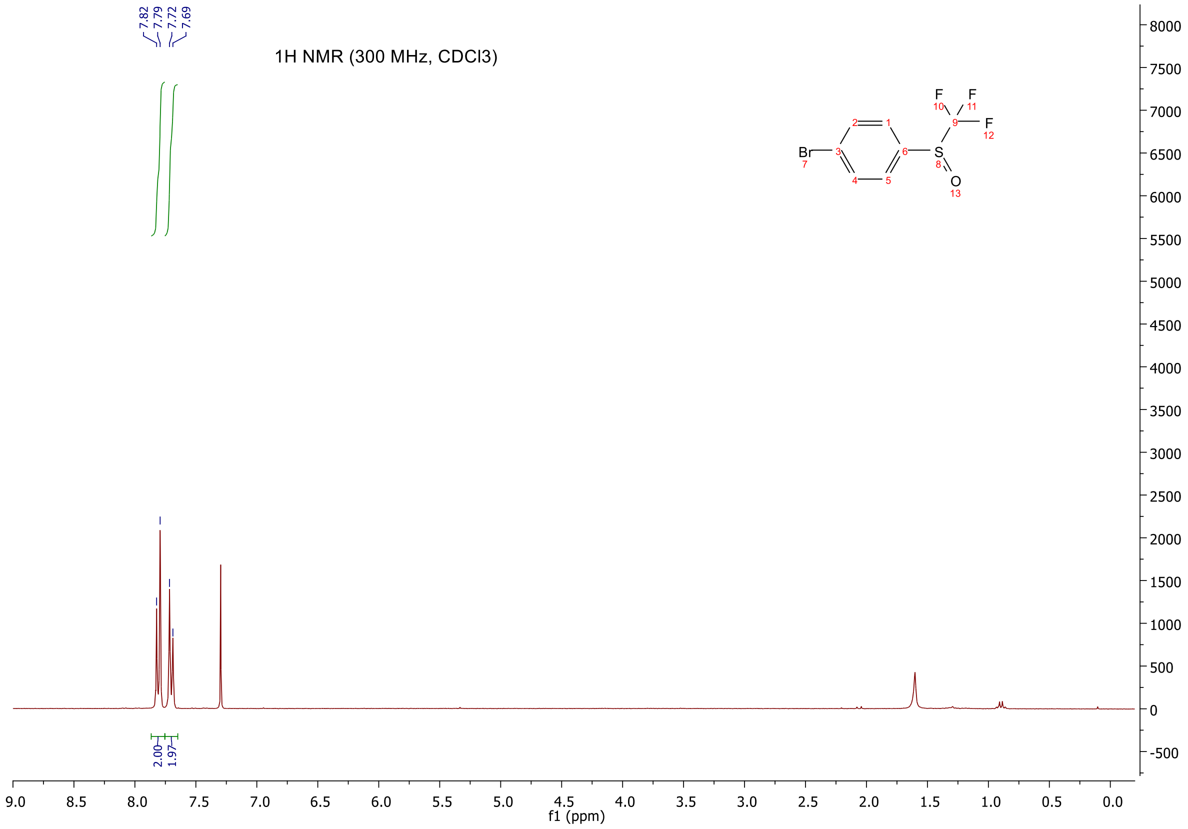

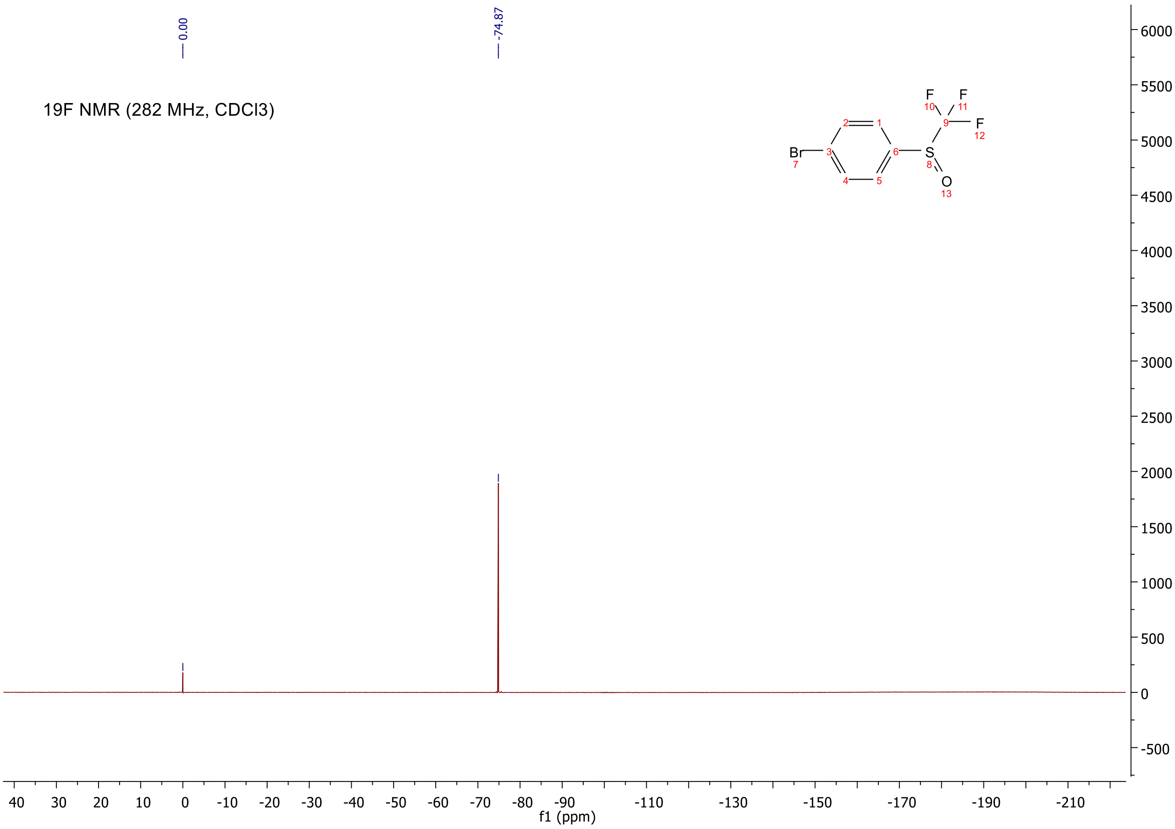

0.00
-74.87
19F NMR (282 MHz, CDCl3)
F
F
F
Br
S
O
6000
5500
5000
4500
4000
3500
3000
2500
2000
1500
1000
500
0
-500
40 30 20 10 0 -10 -20 -30 -40 -50 -60 -70 -80 -90 -110 -130 -150 -170 -190 -210
f1 (ppm)

2-Fluoro-4-methyl-1-((trifluoromethyl)sulfinyl)benzene (1e)

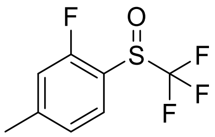

$C_8H_6F_4OS$
226.19

Reaction carried out following the general procedure 1 using (2-fluoro-4-methylphenyl)(trifluoromethyl)sulfane (385 mg, 1.83 mmol). Purified by flash chromatography (Petroleum ether/diethyl ether 80:20), affording the product as an orange oil (149 mg, 0.66 mmol). Yield = 36%.

* **HRMS ASAP+TOF**: Calculated for $C_{16}H_{13}F_8O_2S_2$[2M+H]$^+$: 453.0229; Found [2M+H]$^+$: 453.0235

* **^{1}H NMR (300 MHz, CDCl$_3$) δ (ppm):** 7.83 (t, *J* = 7 Hz, 1H), 7.25 (d, *J* = 8 Hz, 1H), 7.03 (d, *J* = 12 Hz, 1H), 2.46 (s, 3H).

* **^{13}C NMR (75 MHz, CDCl$_3$) *δ* (ppm):** 159.8 (d, *J* = 252 Hz), 147.6 (d, *J* = 8 Hz), 127.0 (d, *J* = 2 Hz), 126.6 (d, *J* = 3 Hz), 125.0 (qd, *J* = 336, 3 Hz), 120.2 (qd, *J* = 16, 2 Hz), 117.0 (d, *J* = 20 Hz), 21.8

* **^{19}F NMR (282 MHz, CDCl$_3$) δ (ppm):** -75.03 (d, *J* = 9 Hz), -114.39 (q, *J* = 9 Hz).

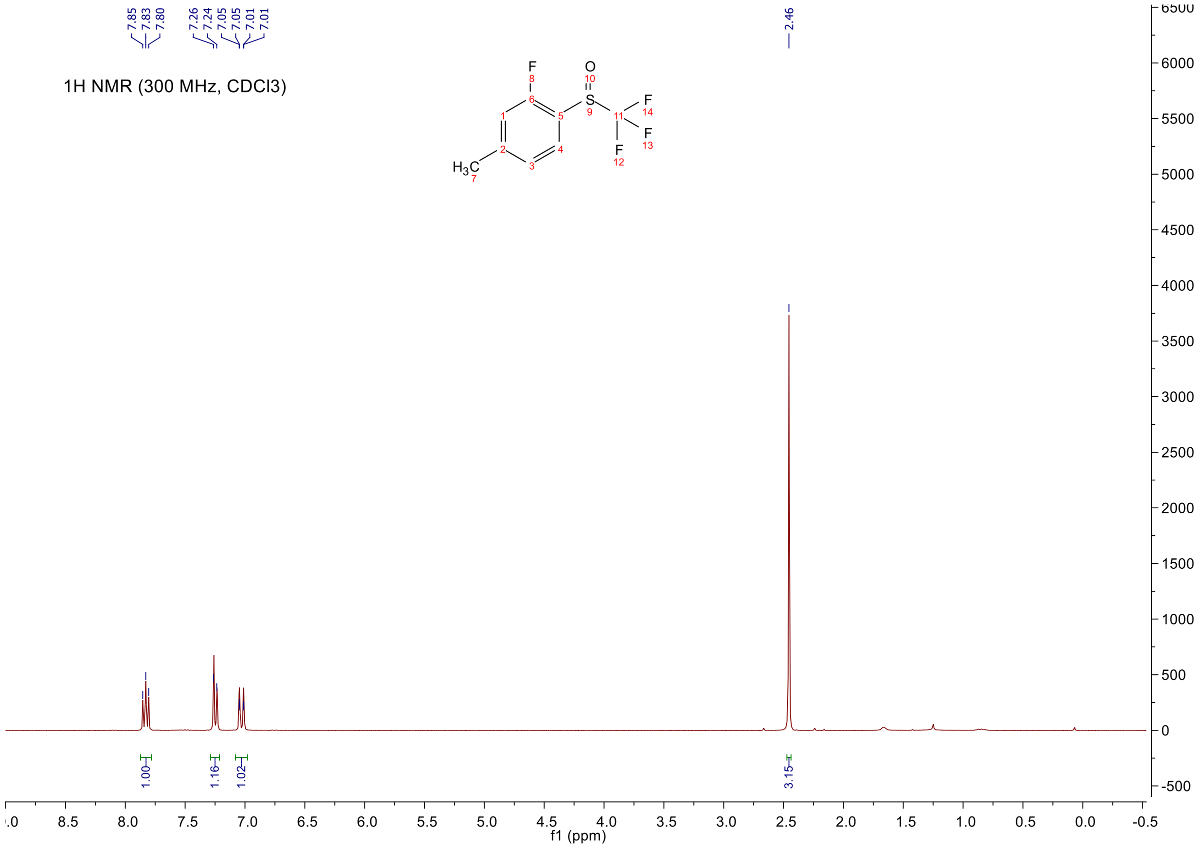

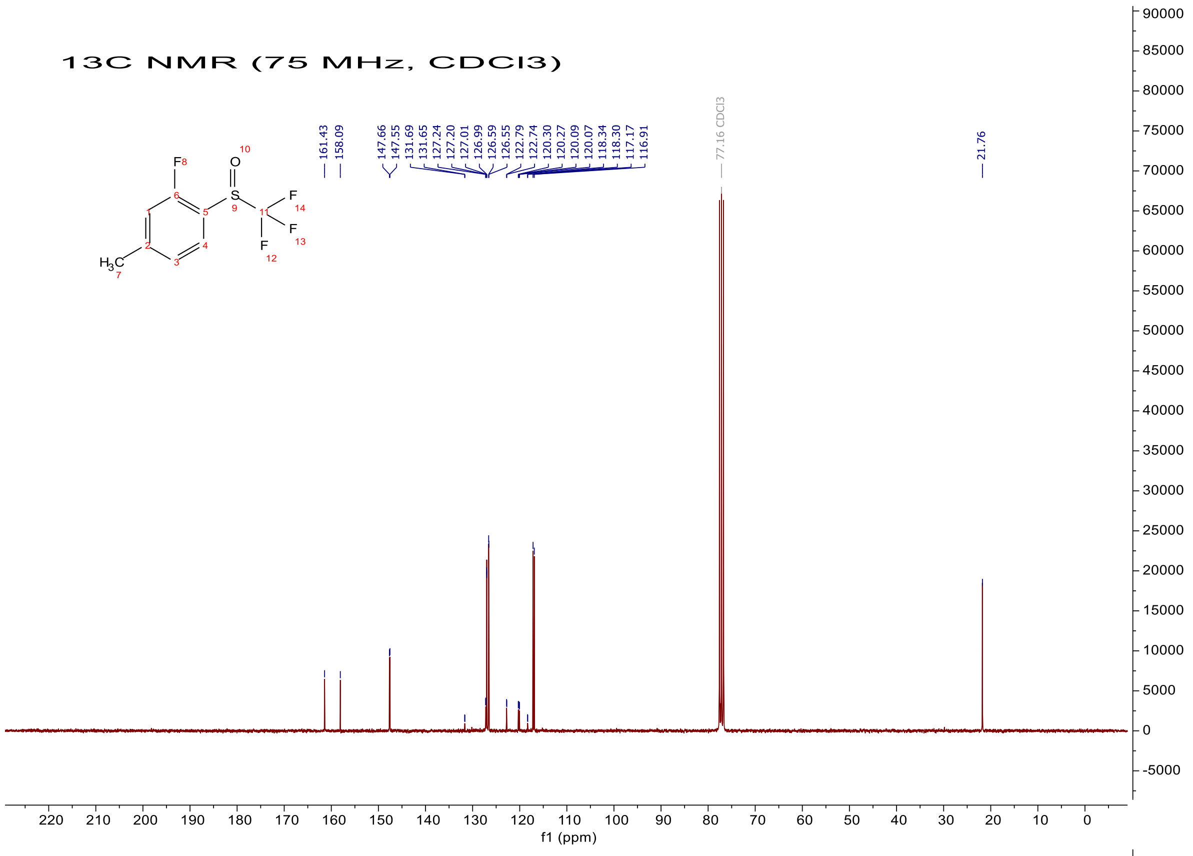
13C NMR (75 MHz, CDCl3)
161.43
158.09
147.66
147.55
131.69
131.65
127.24
127.20
127.01
126.99
126.59
126.55
122.79
122.74
120.30
120.27
120.09
120.07
118.34
118.30
117.17
116.91
77.16 CDCl3
21.76
f1 (ppm)

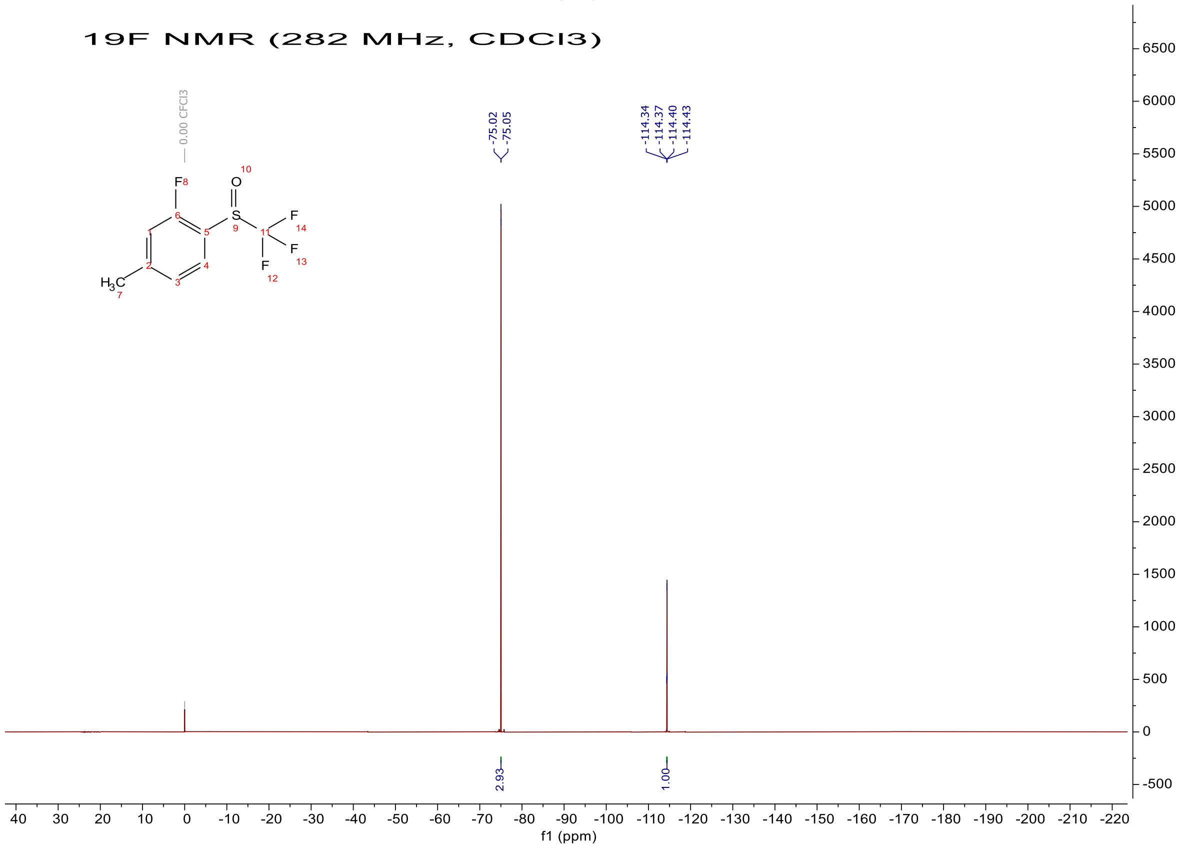
19F NMR (282 MHz, CDCl3)
0.00 CFCl3
-75.02
-75.05
-114.34
-114.37
-114.40
-114.43
2.93
1.00
f1 (ppm)

3-Methyl-1-((trifluoromethyl)sulfinyl)benzene (1f)

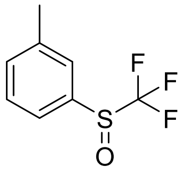

$C_8H_7F_3OS$
208,017

Reaction carried out following the general procedure 1 using (3-methylphenyl)(trifluoromethyl)sulfane (210 mg, 1.15 mmol). Purified by flash chromatography (petroleum ether/diethyl ether 80:20), affording the product as an orange oil (58 mg, 0.28 mmol). Yield = 25%. The NMR data matched with the literature.[7]

* **^{1}H NMR (300 MHz, CDCl$_3$) δ (ppm):** 7.66 – 7.60 (m, 2H), 7.55 – 7.47 (m, 2H), 7.69 – 7.62 (m, 1H), 4.06 (d, *J* = 1 Hz, 2H).

* **^{19}F NMR (282 MHz, CDCl$_3$) δ (ppm):** -75.00 (s, 3F)

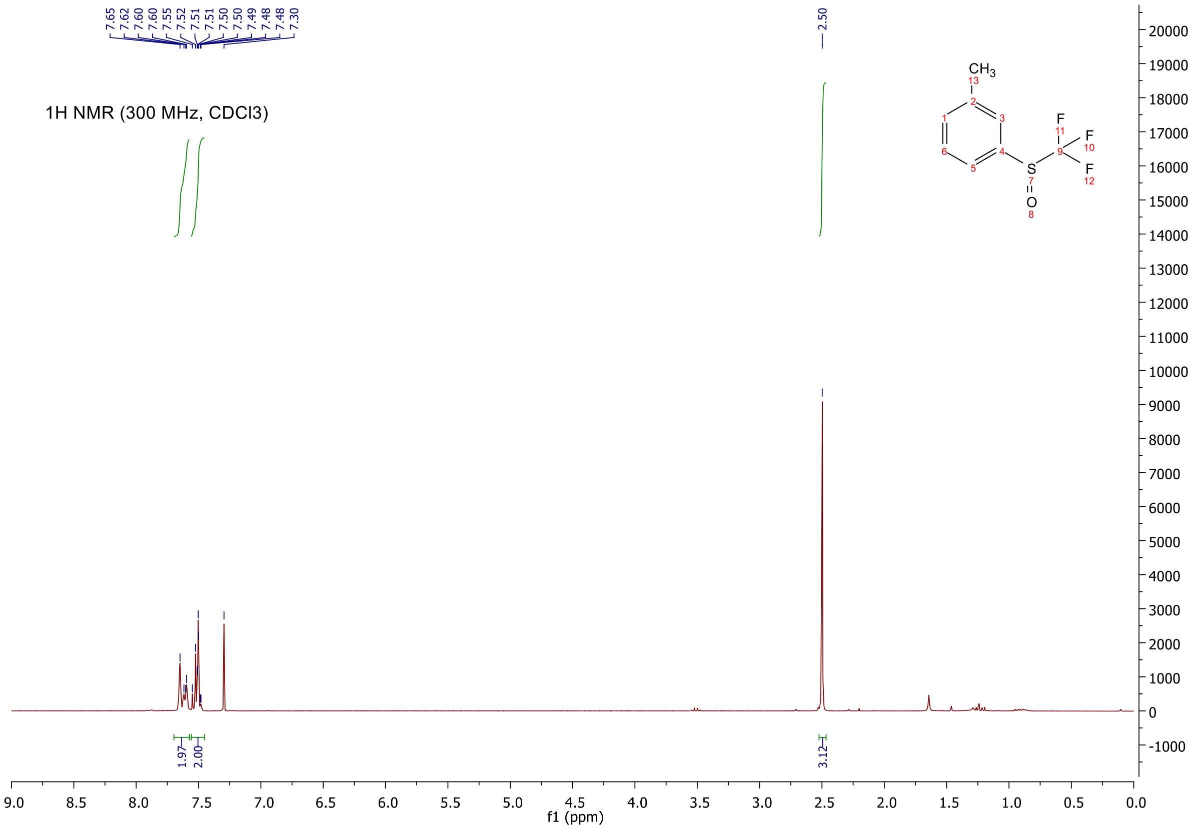

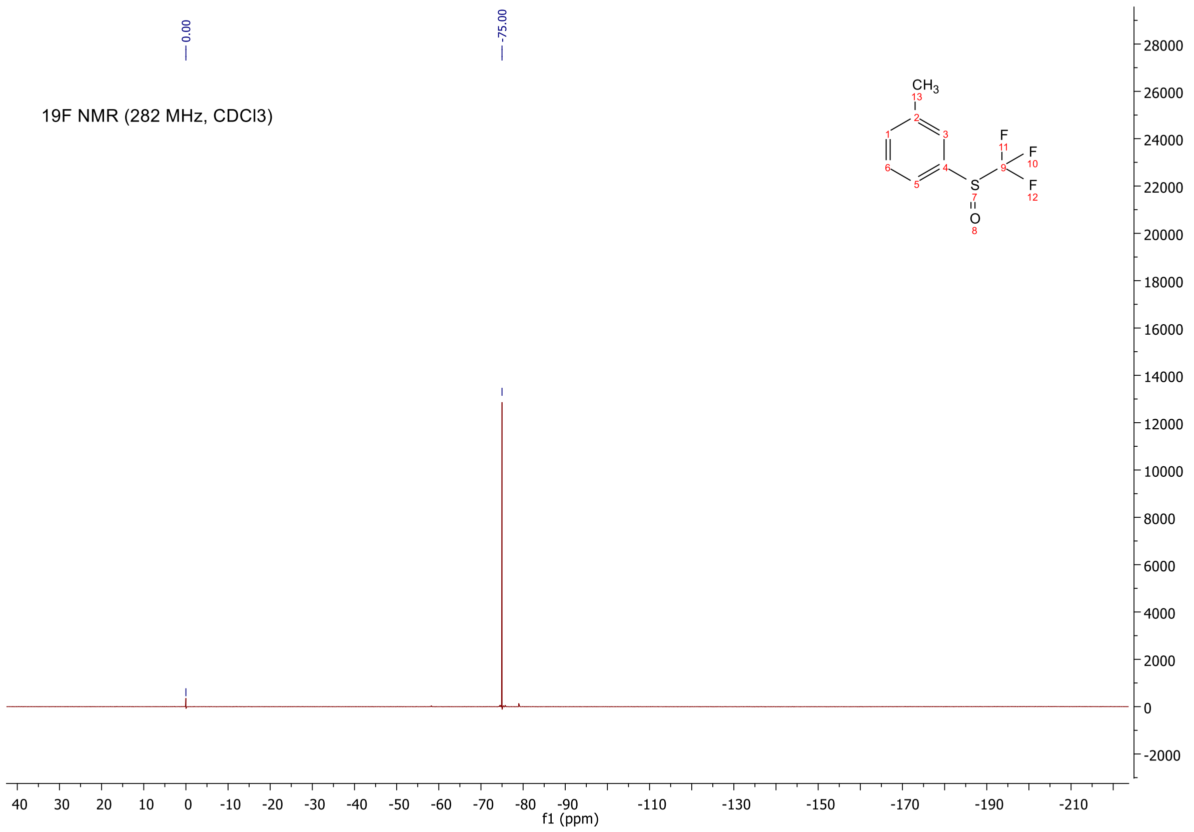

0.00
-75.00
19F NMR (282 MHz, CDCl3)
CH3
F
F
F
S
O
28000
26000
24000
22000
20000
18000
16000
14000
12000
10000
8000
6000
4000
2000
0
-2000
40 30 20 10 0 -10 -20 -30 -40 -50 -60 -70 -80 -90 -110 -130 -150 -170 -190 -210
f1 (ppm)

2-(2-((Trifluoromethyl)sulfinyl)phenyl)acetonitrile (1g)

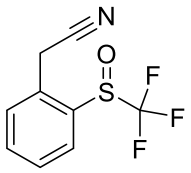

$C_9H_6F_3NOS$
233.21

Reaction carried out following the general procedure 1 using (2-fluoro-4-methylphenyl)(trifluoromethyl)sulfane (457 mg, 2.10 mmol). Purified by flash chromatography (petroleum ether/diethyl ether 33:67), affording the product as an orange oil (343 mg, 1.47 mmol). Yield = 70%. Purity = 96%. The impurity was the corresponding sulfone.

* **HRMS ASAP+TOF**: Calculated for $C_9H_6F_3NOS[M+H]^+$: 234.0200; Found $[M+H]^+$: 234.0190

* **^{1}H NMR (300 MHz, $CDCl_3$) δ (ppm):** 8.02 – 7.94 (m, 1H), 7.75 – 7.71 (m, 2H), 7.69 – 7.62 (m, 1H), 4.06 (d, *J* = 1 Hz, 2H).

* **^{13}C NMR (75 MHz, $CDCl_3$) *δ* (ppm):** 134.4, 133.6 (q, *J* = 2 Hz), 130.9, 130.8, 129.8, 127.8, 125.2 (q, *J* = 336 Hz), 116.2, 20.3 (q, *J* = 2 Hz).

* **^{19}F NMR (282 MHz, $CDCl_3$) δ (ppm):** -73.25 (s, 3F)

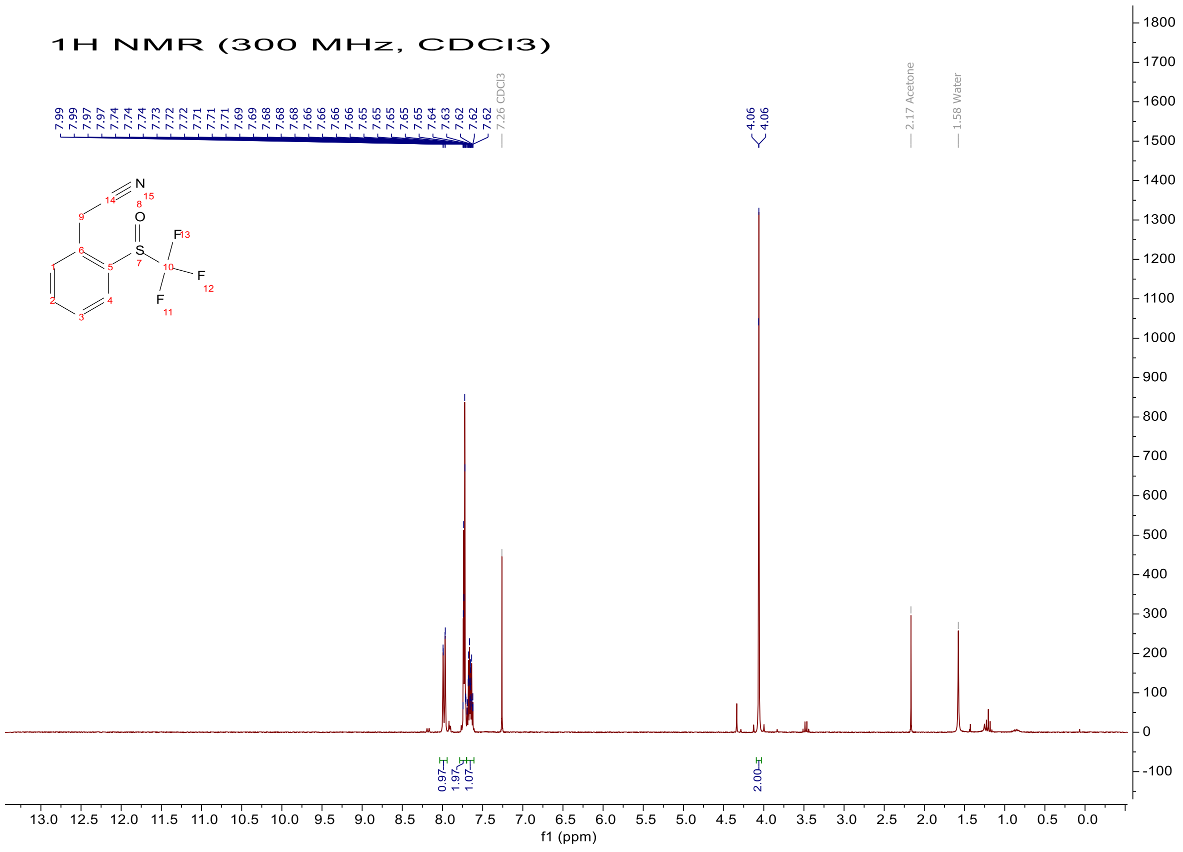
1H NMR (300 MHz, CDCl3)
7.26 CDCl3
2.17 Acetone
1.58 Water
f1 (ppm)

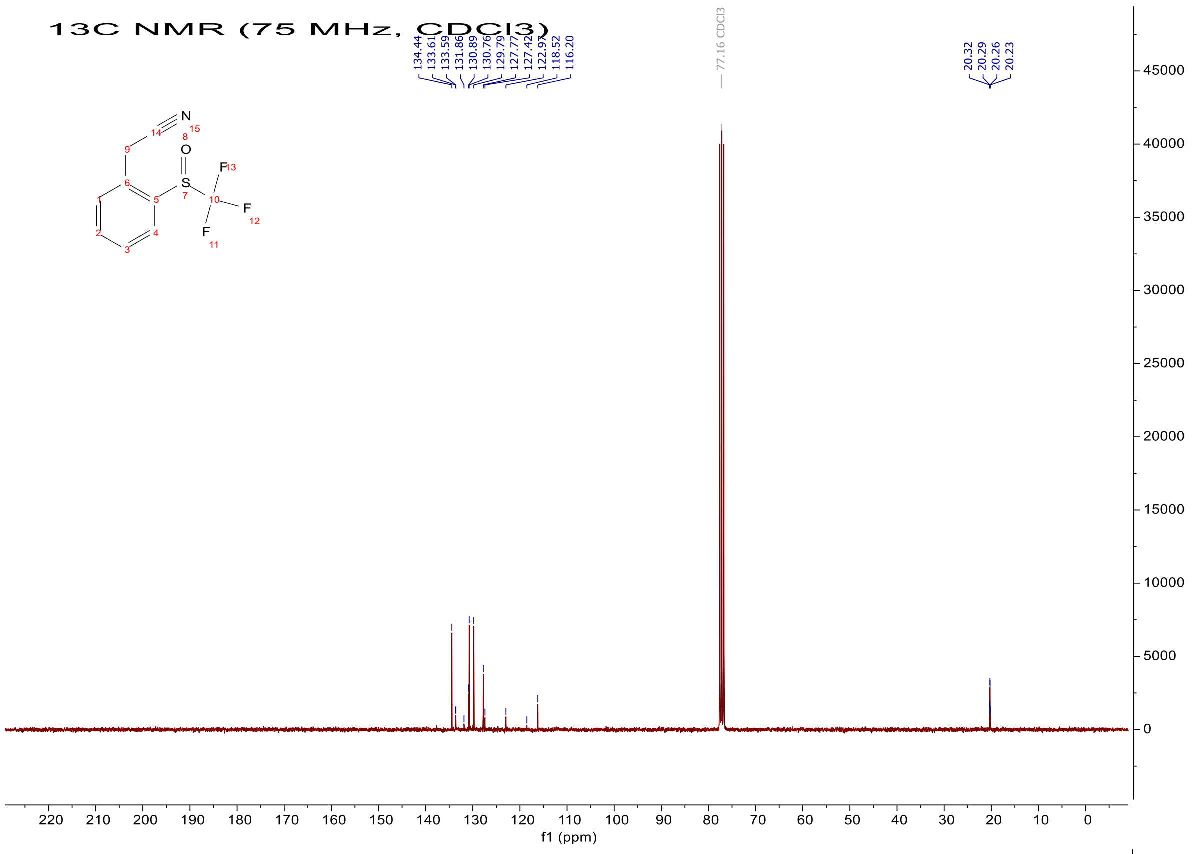
13C NMR (75 MHz, CDCl3)
134.44
133.61
133.59
131.86
130.89
130.76
129.79
127.77
127.42
122.97
118.52
116.20
77.16 CDCl3
20.32
20.29
20.26
20.23
N
O
S
F
F
F
f1 (ppm)

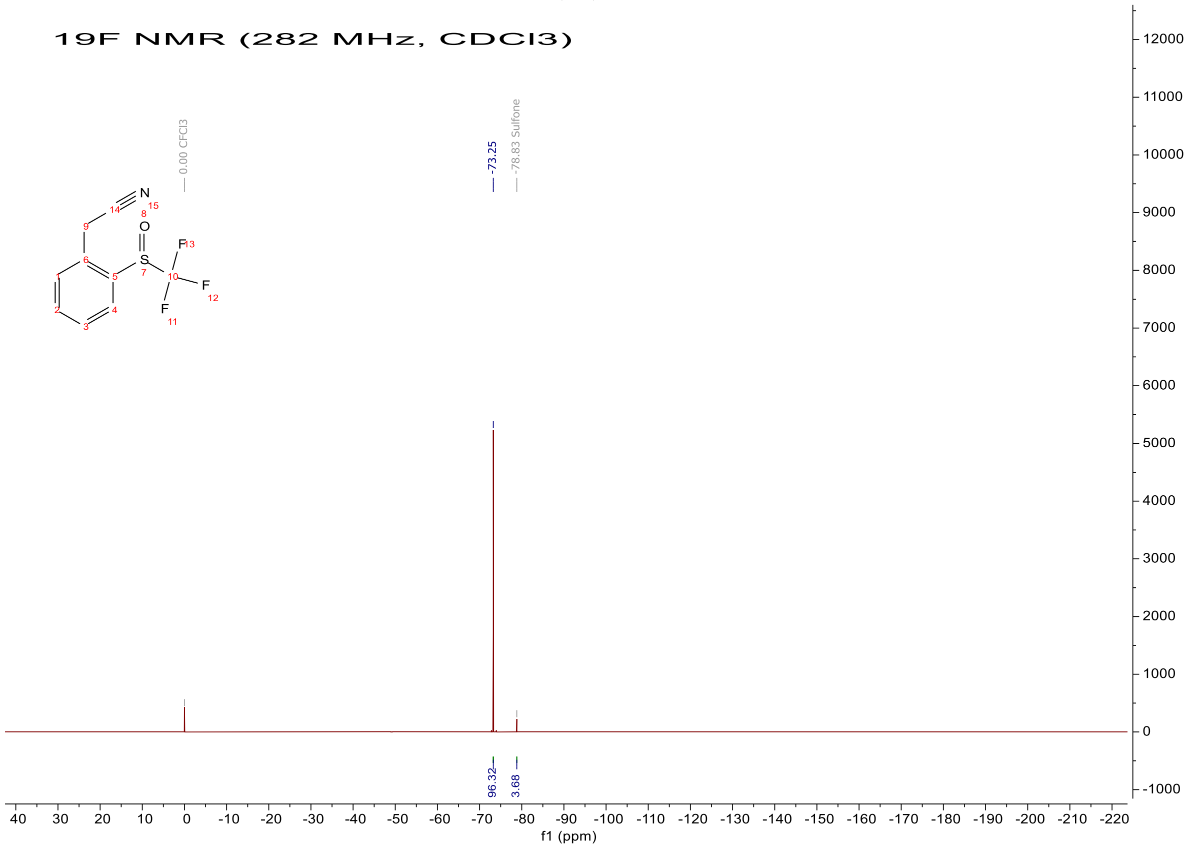
19F NMR (282 MHz, CDCl3)
0.00 CFCl3
-73.25
-78.83 Sulfone
96.32
3.68
N
O
S
F
F
F
f1 (ppm)

1-Bromo-4-((difluoromethyl)sulfinyl)benzene (1h)

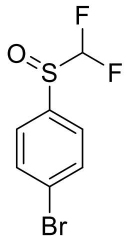

$C_7H_5BrF_2OS$
255.08

Reaction carried out following the general procedure 1 using (4-bromophenyl)(difluoromethyl)sulfane (478 mg, 2 mmol). Purified by flash chromatography (petroleum ether/diethyl ether 92:8), affording the product as a slightly yellow oil (281 mg, 1.1 mmol). Yield = 55%. The NMR data matched with the literature.[4]

* **^{1}H NMR (300 MHz, $CDCl_3$) δ (ppm):** 7.78 – 7.72 (m, 2H), 7.63 – 7.56 (m, 2H), 6.05 (t, *J* = 55 Hz, 1H).

* **^{19}F NMR (282 MHz, $CDCl_3$) δ (ppm):** -119.17 (dd, *J* = 262, 55 Hz, 1F), -120.31 (dd, *J* = 262, 55 Hz, 1F).

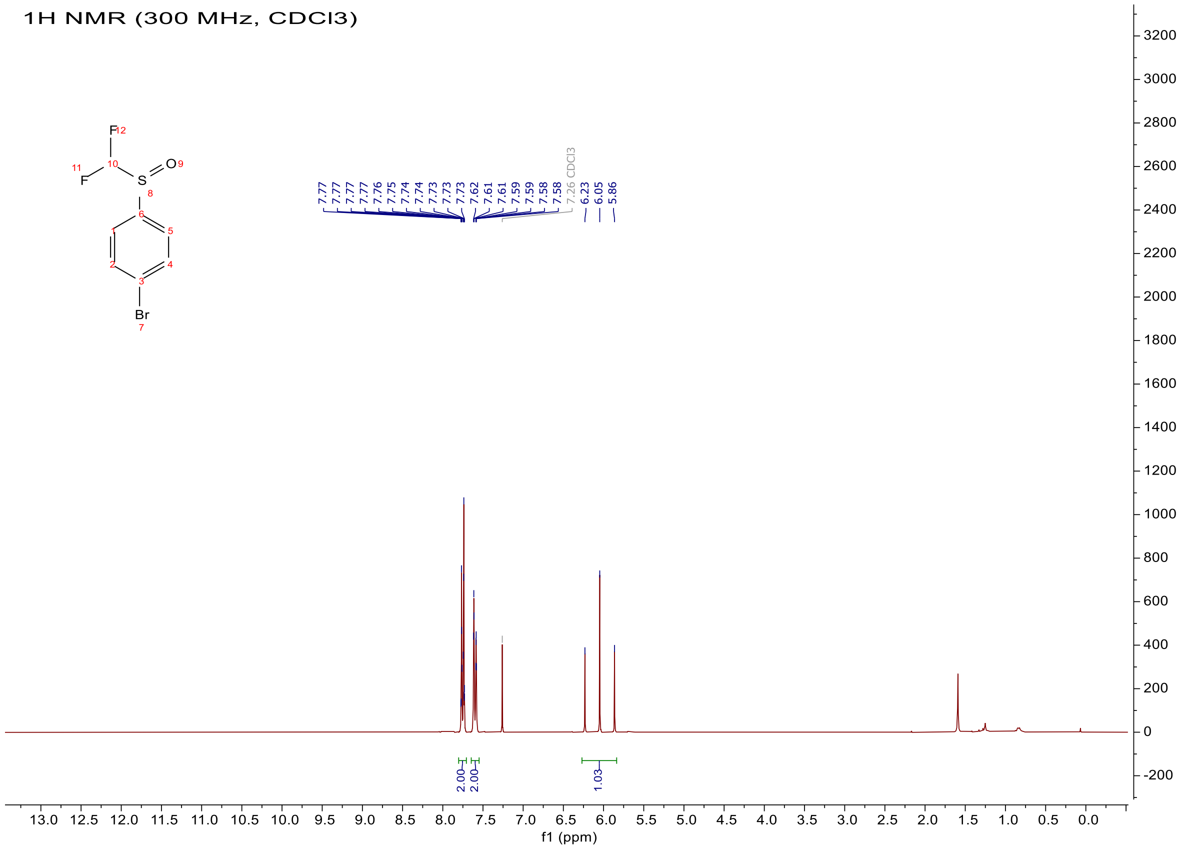
1H NMR (300 MHz, CDCl3)
7.77
7.77
7.77
7.77
7.76
7.75
7.74
7.74
7.73
7.73
7.73
7.73
7.62
7.61
7.61
7.59
7.59
7.58
7.58
7.26 CDCl3
6.23
6.05
5.86
2.00
2.00
1.03
f1 (ppm)

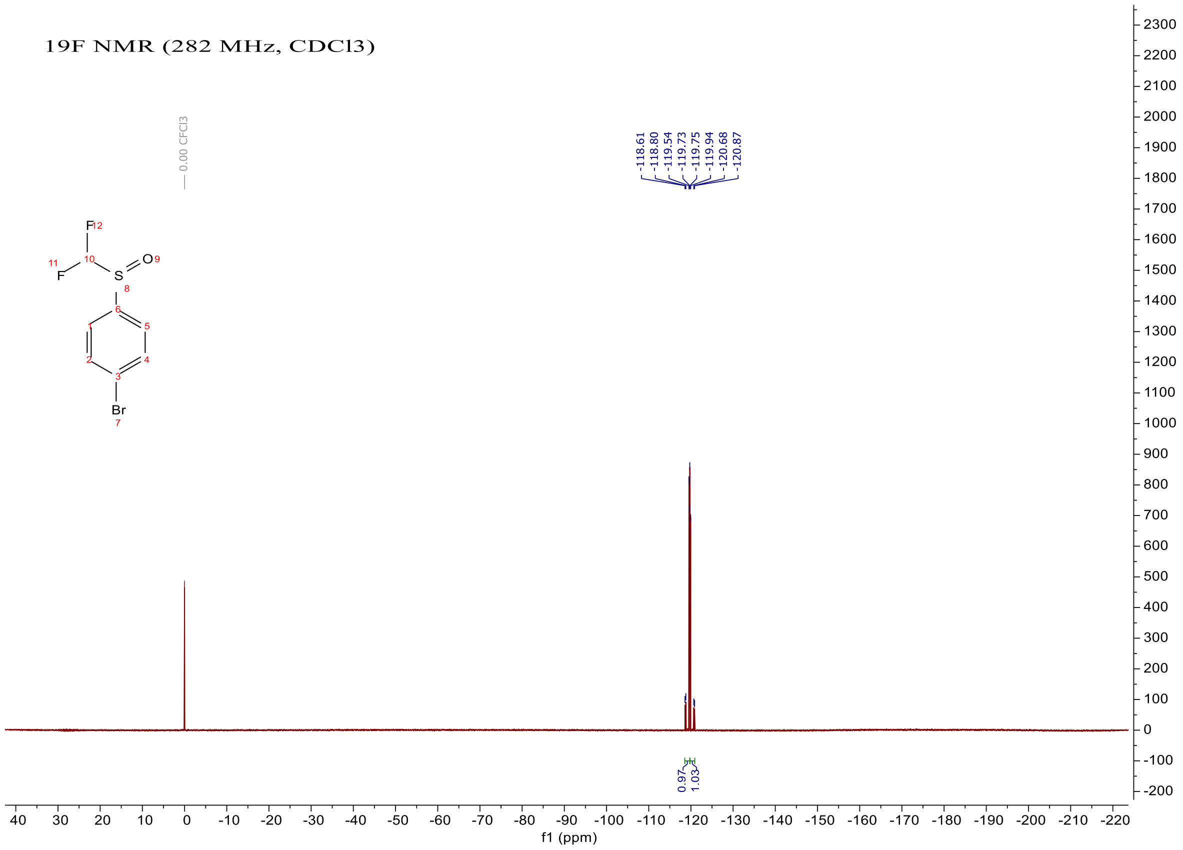
19F NMR (282 MHz, CDCl3)
0.00 CFCl3
-118.61
-118.80
-119.54
-119.73
-119.75
-119.94
-120.68
-120.87
0.97
1.03
f1 (ppm)

((Difluoromethyl)sulfinyl)benzene (1i)

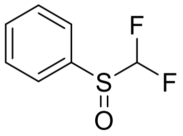

$C_7H_6F_2OS$
176.18

Reaction carried out following the general procedure 1 using (difluoromethyl)(phenyl)sulfane (320 mg, 2 mmol). Purified by flash chromatography (petroleum ether/diethyl ether 92:8), affording the product as a slightly yellow oil (127 mg, 1.1 mmol). Yield = 36%. The NMR data matched with the literature.[5]

* **^{1}H NMR (300 MHz, CDCl3) δ (ppm):** 7.77 – 7.69 (m, 2H), 7.67 – 7.56 (m, 3H), 6.04 (t, *J* = 55 Hz, 1H).

* **^{19}F NMR (282 MHz, CDCl3) δ (ppm):** -119.72 (d, *J* = 55 Hz, 1F), -119.76 (d, *J* = 55 Hz, 1F),

1H NMR (300 MHz, CDCl3)

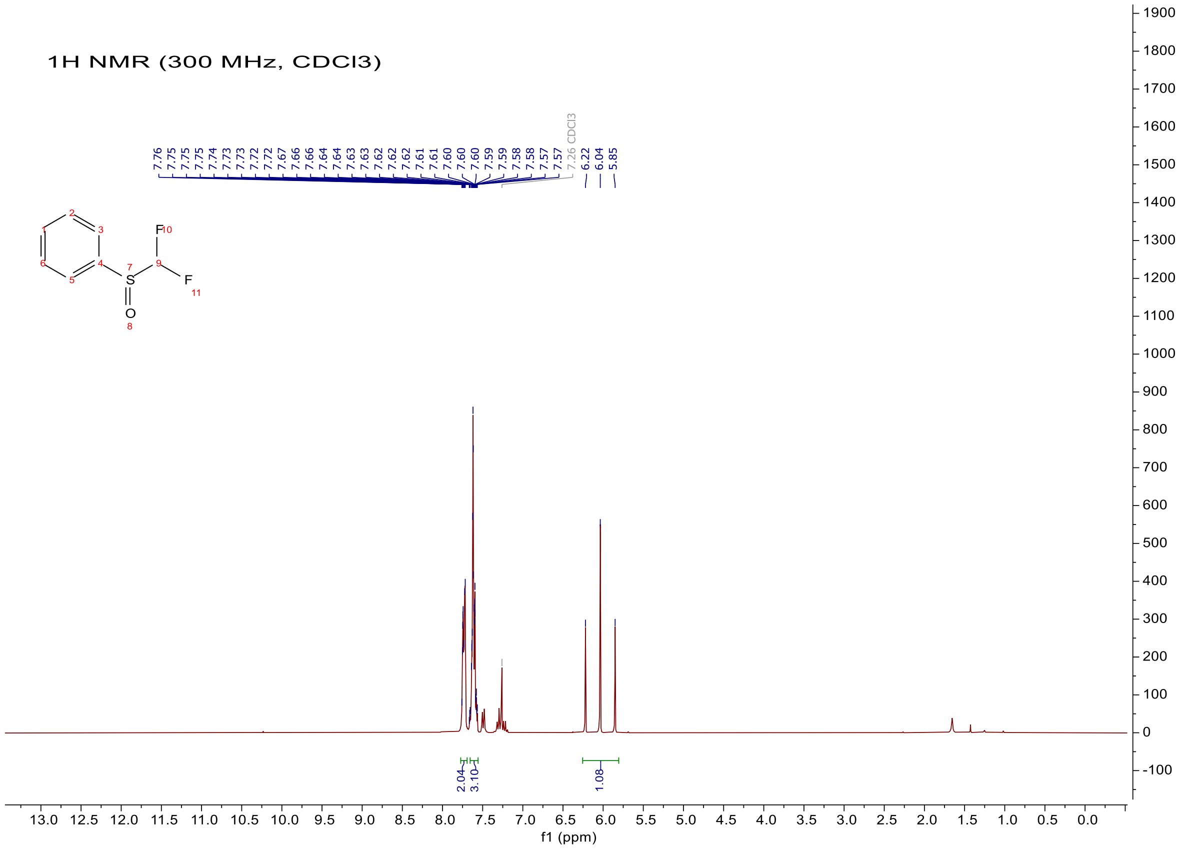

19F NMR (282 MHz, CDCl3)

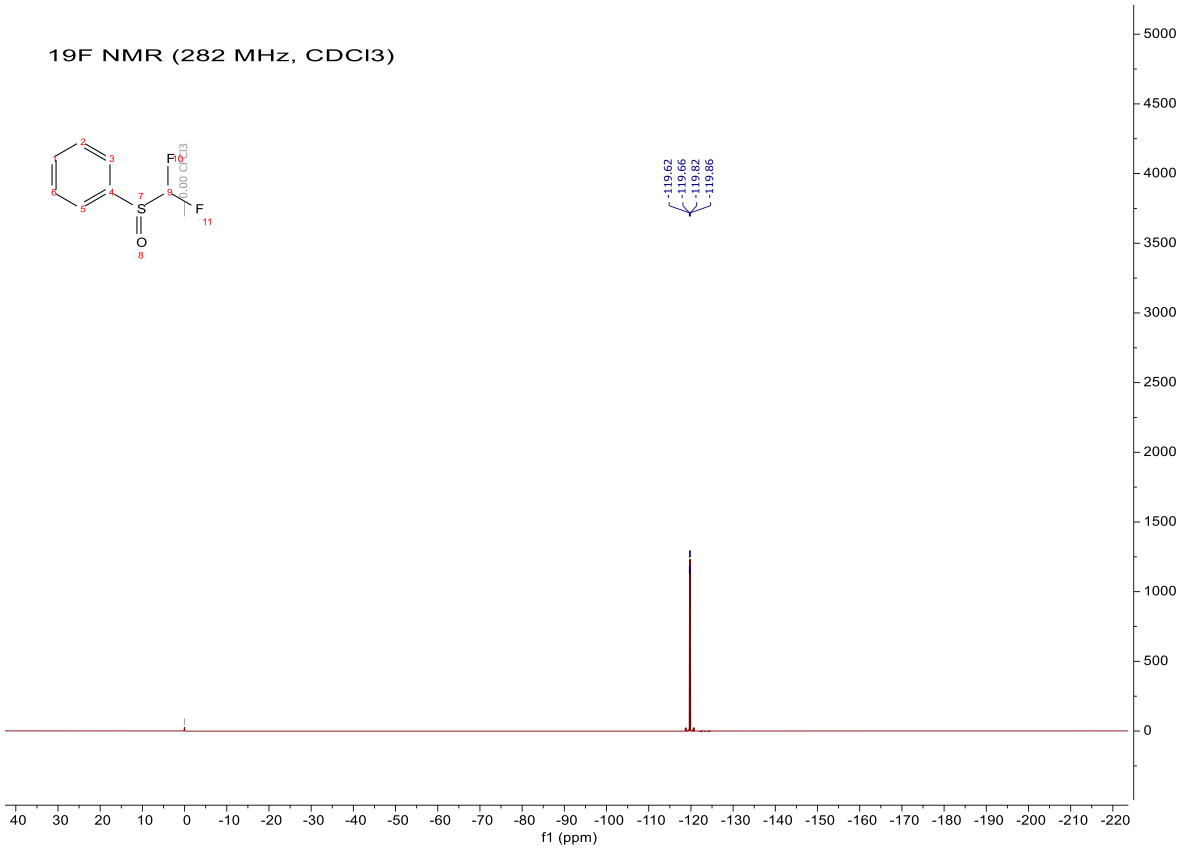

((Bromodifluoromethyl)sulfinyl)benzene (1j)

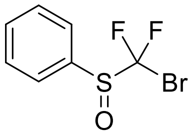

$C_7H_5BrF_2OS$
255.08

Reaction carried out following the general procedure 1 using (bromodifluoromethyl)(phenyl)sulfane (807 mg, 3.37 mmol). Purified by flash chromatography (petroleum ether/diethyl ether 92:8), affording the product as a slightly yellow oil (609 mg, 2.39 mmol). Yield = 71%. The NMR data matched with the literature.[1]

* **^{1}H NMR (200 MHz, $CDCl_3$) δ (ppm):** 7.80 (d, J = 7 Hz, 2H), 7.68 – 7.53 (m, 3H).

* **^{19}F NMR (188 MHz, $CDCl_3$) δ (ppm):** -53.42 (d, J = 145 Hz, 1F), -55.54 (d, J = 145 Hz, 1F).

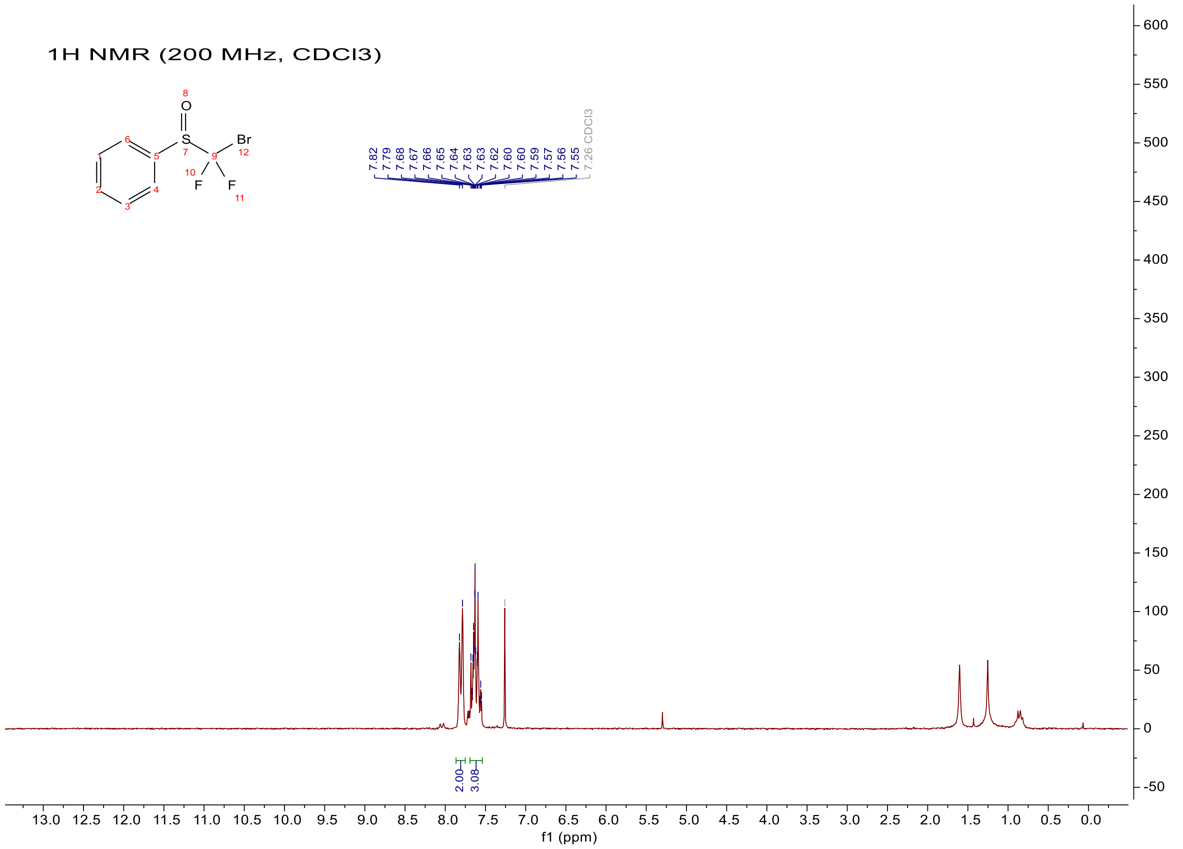
1H NMR (200 MHz, CDCl3)
7.82
7.79
7.68
7.67
7.66
7.65
7.64
7.63
7.63
7.62
7.60
7.59
7.57
7.56
7.55
7.26 CDCl3
2.00
3.08
f1 (ppm)

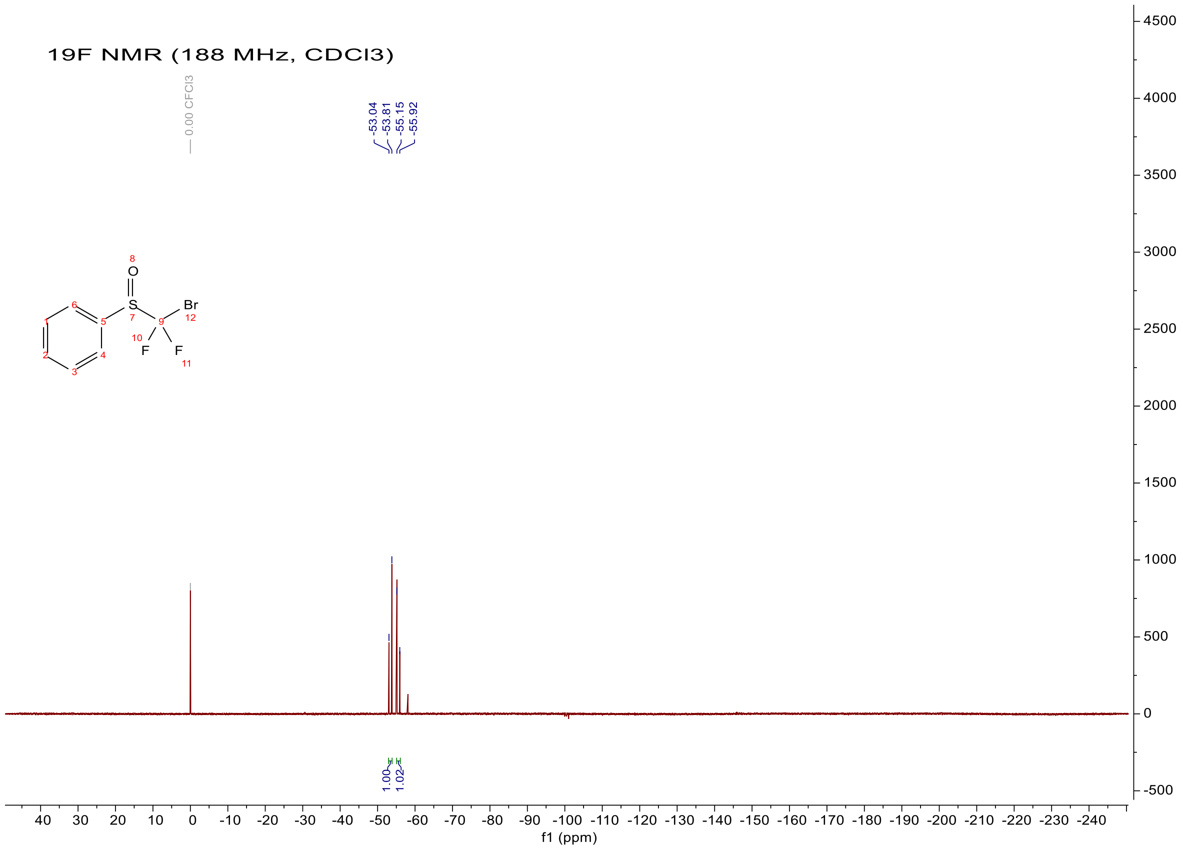
19F NMR (188 MHz, CDCl3)
0.00 CFCl3
-53.04
-53.81
-55.15
-55.92
1.00
1.02
f1 (ppm)

((Trifluoromethyl)seleninyl)benzene (1k)

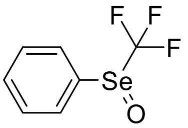

$C_7H_5F_3OSe$
241,9458

To a round-bottomed flask equipped with a magnetic stirring bar was added the phenylselenocyanate (990 mg, 5 mmol, 1 equiv) followed by 15 mL of dry THF. $TMSCF_3$ (10 mmol, 2 equiv) was added at 0 °C and TBAF (1 mmol, 0.2 equiv) was added dropwise. The mixture was then allowed to warm to room temperature and stirred until full conversion (the conversion was checked by ^{19}F NMR with $PhOCF_3$ as internal standard). Then, Oxone® (10 mmol, 2 equiv) was added at 0 °C, followed by 15 mL of water. The mixture was then allowed to warm to room temperature and stirred at room temperature for 3 h (the conversion was checked by ^{19}F NMR with $PhOCF_3$ as internal standard). The mixture was then filtered through a pad of silica and concentrated under vaccum. The mixture was then portioned with DCM (5 mL) and water (5 mL), the organic layer was washed with brine (10 mL) and water (10 mL), dried over $MgSO_4$, filtered and concentrated under vacuum. The crude residue was purified by column chromatography (*n*-pentane/EtOAC 70:30) to afford **1k** as a yellow solid (943.4 mg, 3.9 mmol, 78%). The NMR data matched with the literature.[1]

* **^{1}H NMR (200 MHz, $CDCl_3$) δ (ppm):** 7.82 (d, J = 6 Hz, 2H), 7.64 – 7.56 (m, 3H).

* **^{19}F NMR (188 MHz, $CDCl_3$) δ (ppm):** -65.11 (s, 3F).

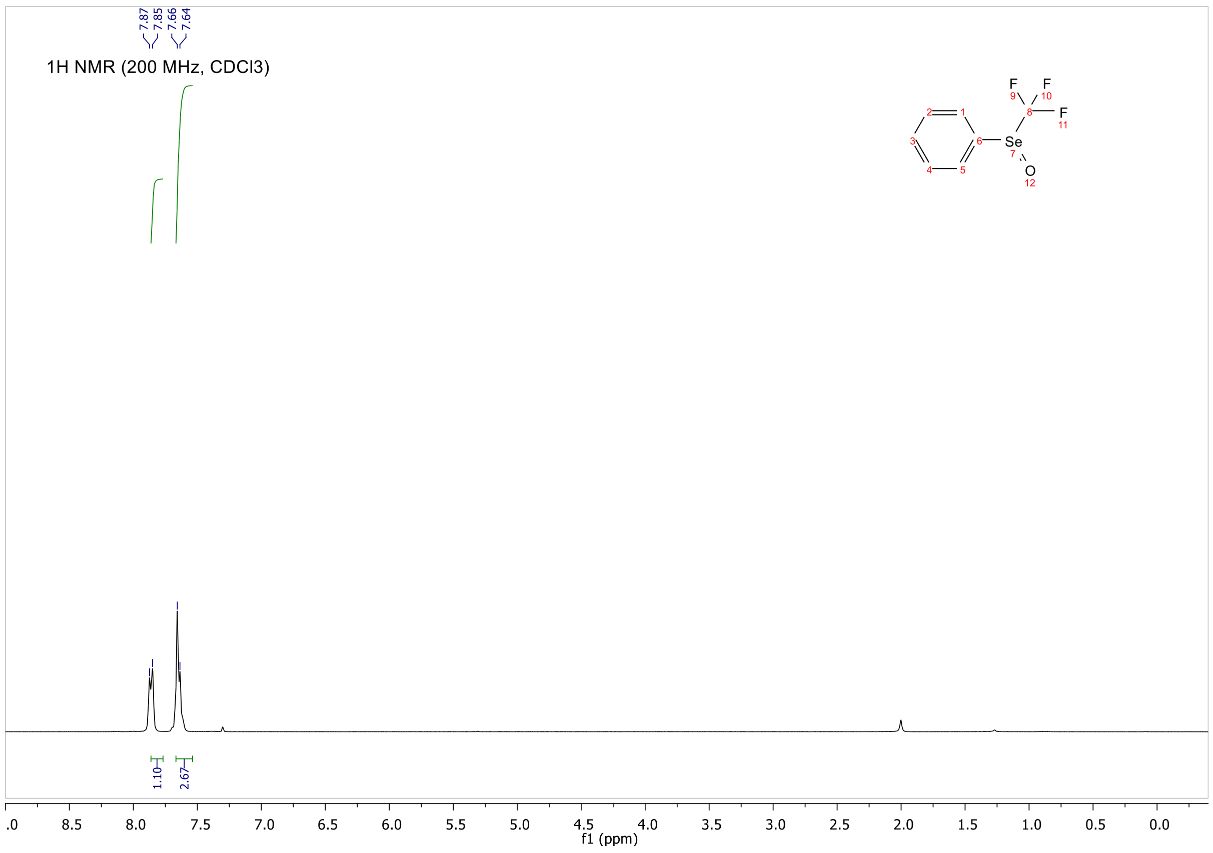
7.87
7.85
7.66
7.64
1H NMR (200 MHz, CDCl3)
1.10
2.67
f1 (ppm)

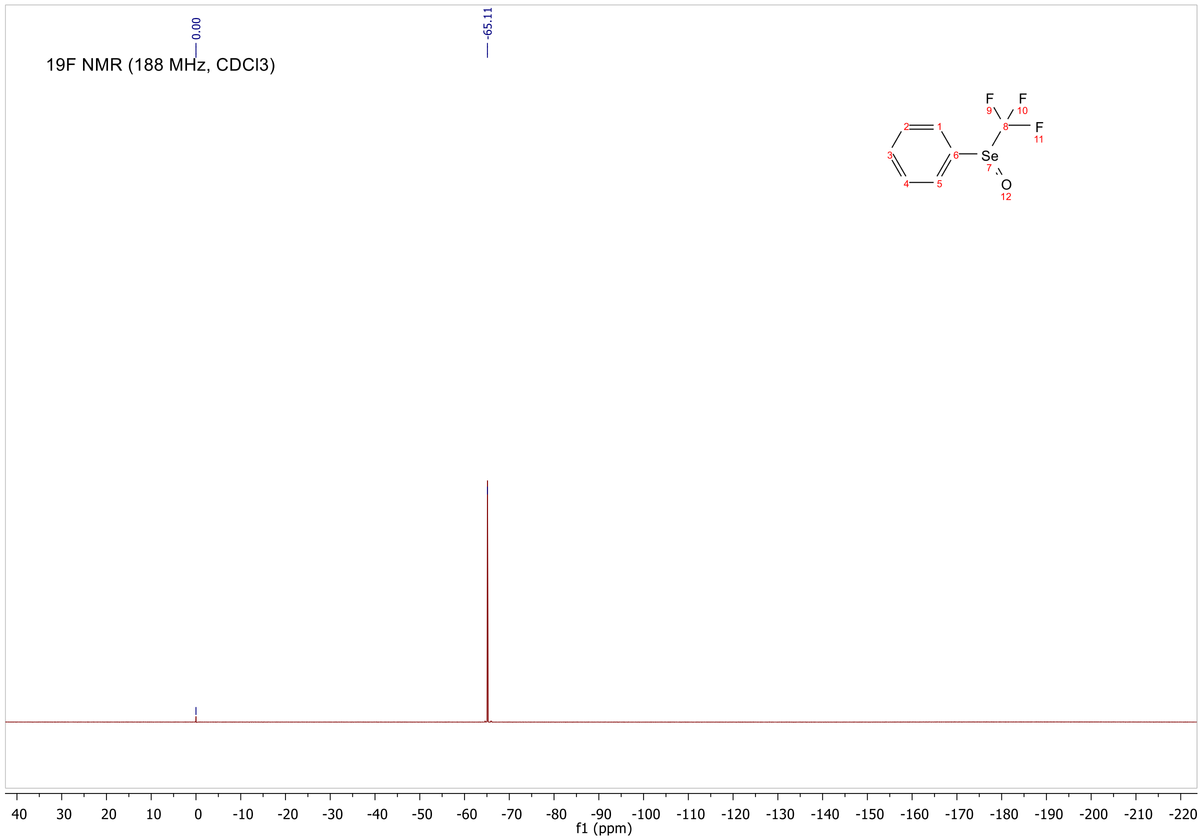
0.00
-65.11
19F NMR (188 MHz, CDCl3)
f1 (ppm)

General procedure 2: Rearrangement

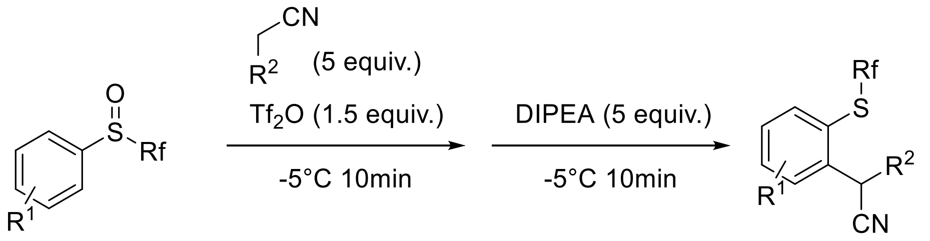

The sulfoxide (0.5 mmol, 1 equiv), nitrile (5 equiv) and Tf_2O (1.5 equiv) were added in the described order to a 5mL flask under an argon atmosphere, maintained at −5 °C. The reaction mixture was stirred for 10 min, then DIPEA (5 equiv) was slowly added to the flask with a syringe and the reaction was stirred for another 10 min. At the end of the reaction, 1 mL of chloroform and a known amount of trifluoromethoxybenzene were added to the flask in order to determine the ^{19}F NMR yield. To purify the product, the reaction mixture was mixed with a sufficient volume of a saturated NH_4Cl solution, then extracted 3 times with diethyl ether. The combined organic layers were dried over $MgSO_4$, filtered, concentrated under reduced pressure and purified by preparative TLC or flash chromatography

2-(2-((Trifluoromethyl)thio)phenyl)acetonitrile (2a)

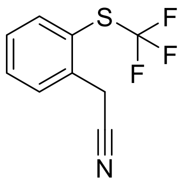

$C_9H_6F_3NS$
217.21

Reaction carried out following the general procedure 2 using ((trifluoromethyl)sulfinyl)benzene (97 mg, 0.5 mmol) and acetonitrile. NMR ^{19}F yield = 95%. Purified by preparative TLC (petroleum ether/diethyl ether 7:3) affording the product as a yellow oil (87 mg, 0.4 mmol). Yield = 79%.

Gram-Scale:

An oven-dried 100 mL round-bottomed flask was charged with argon gas, then ((trifluoromethyl)sulfinyl)benzene (2 g, 10.3 mmol, 1 equiv) and acetonitrile (5.4 mL, 103 mmol, 10 equiv) were added by syringe. The flask was cooled to and maintained at −5 °C with magnetic stirring throughout the whole process. Tf_2O (2.6 mL, 15.5 mmol, 1.5 equiv) was added to this flask through an automatic injector programmed at a debit of 0.26 mL/min. The reaction mixture was then stirred for 10 minutes and DIPEA (8.9 mL, 51.5 mmol, 5 equiv) was added to this flask through an automatic injector programmed at a debit of 0.89 mL/min. The reaction mixture was then stirred for a further 10 minutes. The mixture was diluted in chloroform (20 mL) and a known quantity of

trifluoromethoxylbenzene was added. ^{19}F NMR yield = 91%. The reaction was mixed with NH_4Cl (saturated solution, 30 mL), extracted with diethyl ether (20 mL × 3), the combined organic layer was dried on $MgSO_4$, concentrated, and purified by flash chromatography (petroleum ether/diethyl ether 7:3) affording the product as a yellow oil (1881mg, 8.66mmol). Yield = 84%.

The analytical data are in accordance with the literature [7].

* **HRMS ASAP+TOF**: Calculated for $C_{18}H_{13}F_6N_2S_2$ $[2M+H]^+$: 435.0424, Found $[2M+H]^+$: 435.0433

* **^1H NMR (300 MHz, $CDCl_3$) δ (ppm):** 7.76 (dd, *J* = 8, 1 Hz, 1H), 7.67 (dd, *J* = 8, 1 Hz, 1H), 7.57 (td, *J* = 8, 1 Hz, 1H), 7.43 (td, *J* = 8, 2 Hz, 1H), 4.06 (s, 2H).

* **^{13}C NMR (75 MHz, $CDCl_3$) *δ* (ppm):** 139.3, 136.2, 132.6, 130.0, 129.6, 129.3 (q, *J* = 309 Hz), 123.38 (q, *J* = 2 Hz), 117.3, 31.0

* **^{19}F NMR (282 MHz, $CDCl_3$) δ (ppm):** -42.83 (s)

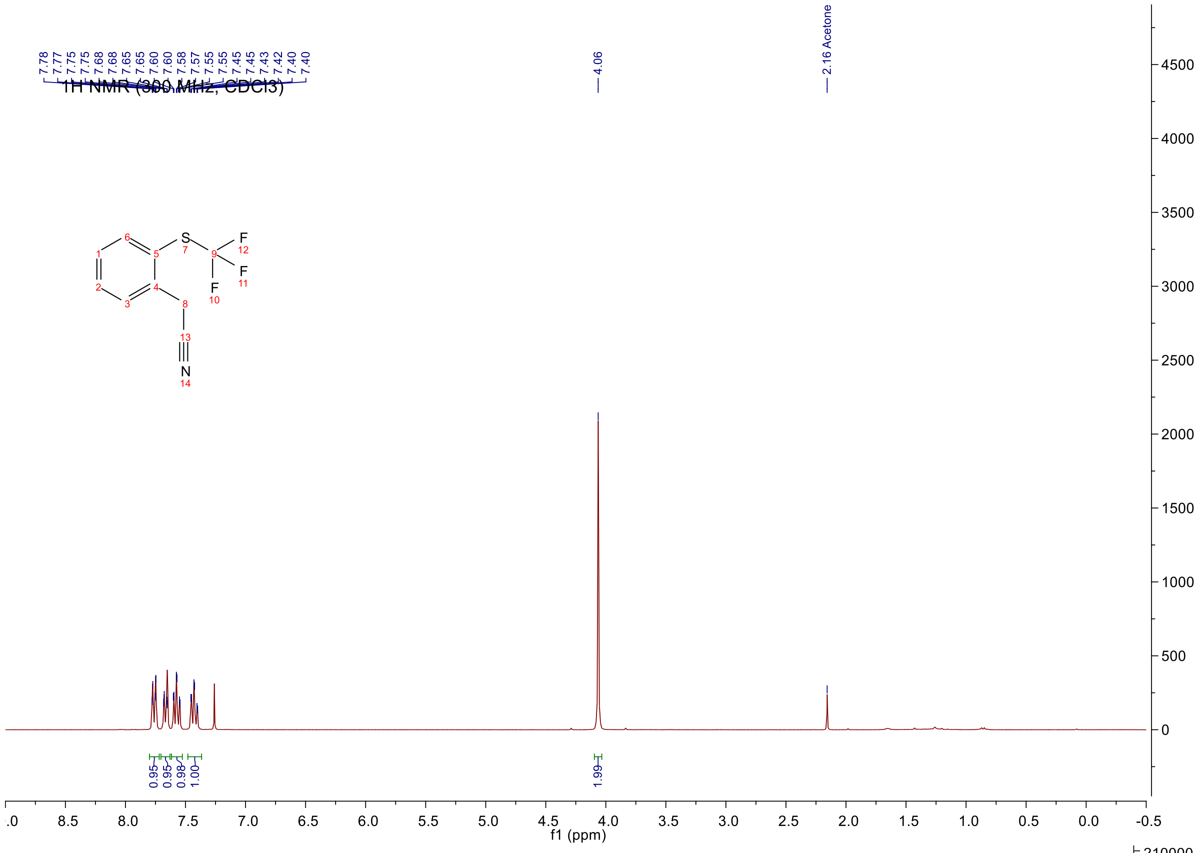
1H NMR (300 MHz, CDCl3)
7.78 7.77 7.75 7.75 7.68 7.65 7.65 7.60 7.60 7.58 7.57 7.55 7.55 7.45 7.45 7.43 7.42 7.40 7.40
4.06
2.16 Acetone
0.95 0.95 0.98 1.00
1.99
f1 (ppm)

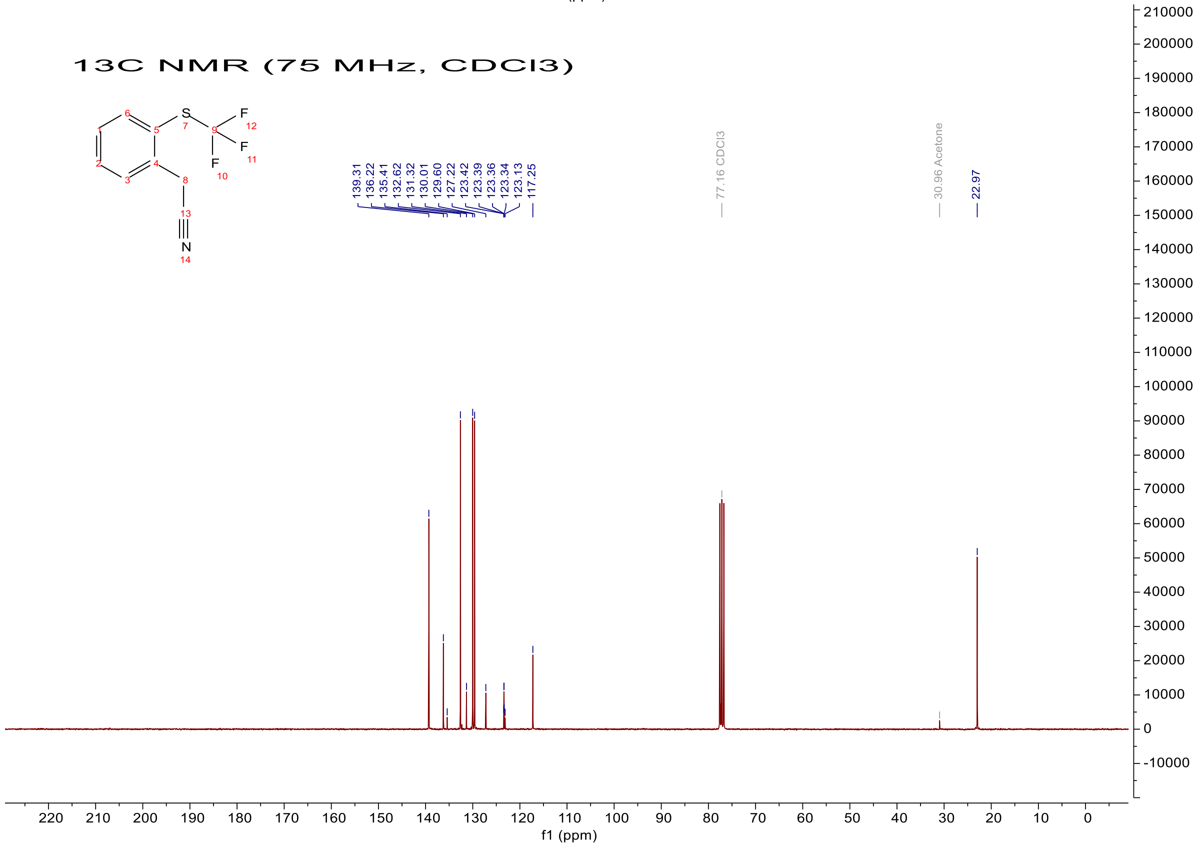
13C NMR (75 MHz, CDCl3)
139.31 136.22 135.41 132.62 131.32 130.01 129.60 127.22 123.42 123.39 123.36 123.34 123.13 117.25
77.16 CDCl3
30.96 Acetone
22.97
f1 (ppm)

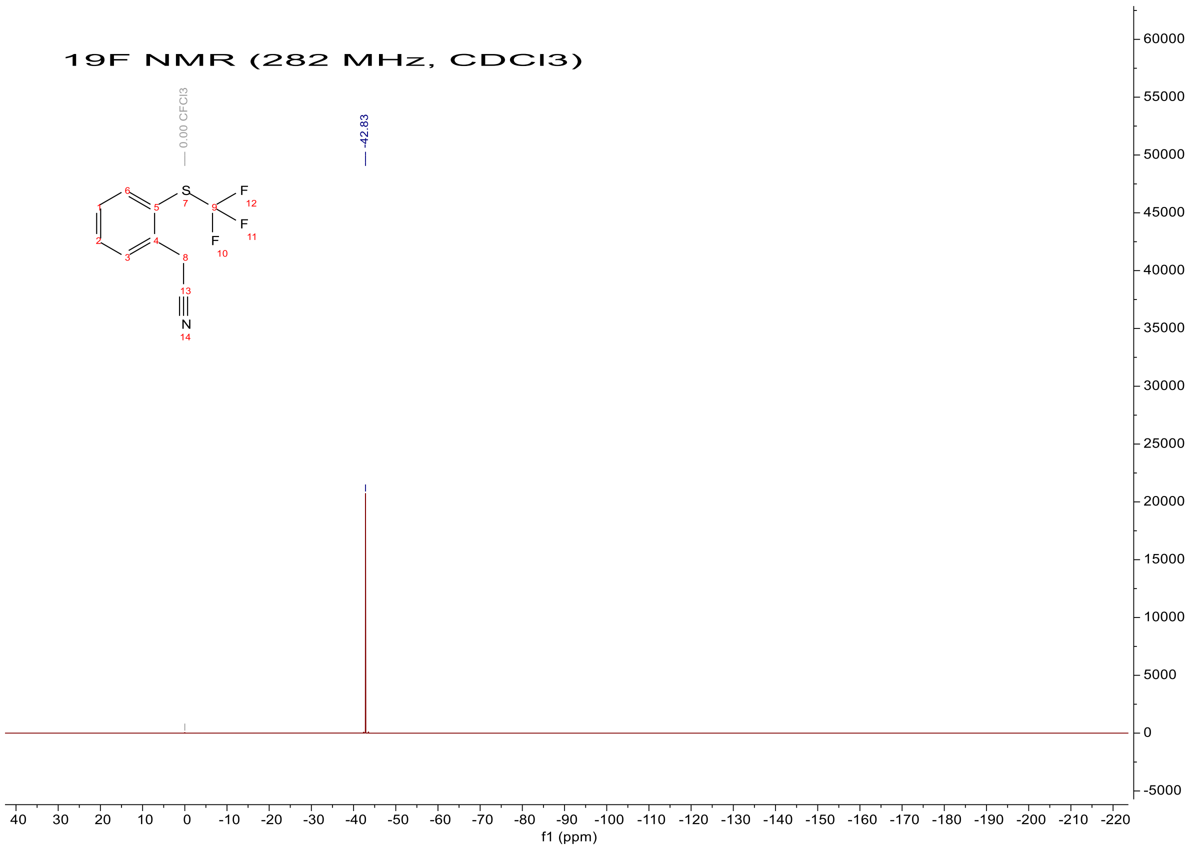

19F NMR (282 MHz, CDCl3)
0.00 CFCl3
-42.83
S
F
F
F
N
f1 (ppm)

2-(5-(Trifluoromethoxy)-2-((trifluoromethyl)thio)phenyl)acetonitrile (2b)

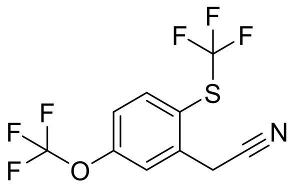

$C_{10}H_5F_6NOS$
301.21

Reaction carried out following the general procedure 2 using 1-(trifluoromethoxy)-4-((trifluoromethyl)sulfinyl)benzene (138 mg, 0.49 mmol). NMR ^{19}F yield = 70%. Purified by preparative TLC (petroleum ether/diethyl ether 92:8), affording the product as a yellow oil (100 mg, 0.33 mmol). Yield = 67%.

* **HRMS ASAP+TOF**: Calculated for $C_{20}H_{11}F_{12}N_2O_2S_2$[2M+H]$^+$:603.0070 ; Found [2M+H]$^+$: 603.0069.

* **^{1}H NMR (300 MHz, CDCl$_3$) δ (ppm):** 7.82 (d, *J* = 9 Hz, 1H), 7.52 (d, *J* = 2 Hz, 1H), 7.29 (dd, *J* = 9, 2 Hz, 1H), 4.07 (s, 2H).

* **^{13}C NMR (75 MHz, CDCl$_3$) *δ* (ppm):** 152.2 (q, *J* = 2 Hz), 141.0, 138.8, 129.0 (q, *J* = 309 Hz), 122.1 (d, *J* = 8 Hz), 121.6 (q, *J* = 2 Hz), 121.3, 120.3 (q, *J* = 258 Hz) 116.5, 23.2

* **^{19}F NMR (282 MHz, CDCl$_3$) δ (ppm):** -42.83 (s), -58.88 (s)

1H NMR (300 MHz, CDCl3)

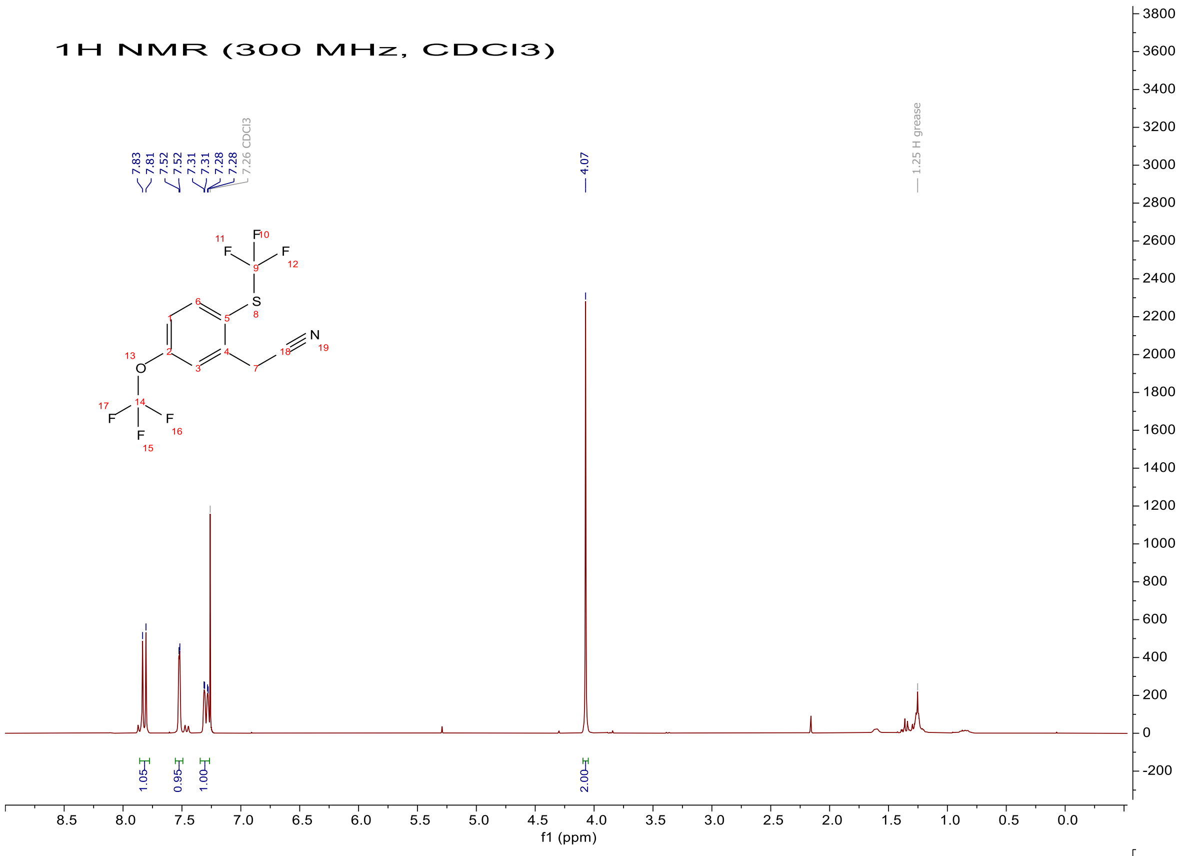

13C NMR (75 MHz, CDCl3)

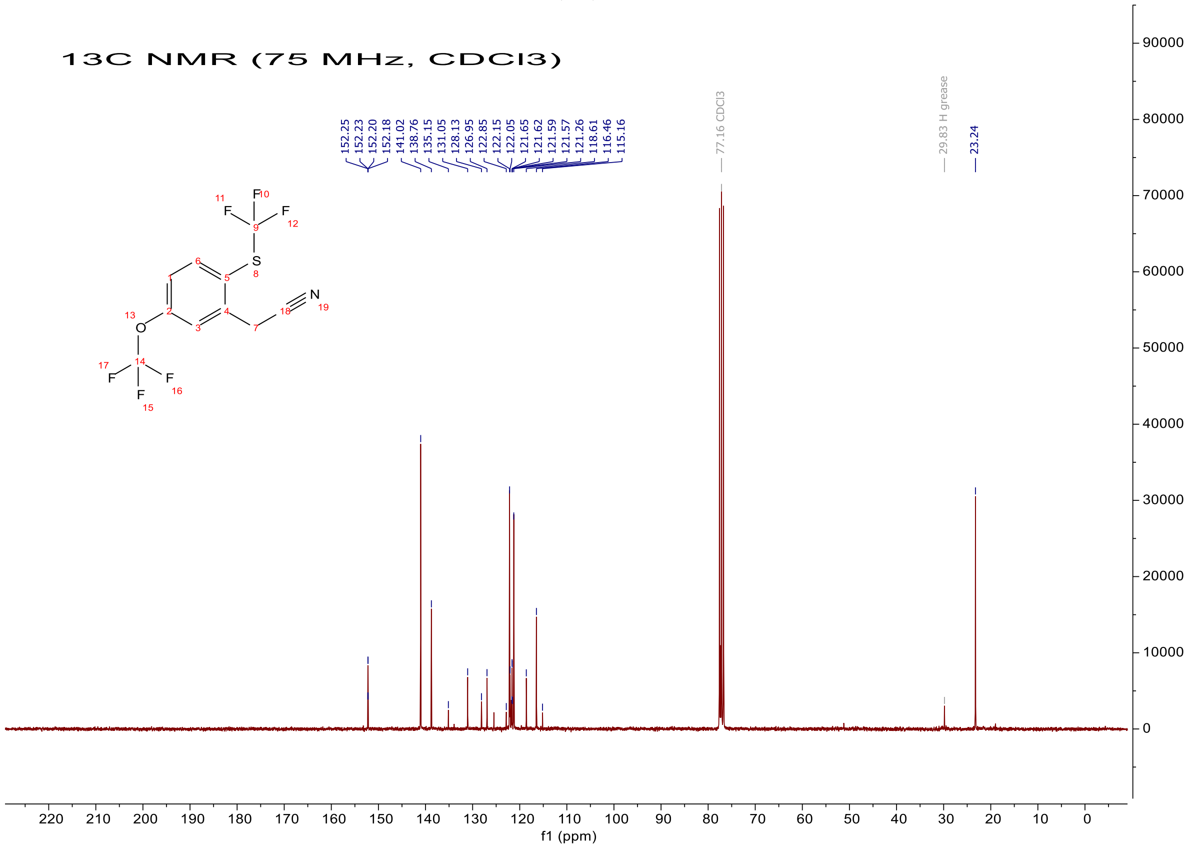

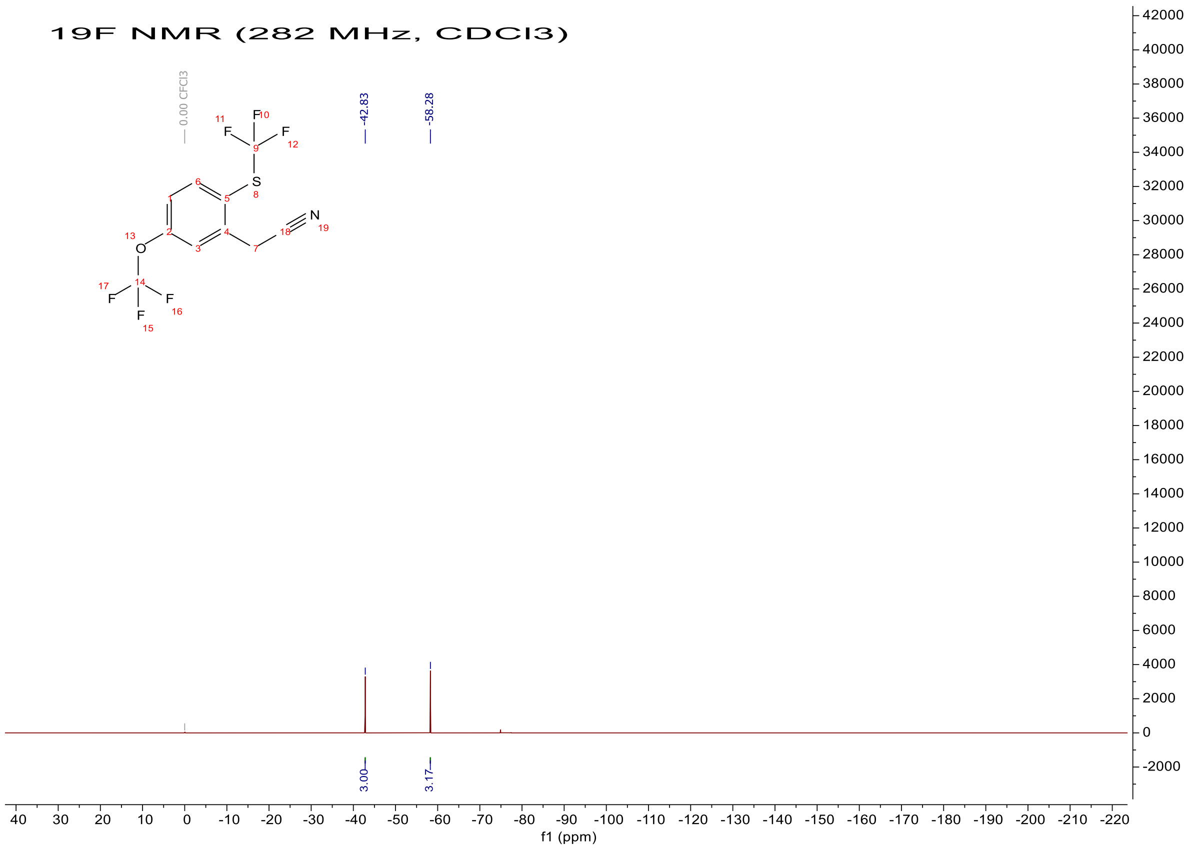
19F NMR (282 MHz, CDCl3)
0.00 CFCl3
-42.83
-58.28
3.00
3.17
f1 (ppm)

2-(5-(Chloromethyl)-2-((trifluoromethyl)thio)phenyl)acetonitrile (2c)

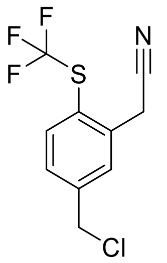

$C_{10}H_7ClF_3NS$
265.68

Reaction carried out following the general procedure 2 using 1-(chloromethyl)-4-((trifluoromethyl)sulfinyl)benzene(121 mg, 0.5 mmol) with acetonitrile. NMR ^{19}F yield = 85%. Purified by preparative TLC (petroleum ether/diethyl ether 7:3) affording the product as an orange oil (98 mg, 0.37 mmol). Yield = 74%.

* **HRMS ASAP+TOF**: Calculated for $C_{10}H_{15}ClF_3NS$ $[2M+H]^+$: 530.9958; Found $[M+H]^+$: 530.9963.

* **^{1}H NMR (300 MHz, CDCl$_3$) δ (ppm):** 7.77 (d, *J* = 8 Hz, 1H), 7.69 (dd, *J* = 2 Hz, 1 Hz, 1H), 7.49 – 7.45 (m, 1H), 4.62 (s, 2H), 4.07 (s, 2H).

* **^{13}C NMR (75 MHz, CDCl$_3$) *δ* (ppm):** 142.4, 139.7, 136.7, 130.0, 129.6, 129.2 (q, *J* = 307 Hz), 123.4 (q, *J* = 2 Hz), 117.0, 44.7, 23.1.

* **^{19}F NMR (282 MHz, CDCl$_3$) δ (ppm):** -42.66 (s)

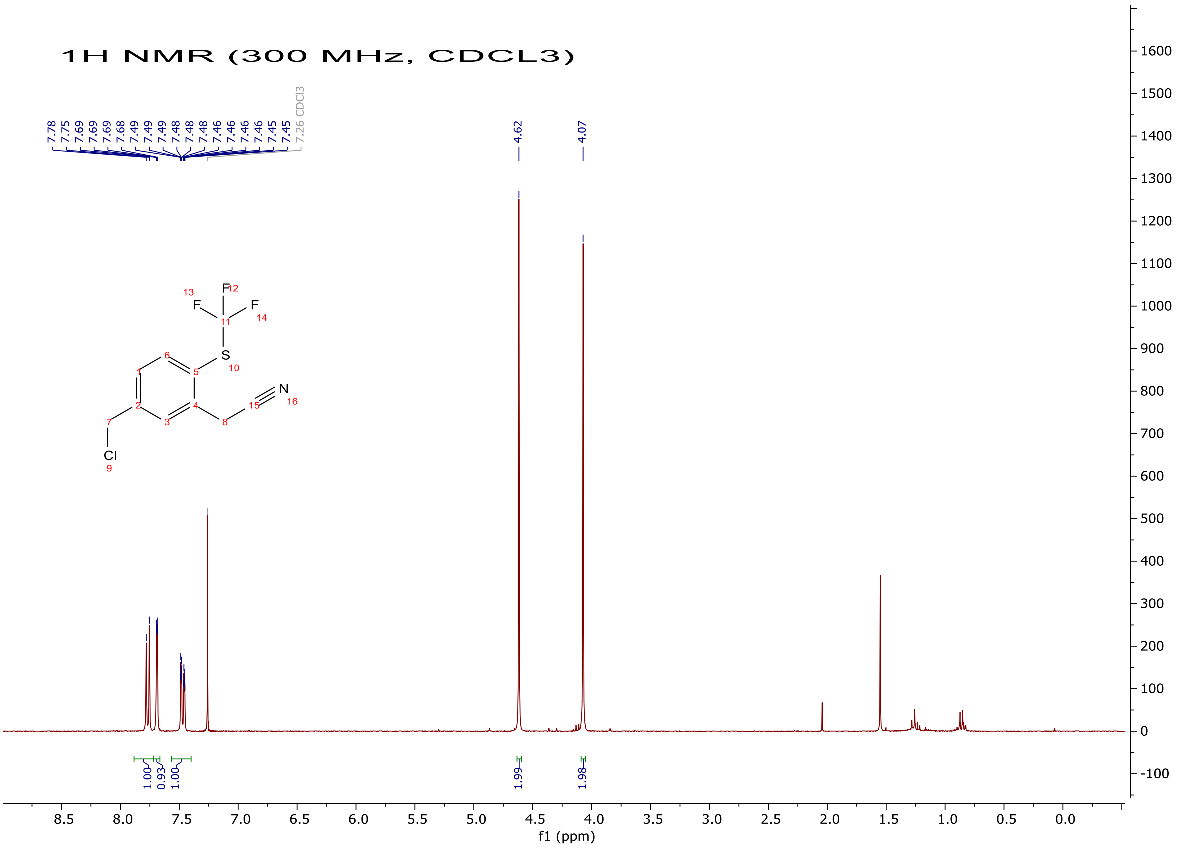

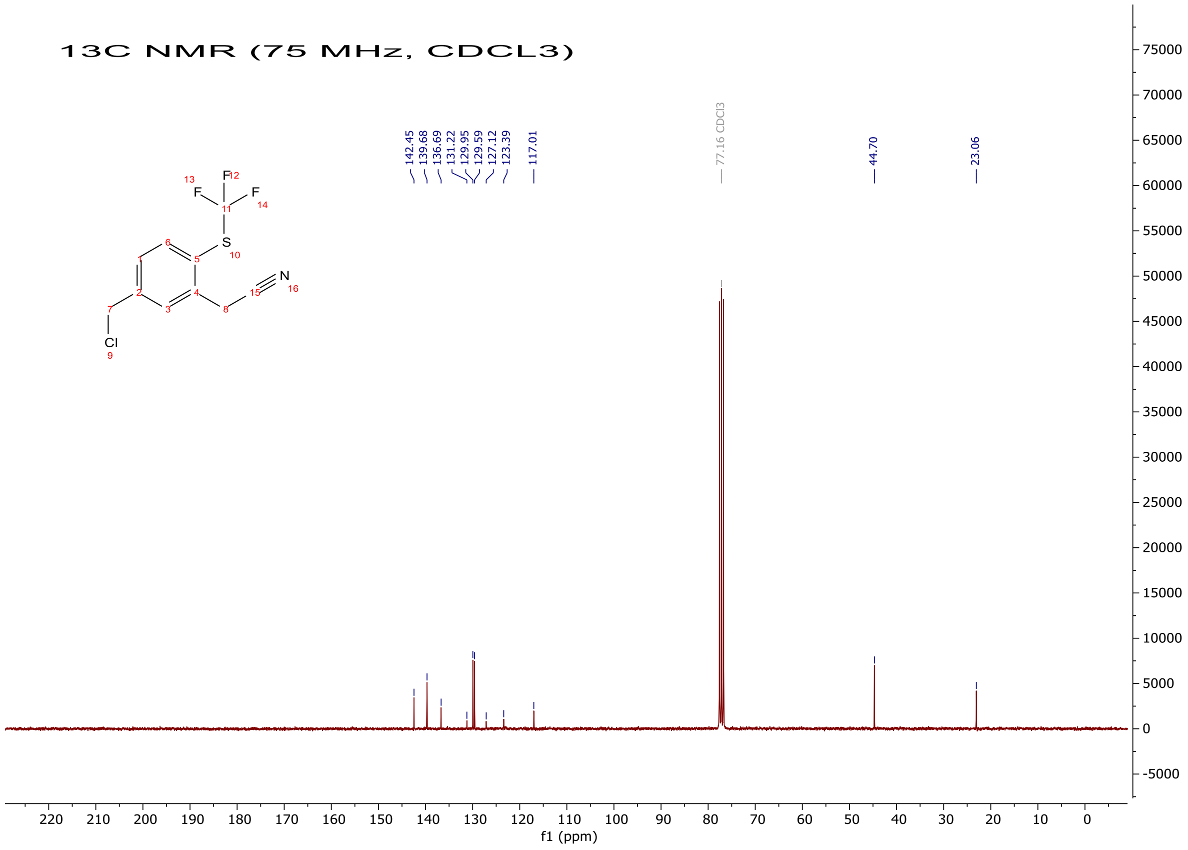
13C NMR (75 MHz, CDCL3)
142.45
139.68
136.69
131.22
129.95
129.59
127.12
123.39
117.01
77.16 CDCl3
44.70
23.06
f1 (ppm)

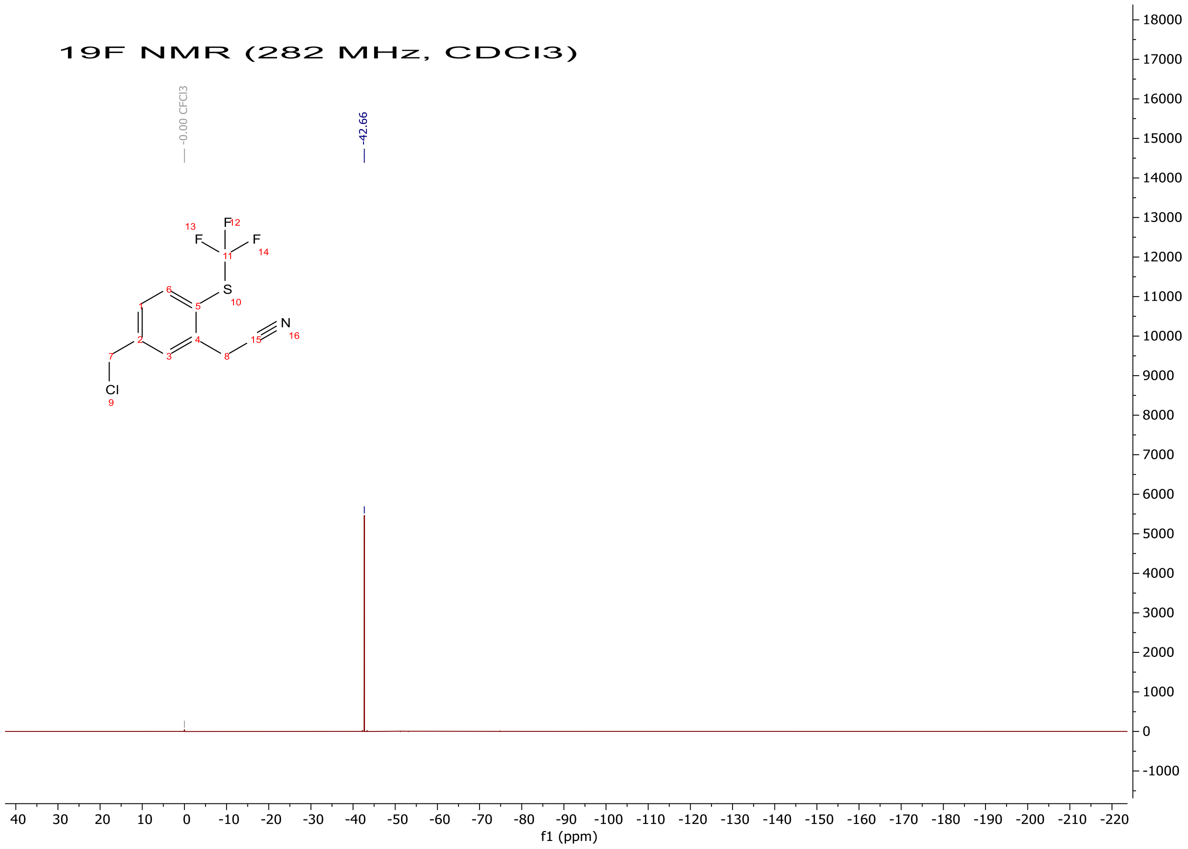
19F NMR (282 MHz, CDCl3)
-0.00 CFCl3
-42.66
f1 (ppm)

2-(5-Bromo-2-((trifluoromethyl)thio)phenyl)acetonitrile (2d)

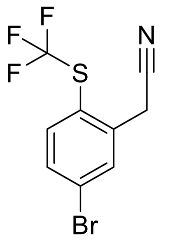

$C_9H_5BrF_3NS$
296.11

Reaction carried out following the general procedure 1 using 1-bromo-4-((trifluoromethyl)sulfinyl)benzene (137 mg, 0.5 mmol) and acetonitrile. NMR ^{19}F yield = 73%. Purified by preparative TLC (*n*-pentane/EtOAc 84:16) affording the product as an orange oil (87 mg, 0.3 mmol). Yield = 59%.

* **^{1}H NMR (300 MHz, CDCl₃) δ (ppm):** 7.83 (d, *J* = 1 Hz, 1H), 7.67 – 7.55 (m, 2H), 4.04 (s, 2H).

* **^{13}C NMR (75 MHz, CDCl₃) δ (ppm):** 140.4, 138.0, 133.2, 133.0, 128.9 (q, *J* = 309 Hz), 122.4 (q, *J* =2 Hz), 127.6, 116.6, 22.9

* **^{19}F NMR (282 MHz, CDCl₃) δ (ppm):** -42.77 (s)

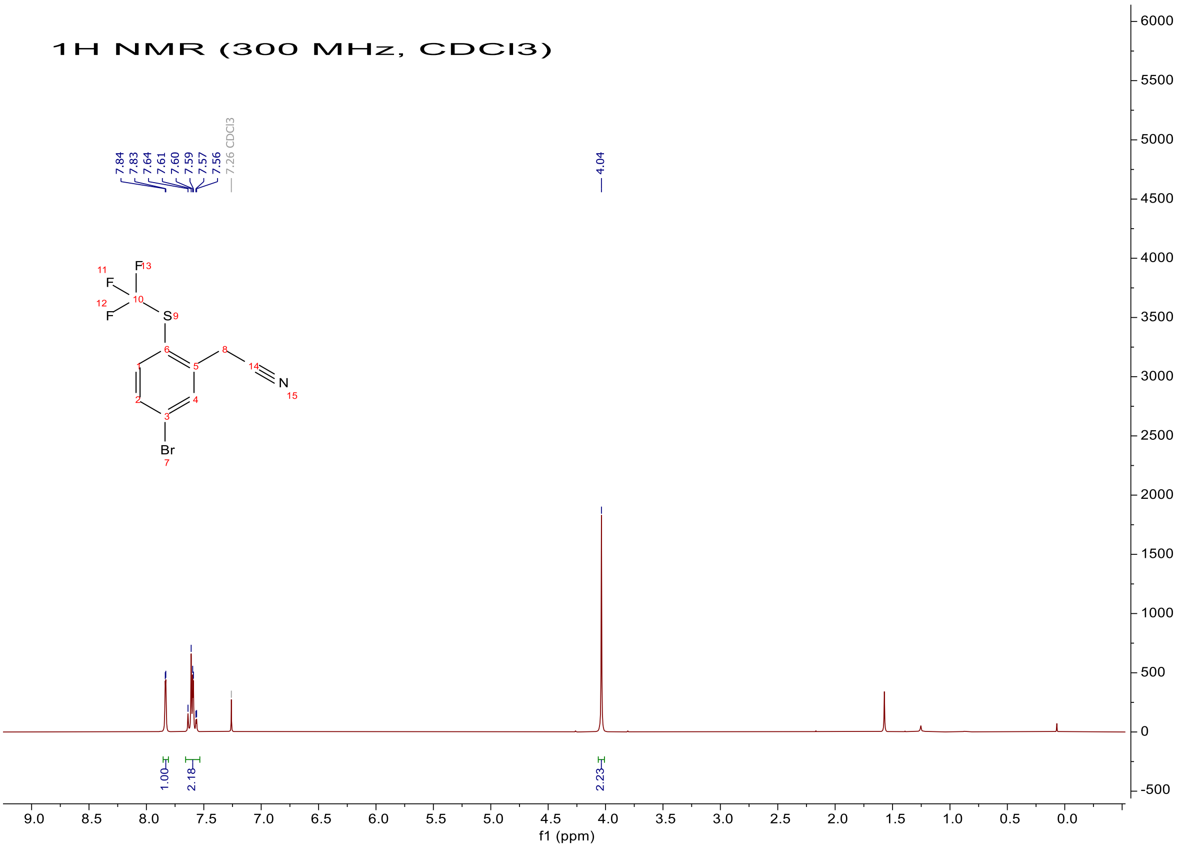

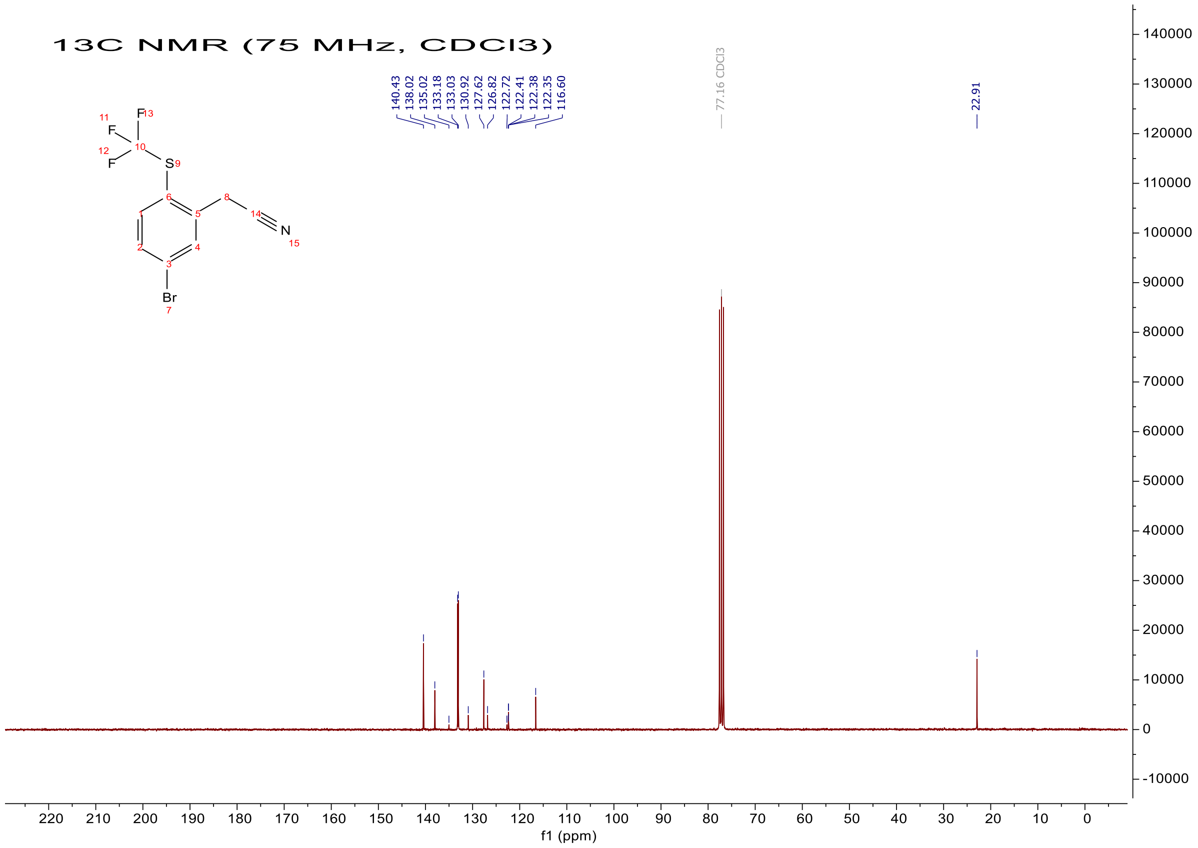
13C NMR (75 MHz, CDCl3)
140.43
138.02
135.02
133.18
133.03
130.92
127.62
126.82
122.72
122.41
122.38
122.35
116.60
77.16 CDCl3
22.91
F
F
F
S
N
Br
f1 (ppm)

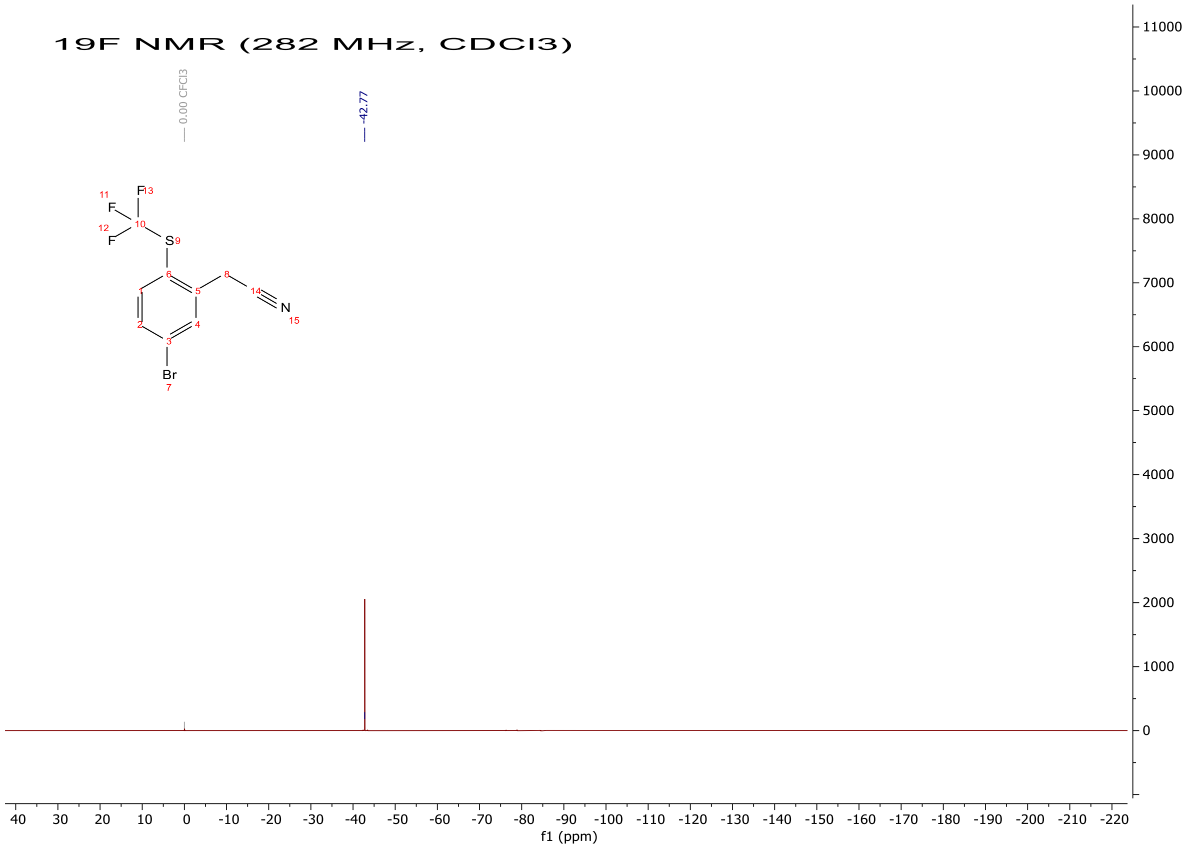
19F NMR (282 MHz, CDCl3)
0.00 CFCl3
-42.77
F
F
F
S
N
Br
f1 (ppm)

2-(3-Fluoro-5-methyl-2-((trifluoromethyl)thio)phenyl)acetonitrile (2e)

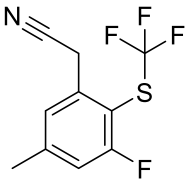

$C_{10}H_7F_4NS$
249.23

Reaction carried out following the general procedure 2 using 2-fluoro-4-methyl-1-((trifluoromethyl)sulfinyl)benzene (110 mg, 0.5 mmol) with acetonitrile. NMR ^{19}F yield = 37%. Purified by flash chromatography (Gradient: petroleum ether/diethyl ether 100:0 to 80:20), affording the product as an orange oil (42 mg, 0.17 mmol). Yield = 35%.

* **HRMS ASAP+TOF**: Calculated for $C_{10}H_8F_4NS[M+H]^+$: 250.0314; Found $[M+H]^+$: 250.0322

* **^1H NMR (300 MHz, $CDCl_3$) δ (ppm):** 7.31 (s, 1H), 7.05 (d, *J* = 9 Hz, 1H), 4.04 (s, 2H), 2.44 (s, 3H).

* **^{13}C NMR (75 MHz, $CDCl_3$) *δ* (ppm):** 164.3 (d, *J* = 253 Hz), 146.3 (d, *J* = 9 Hz), 137.9, 128.9 (qd, *J* = 311, 2 Hz), 126.3 (d, *J* = 3 Hz), 117.3 (d, *J* = 23 Hz), 117.0, 107.6 (qd, *J* = 20, 2 Hz), 22.7 (d, *J* = 4 Hz), 21.7 (d, *J* = 2 Hz).

* **^{19}F NMR (282 MHz, $CDCl_3$) δ (ppm):** -42.76 (d, *J* = 6 Hz), -101.52 (q, *J* = 6 Hz).

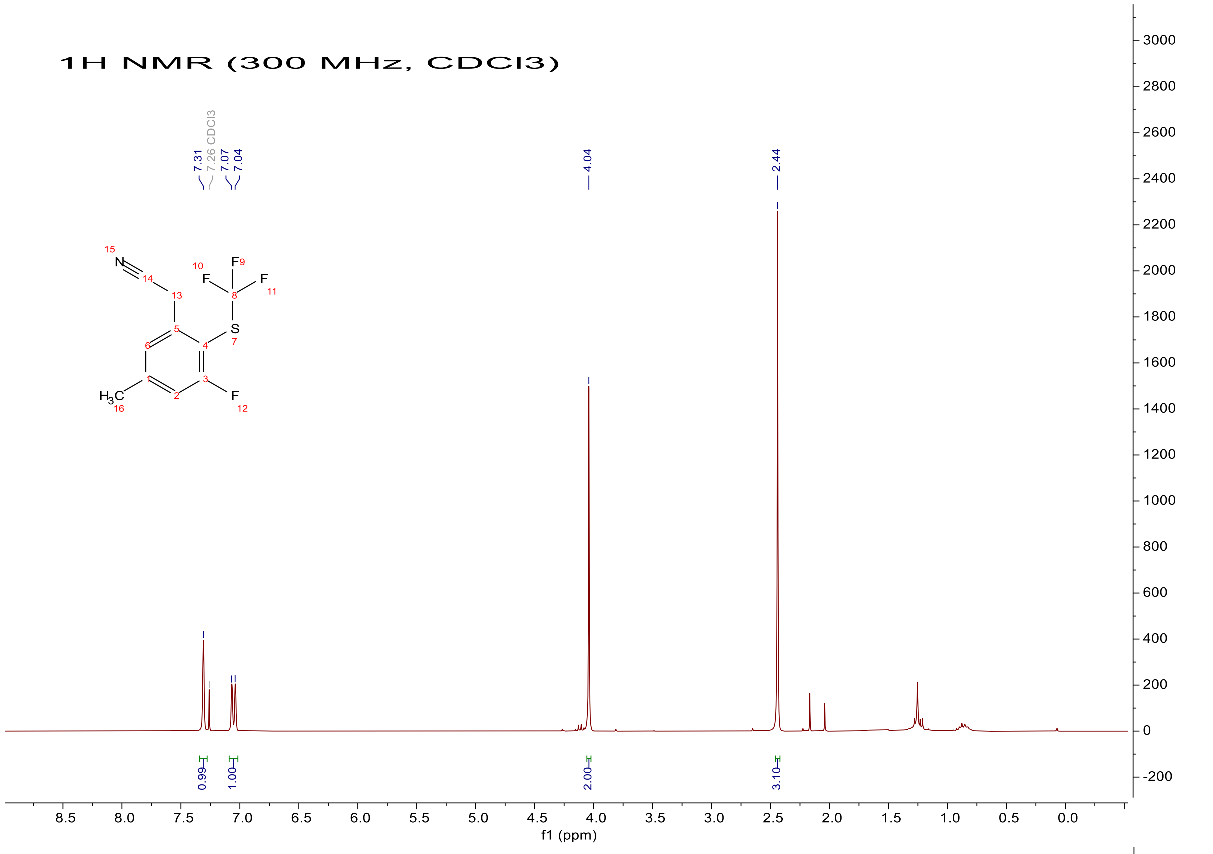
1H NMR (300 MHz, CDCl3)
7.31
7.26 CDCl3
7.07
7.04
4.04
2.44
0.99
1.00
2.00
3.10
f1 (ppm)

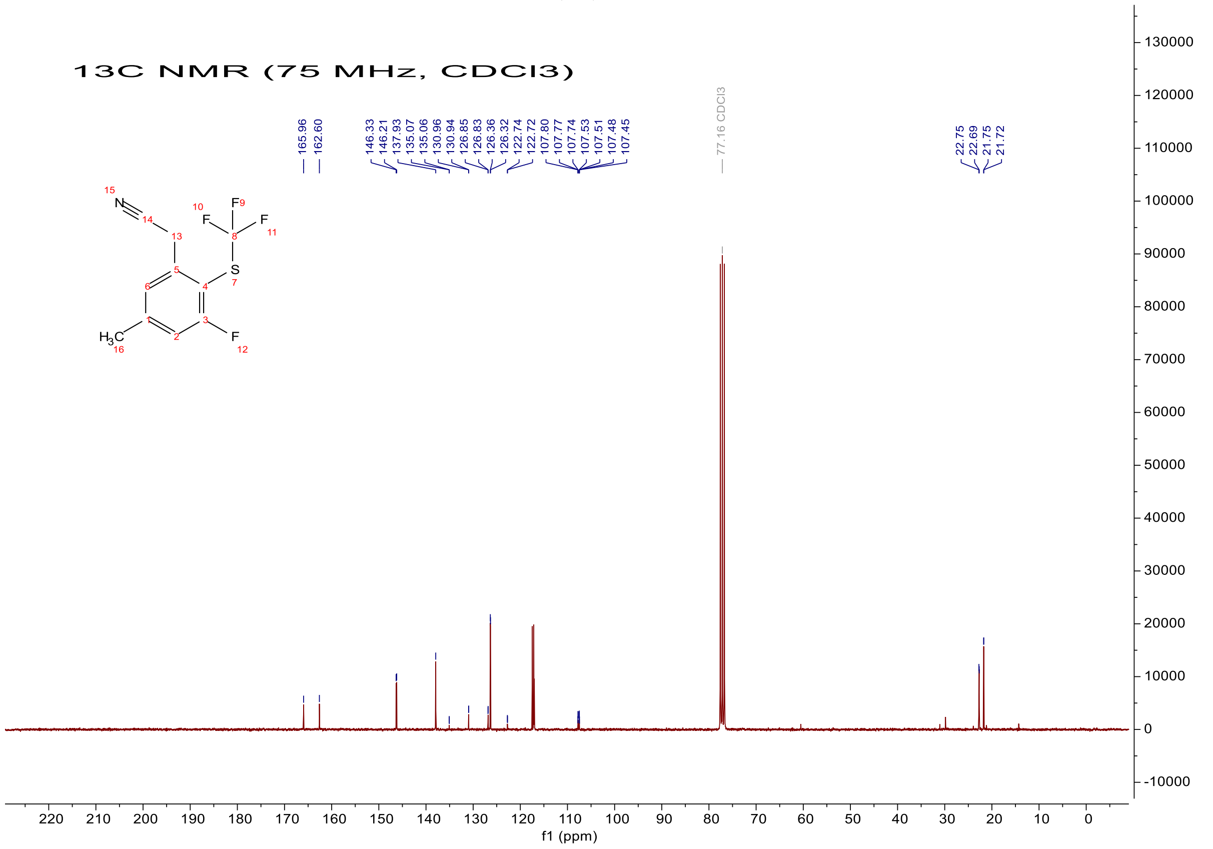
13C NMR (75 MHz, CDCl3)
165.96
162.60
146.33
146.21
137.93
135.07
135.06
130.96
130.94
126.85
126.83
126.36
126.32
122.74
122.72
107.80
107.77
107.74
107.53
107.51
107.48
107.45
77.16 CDCl3
22.75
22.69
21.75
21.72
f1 (ppm)

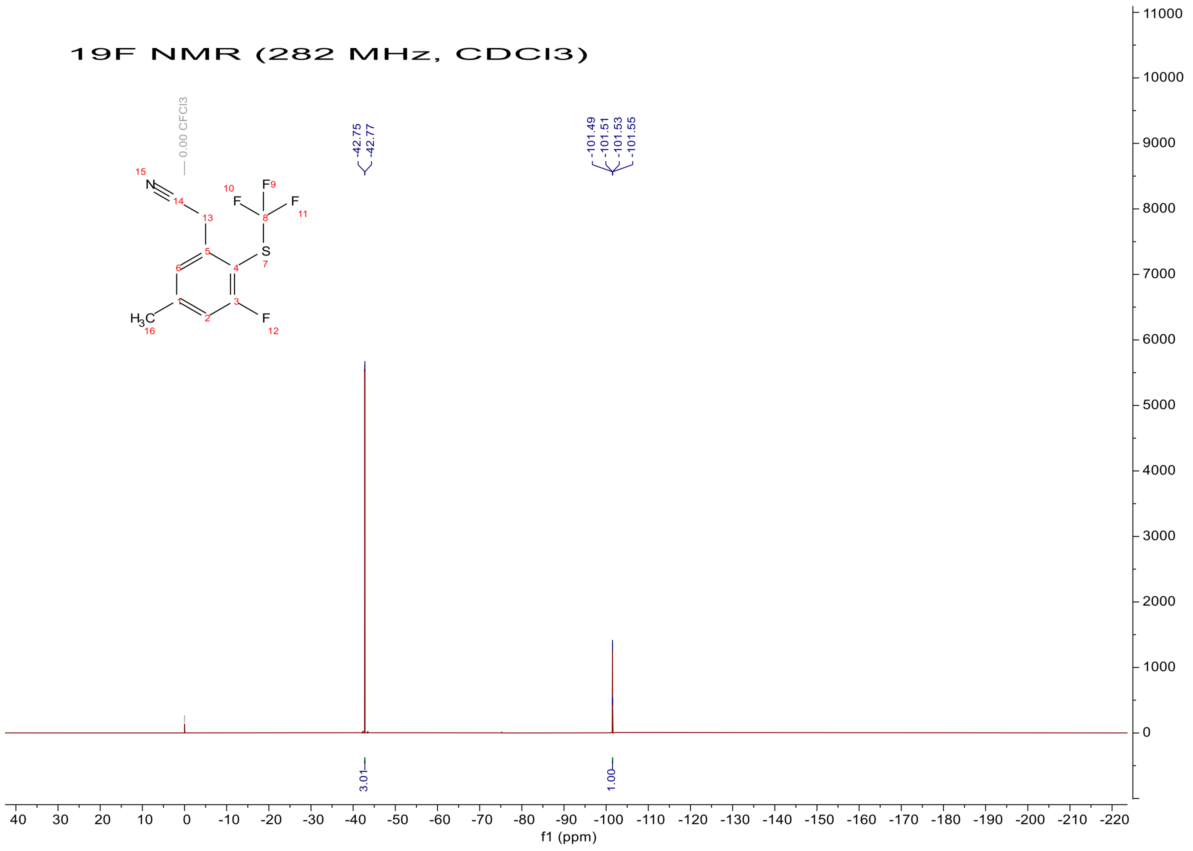

19F NMR (282 MHz, CDCl3)
0.00 CFCl3
-42.75
-42.77
-101.49
-101.51
-101.53
-101.55
3.01
1.00
f1 (ppm)

2-(4-Methyl-2-((trifluoromethyl)thio)phenyl)acetonitrile (2f)

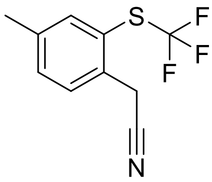

$C_{10}H_8F_3NS$
231.24

Reaction carried out following the general procedure 2 using 1-methyl-3-((trifluoromethyl)sulfinyl)benzene (58 mg, 0.28 mmol). NMR ^{19}F yield = 23%. Purified by preparative TLC (petroleum ether/diethyl ether 8:2), affording the product as a yellow oil (12 mg, 0.05 mmol). Yield = 18%.

* **HRMS ASAP+TOF**: Calculated for $C_{10}H_9F_3NS[M+H]^+$:232.0408 ; Found $[M+H]^+$: 232.0408

* **^1H NMR (300 MHz, $CDCl_3$) δ (ppm):** 7.57 (s, 1H), 7.54 (d, *J* = 8 Hz, 1H), 7.37 (dd, *J* = 8 Hz, 2 Hz, 1H), 4.02 (s, 2H), 2.40 (s, 3H).

* **^{13}C NMR (75 MHz, $CDCl_3$) *δ* (ppm):** 139.9, 139.8, 133.4, 133.1, 129.8, 117.5, 129.4 (q, *J* = 309 Hz), 123.0 (q, *J* = 2 Hz), 22.6, 20.9

* **^{19}F NMR (282 MHz, $CDCl_3$) δ (ppm):** - 42.86 (s)

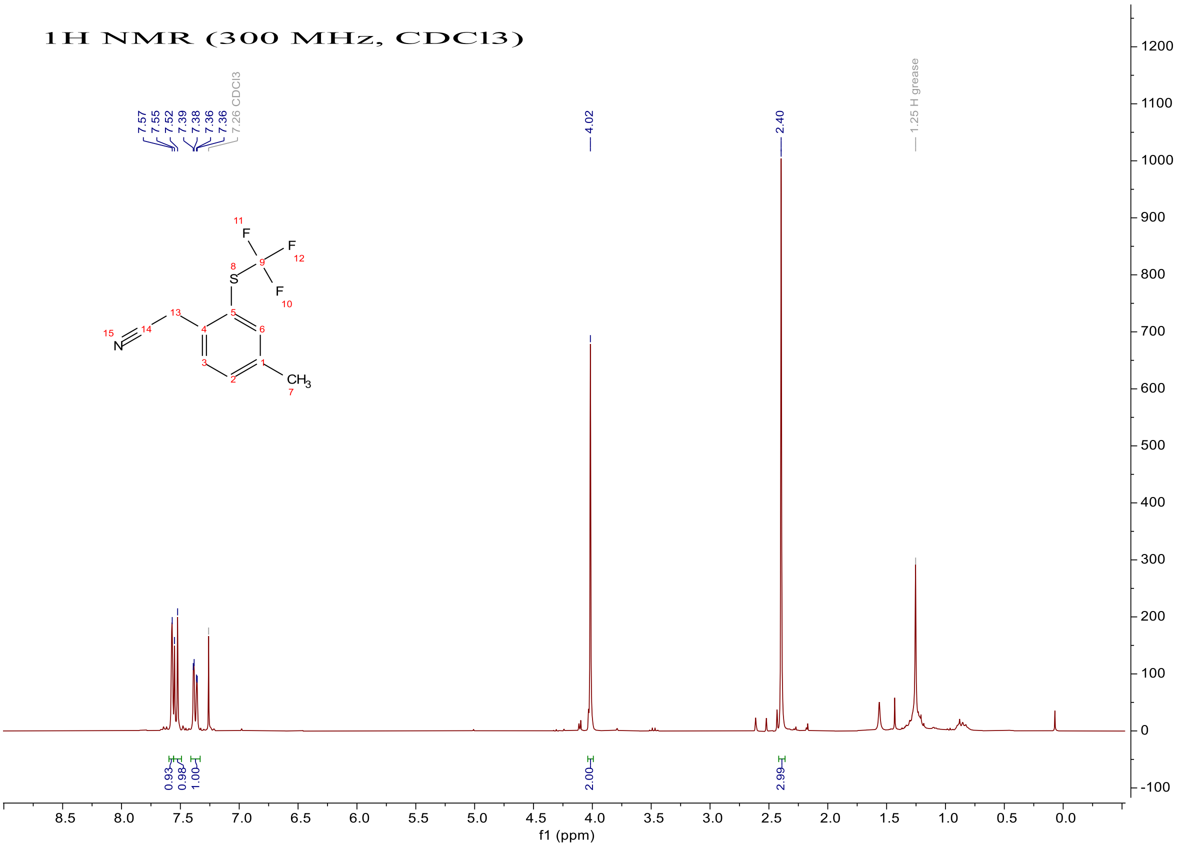

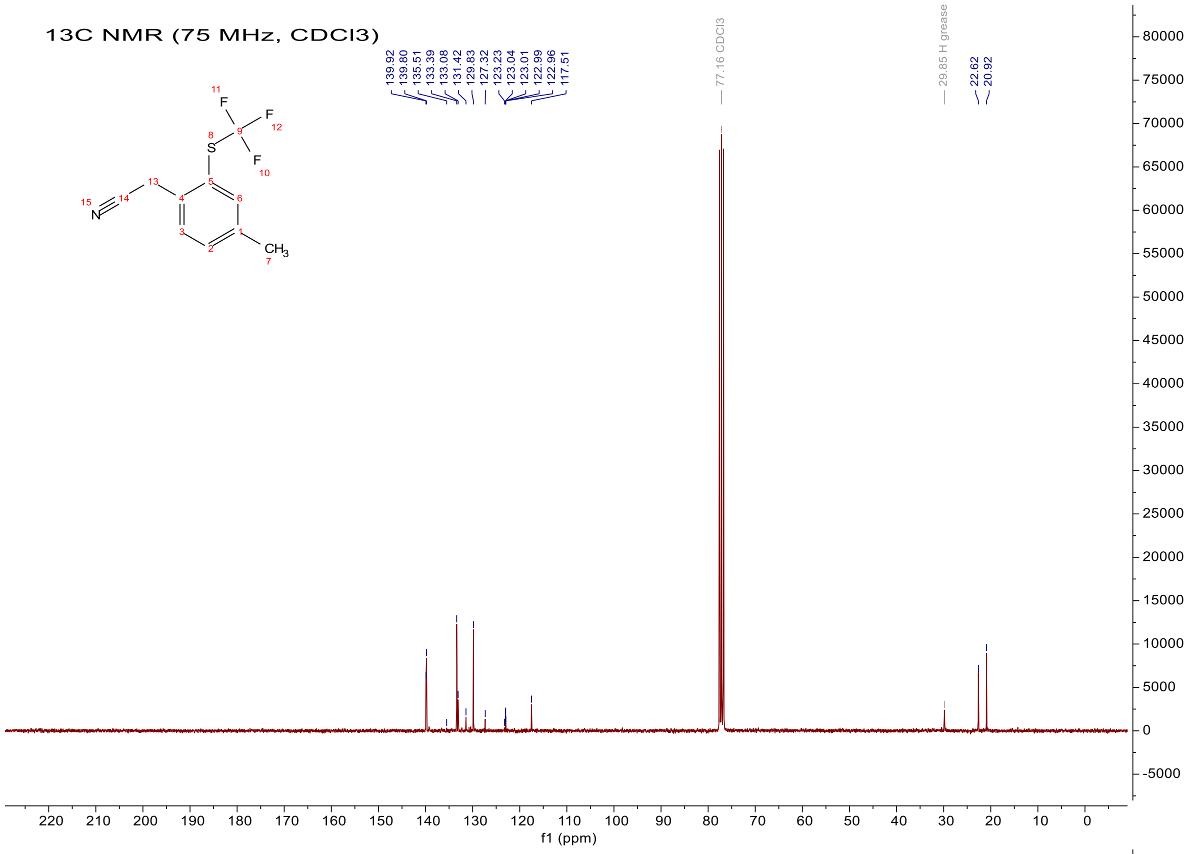
13C NMR (75 MHz, CDCl3)
139.92
139.80
135.51
133.39
133.08
131.42
129.83
127.32
123.23
123.04
123.01
122.99
122.96
117.51
77.16 CDCl3
29.85 H grease
22.62
20.92
f1 (ppm)

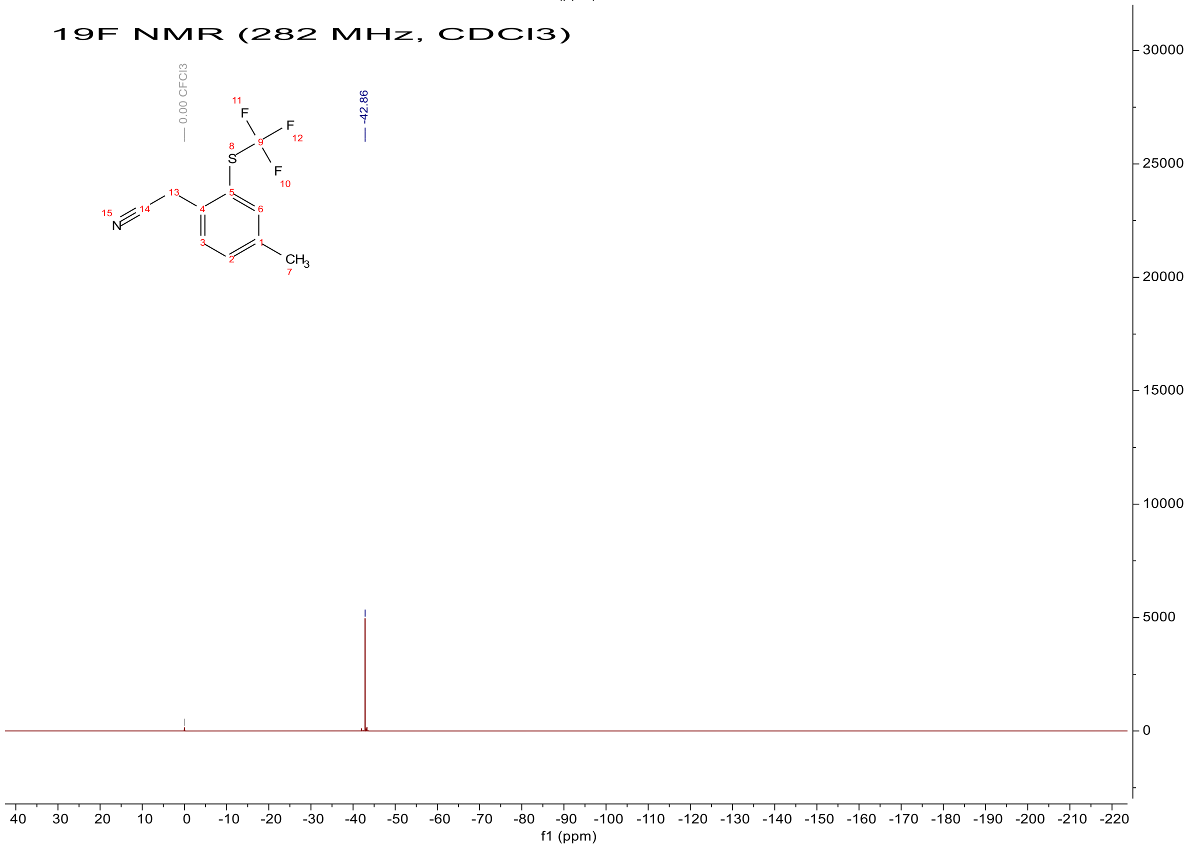
19F NMR (282 MHz, CDCl3)
0.00 CFCl3
-42.86
f1 (ppm)

2-(2-Methyl-6-((trifluoromethyl)thio)phenyl)acetonitrile (2f')

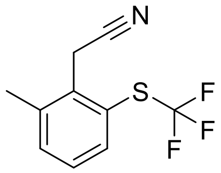

$C_{10}H_8F_3NS$
231.24

Reaction carried out following the general procedure 2 using 1-methyl-3-((trifluoromethyl)sulfinyl)benzene (58 mg, 0.28 mmol). NMR ^{19}F yield = 29%. Purified by preparative TLC (petroleum ether/diethyl ether 8:2), affording the product as a yellow oil (12 mg, 0.05 mmol). Yield = 19%.

* **HRMS ASAP+TOF**: Calculated for $C_{10}H_9F_3NS[M+H]^+$:232.0408 ; Found $[M+H]^+$: 232.0408.

* **^1H NMR (300 MHz, $CDCl_3$) δ (ppm):** 7.65 (d, *J* = 8 Hz, 1H), 7.41 (d, *J* = 8 Hz, 1H), 7.33 (t, *J* = 8 Hz, 1H), 4.10 (s, 2H), 2.52 (s, 3H).

* **^{13}C NMR (75 MHz, $CDCl_3$) δ (ppm):** 139.4, 137.2, 134.9, 134.6, 129.4, 129.3 (q, *J* = 309 Hz), 124.50 (q, *J* = 2 Hz), 116.6, 20.7, 20.1

* **^{19}F NMR (282 MHz, $CDCl_3$) δ (ppm):** - 43.13 (s)

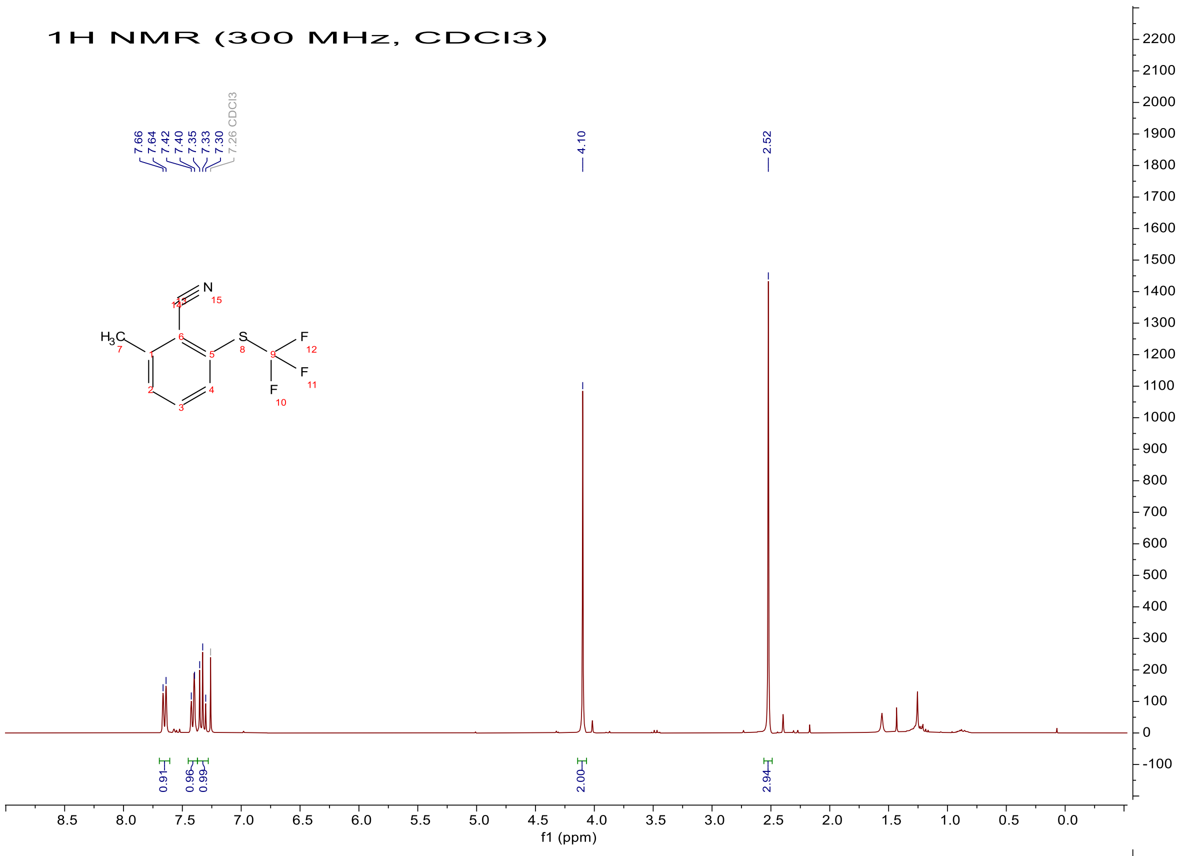
1H NMR (300 MHz, CDCl3)
7.66 7.64 7.42 7.40 7.35 7.33 7.30 7.26 CDCl3
4.10
2.52
0.91 0.96 0.99
2.00
2.94
f1 (ppm)

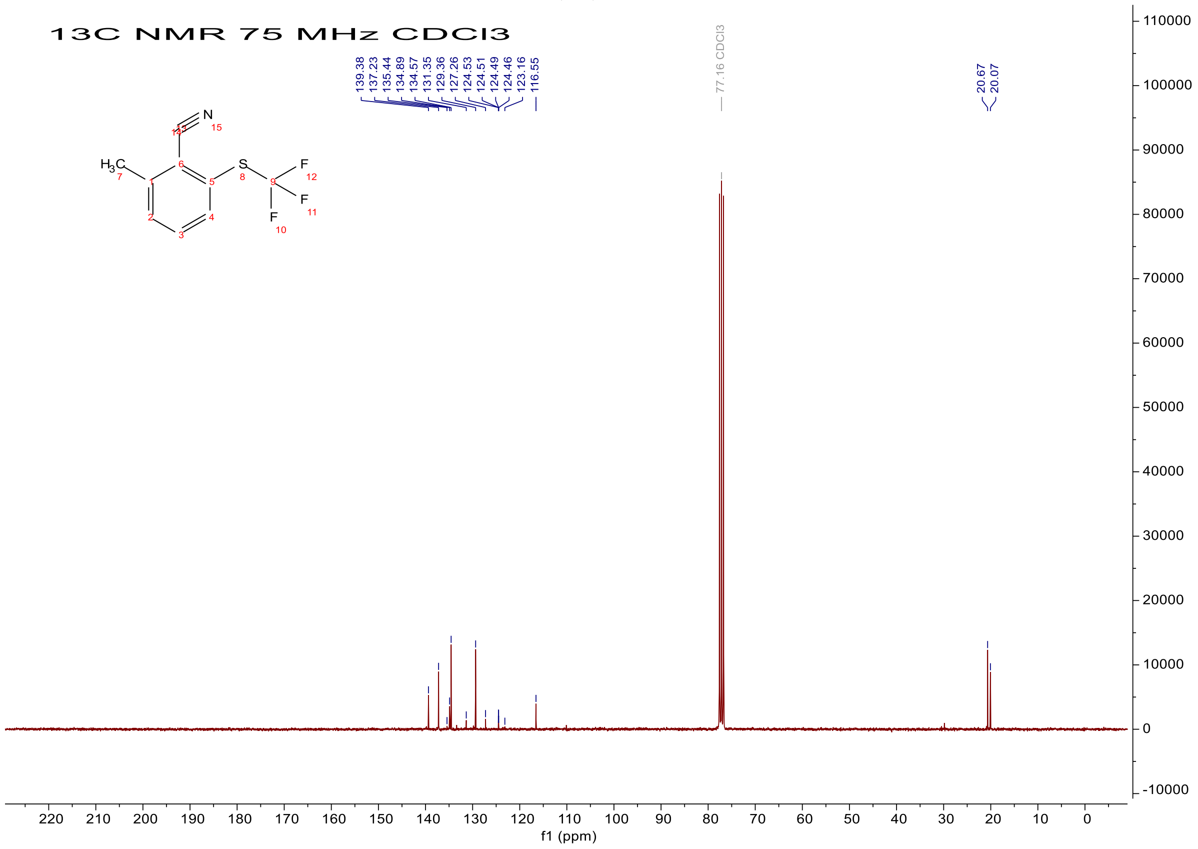
13C NMR 75 MHz CDCl3
139.38 137.23 135.44 134.89 134.57 131.35 129.36 127.26 124.53 124.51 124.49 124.46 123.16 116.55
77.16 CDCl3
20.67 20.07
f1 (ppm)

19F NMR (282 MHz, CDCl3)

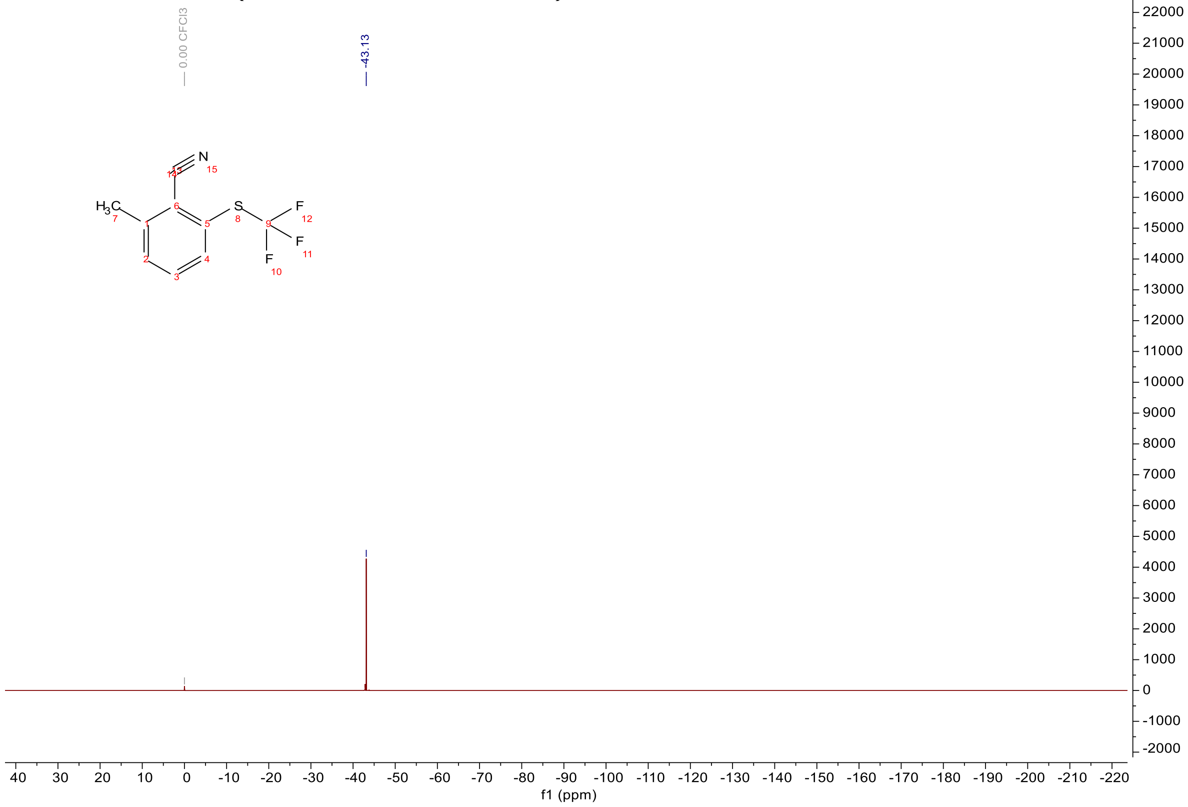

2,2'-(2-((Trifluoromethyl)thio)-1,3-phenylene)diacetonitrile (2g)

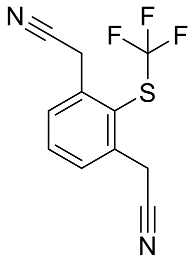

$C_{11}H_7F_3N_2S$
256.25

Reaction carried out following the general procedure 2 using 2-(2-((trifluoromethyl)sulfinyl)phenyl)acetonitrile (116 mg, 0.5 mmol) with acetonitrile. NMR ^{19}F yield = 62%. Purified by flash chromatography (Gradient: petroleum ether/diethyl ether 100:0 to 60:40), affording the product as an orange oil (63 mg, 0.25 mmol). Yield = 49%.

* **HRMS ASAP+TOF**: Calculated for $C_{11}H_8F_3N_2S[M+H]^+$: 257.0360; Found $[M+H]^+$: 257.0368

* **^{1}H NMR (300 MHz, CDCl$_3$) δ (ppm):** 7.76 – 7.62 (m, 3H), 4.13 (s, 4H).

* **^{13}C NMR (75 MHz, CDCl$_3$) δ (ppm):** 138.1, 135.2, 130.2 (2C), 129.0 (q, *J* = 311 Hz), 122.5 (q, *J* = 2 Hz, 2C), 117.0 (2C), 23.8 (q, *J* = 2 Hz, 2C)

* **^{19}F NMR (282 MHz, CDCl$_3$) δ (ppm):** -41.77 (s)

1H NMR (300 MHz, CDCl3)

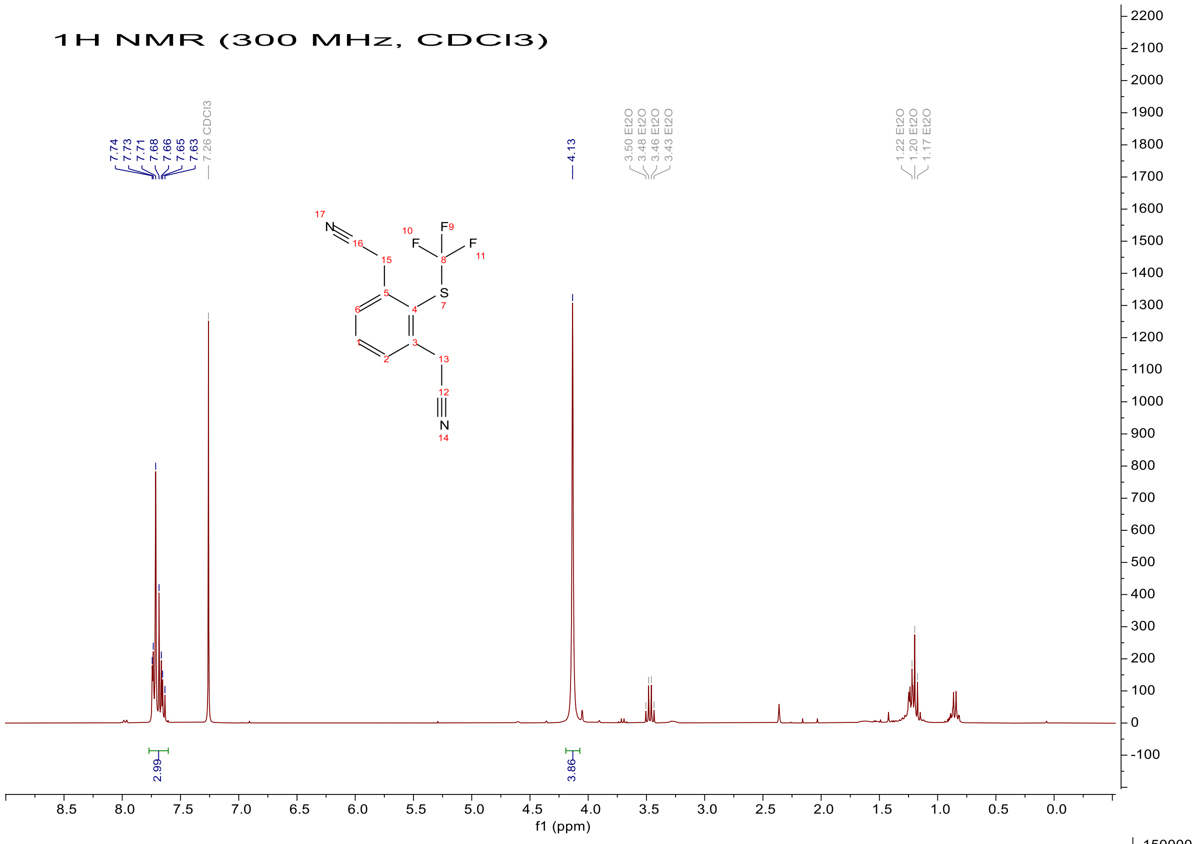

13C NMR (75 MHz, CDCl3)

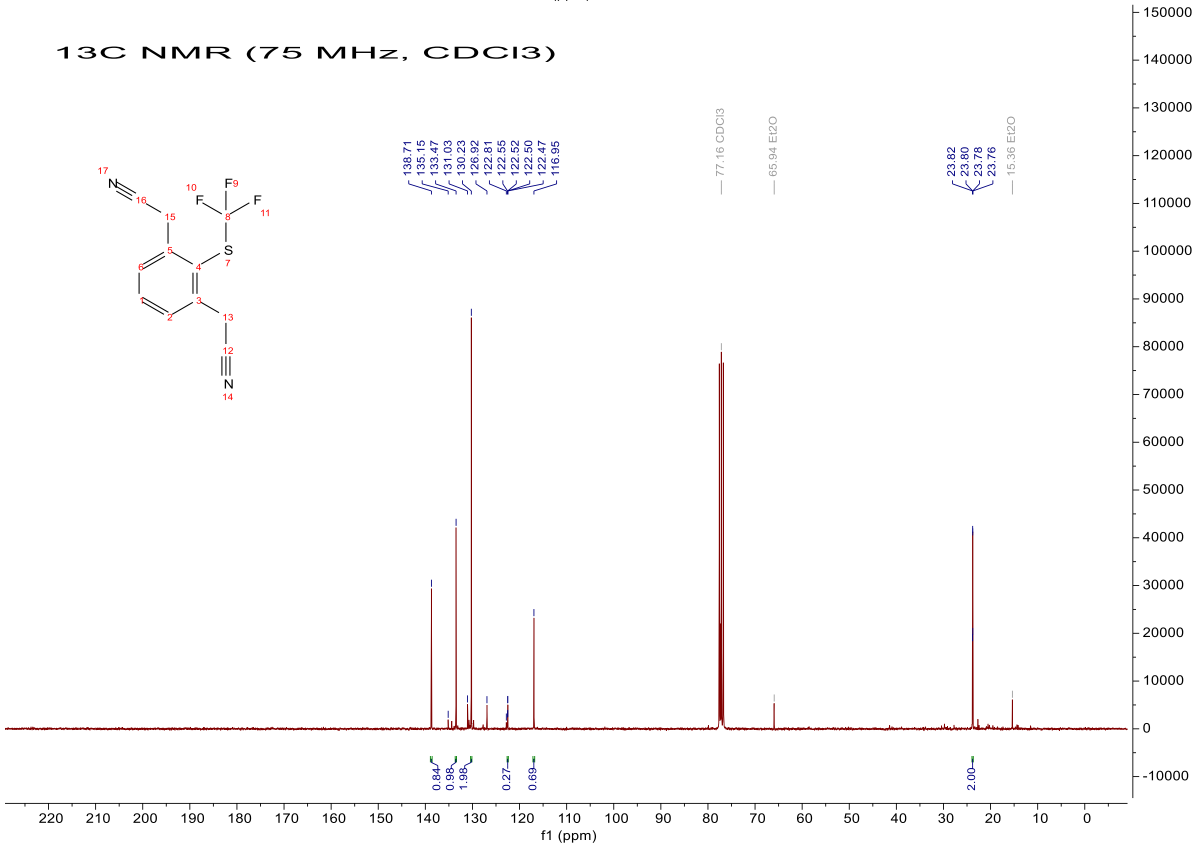

19F NMR (282 MHz, CDCl3)

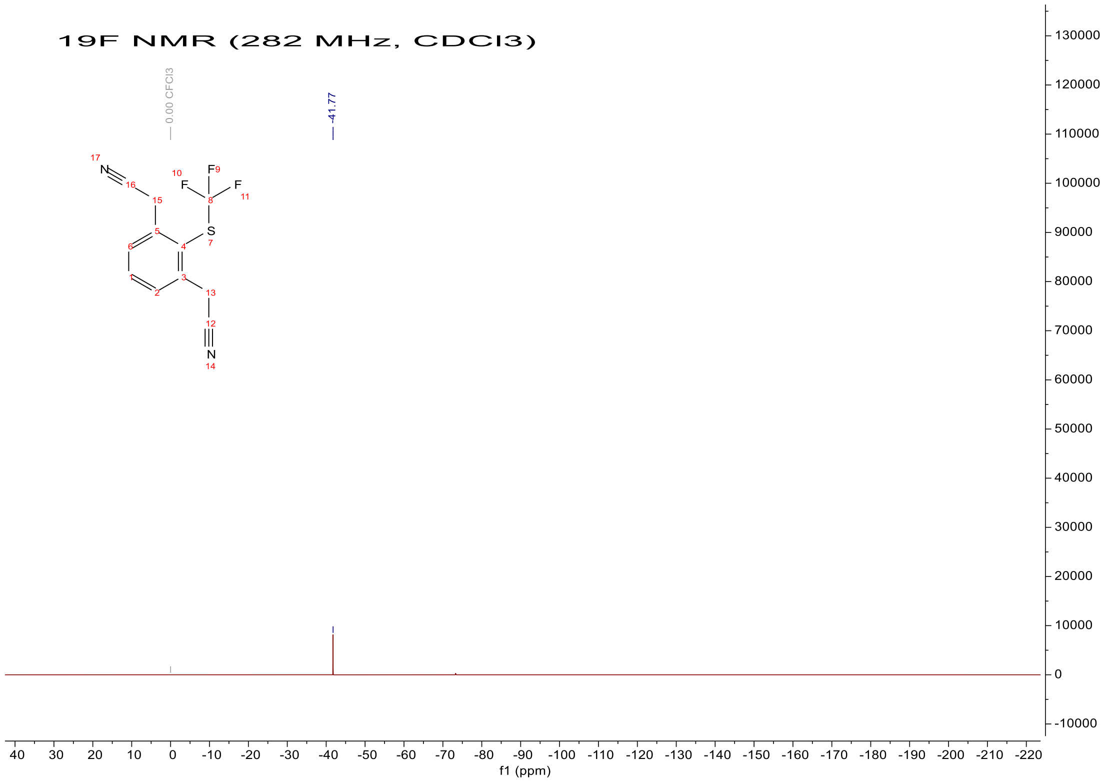

2-(5-Bromo-2-((difluoromethyl)thio)phenyl)acetonitrile (2h)

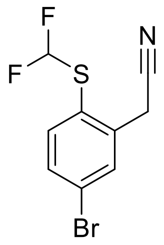

$C_9H_6BrF_2NS$
278.11

Reaction carried out following the general procedure 2 using 1-bromo-4-((difluoromethyl)sulfinyl)benzene (144 mg, 0.57 mmol). NMR ^{19}F yield = 51%. Purified by preparative TLC (petroleum ether/diethyl ether 7:3), affording the product as a yellow oil (71 mg, 0.26 mmol). Yield = 45%.

* **HRMS ASAP+TOF**: Calculated for $C_9H_7BrF_2NS[M+H]^+$: ; Found $[M+H]^+$: 276.9361

* **^1H NMR (300 MHz, $CDCl_3$) δ (ppm):** 7.79 – 7.78 (m, 1H), 7.56 – 7.54 (m, 2H), 6.79 (t, *J* = 56 Hz, 1H), 4.00 (d, *J* = 1 Hz, 2H).

* **^{13}C NMR (75 MHz, $CDCl_3$) *δ* (ppm):** 140.0, 137.8, 132.9, 132.7, 126.4, 123.4 (t, *J* = 3 Hz), 119.6 (t, *J* = 278 Hz), 115.9, 23.1

* **^{19}F NMR (282 MHz, $CDCl_3$) δ (ppm):** - 91.97 (d, *J* = 56 Hz)

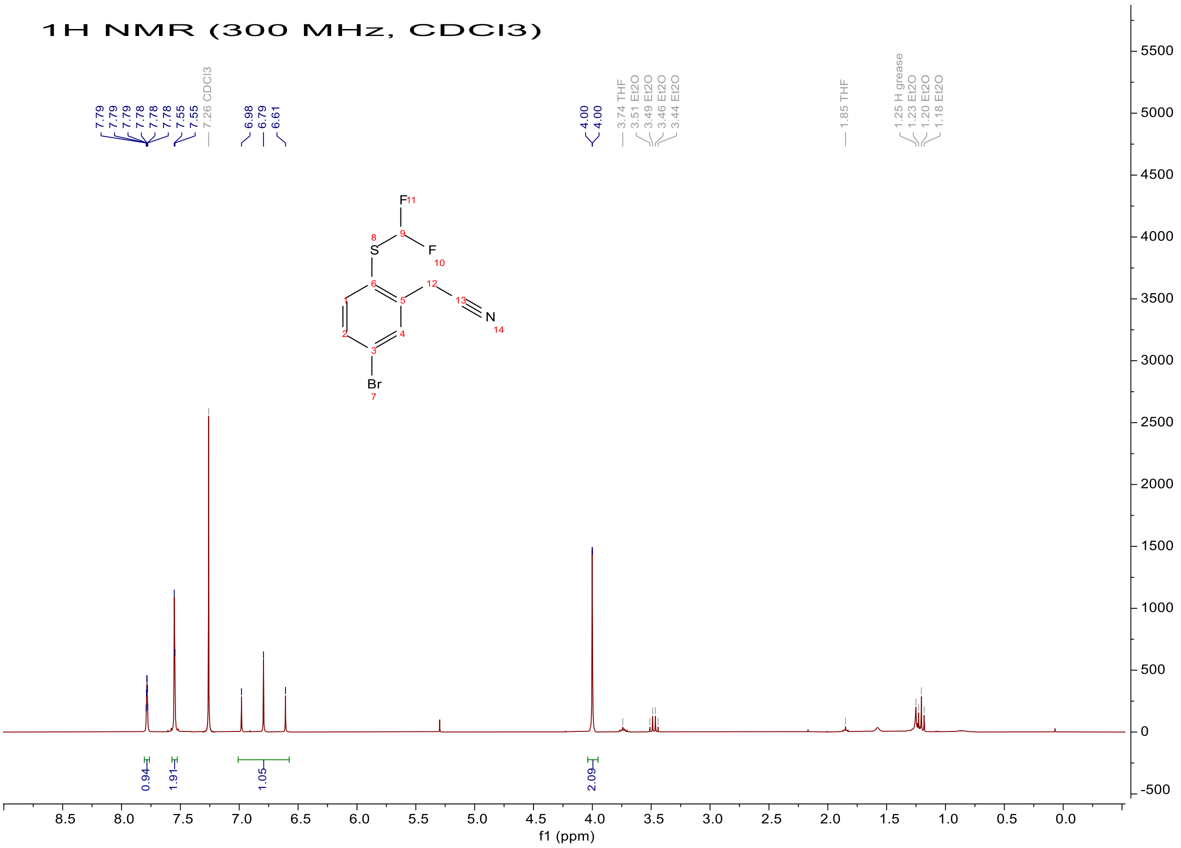

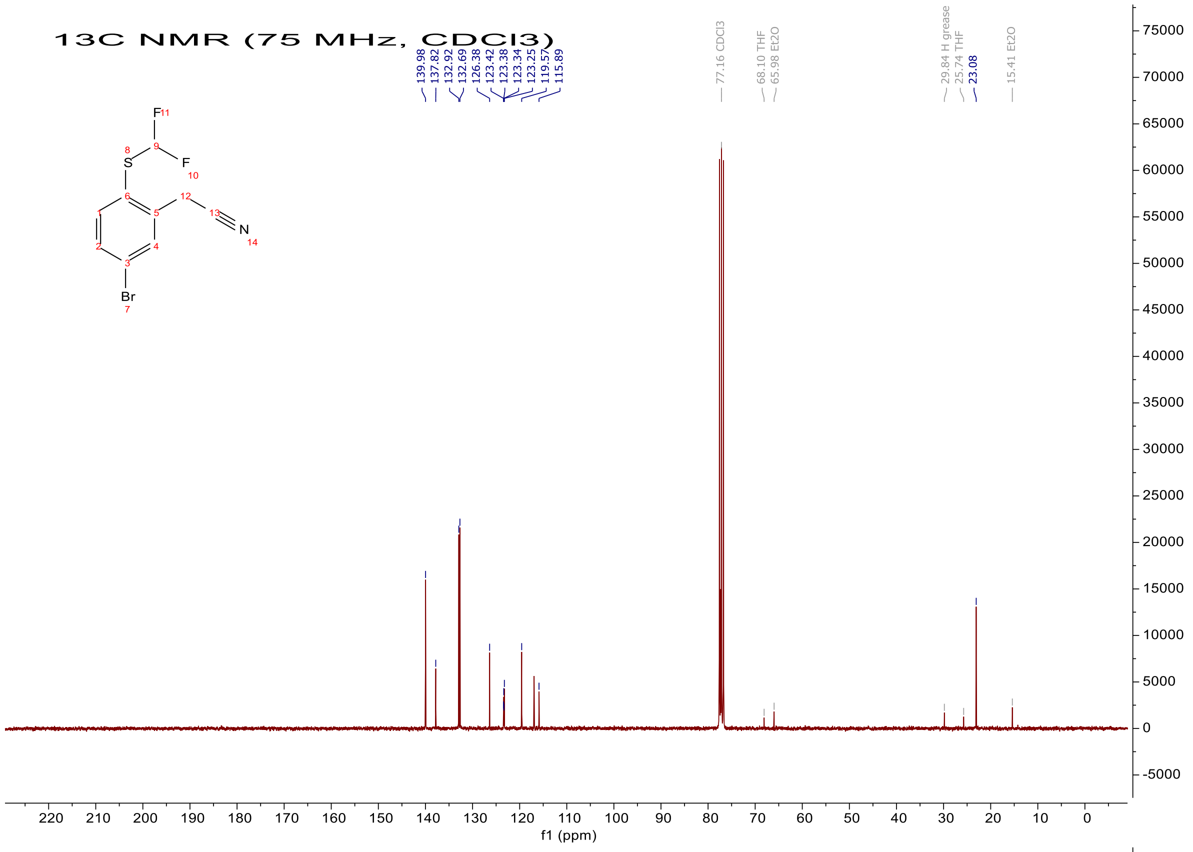
13C NMR (75 MHz, CDCl3)
139.98
137.82
132.92
132.69
126.38
123.42
123.38
123.34
123.25
119.57
115.89
77.16 CDCl3
68.10 THF
65.98 Et2O
29.84 H grease
25.74 THF
23.08
15.41 Et2O
f1 (ppm)

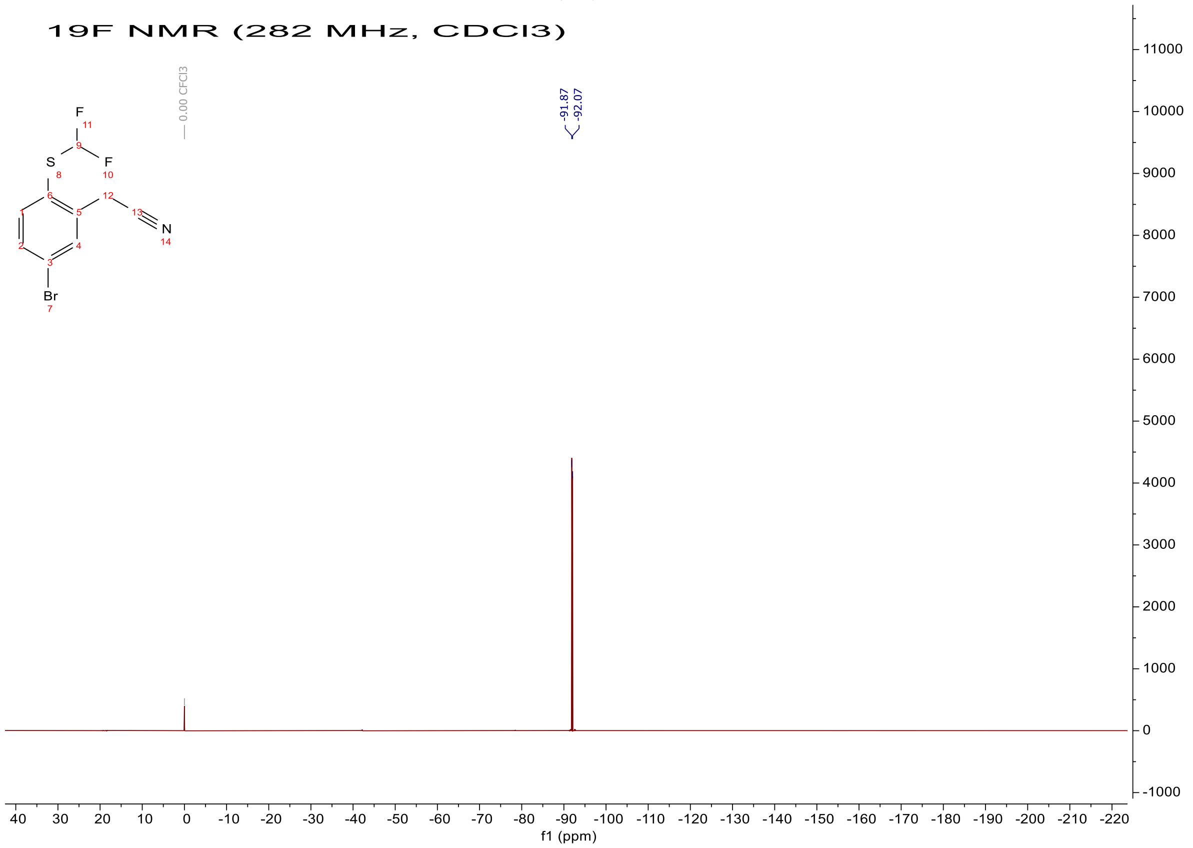
19F NMR (282 MHz, CDCl3)
0.00 CFCl3
-91.87
-92.07
f1 (ppm)

2-(2-((Difluoromethyl)thio)phenyl)acetonitrile (2i)

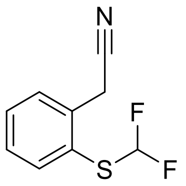

$C_9H_7F_2NS$
199.22

Reaction carried out following the general procedure 2 using ((difluoromethyl)sulfinyl)benzene (98 mg, 0.56 mmol). NMR ^{19}F yield = 60%. Purified by preparative TLC (*n*-pentane/EtOAc 84:16) affording the product as an orange oil (28 mg, 0.14 mmol). Yield = 25%. Highly volatile product.

* **HRMS ASAP+TOF**: Calculated for $C_9H_8F_2NS$ $[M+H]^+$: 200.0346; Found $[M+H]^+$: 200.0353.

* **^1H NMR (300 MHz, $CDCl_3$) δ (ppm):** 7.70 (dd, *J* = 8 Hz, 2 Hz, 1H), 7.63 (dd, *J* = 8 Hz, 2 Hz, 1H), 7.52 (td, *J* = 8 Hz, 2 Hz, 1H), 7.40 (td, *J* = 8 Hz, 2 Hz, 1H), 6.81 (t, *J* = 56 Hz, 1H), 4.04 (s, 2H).

* **^{13}C NMR (75 MHz, $CDCl_3$) *δ* (ppm):** 138.7, 135.9, 131.6, 129.9, 129.4, 124.6 (t, *J* = 3 Hz), 120.2 (t, *J* = 277 Hz), 117.6, 23.2.

* **^{19}F NMR (188 MHz, $CDCl_3$) δ (ppm):** -91.88 (d, *J* = 56 Hz).

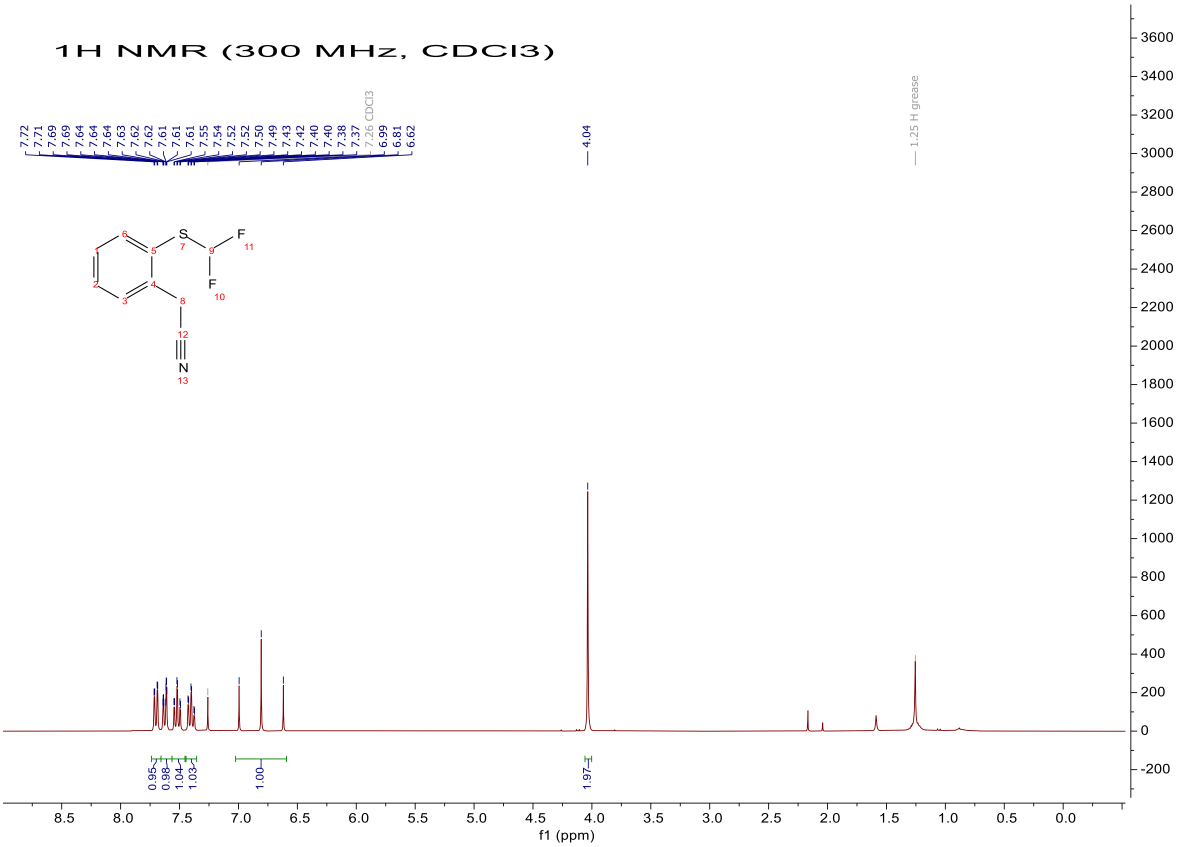

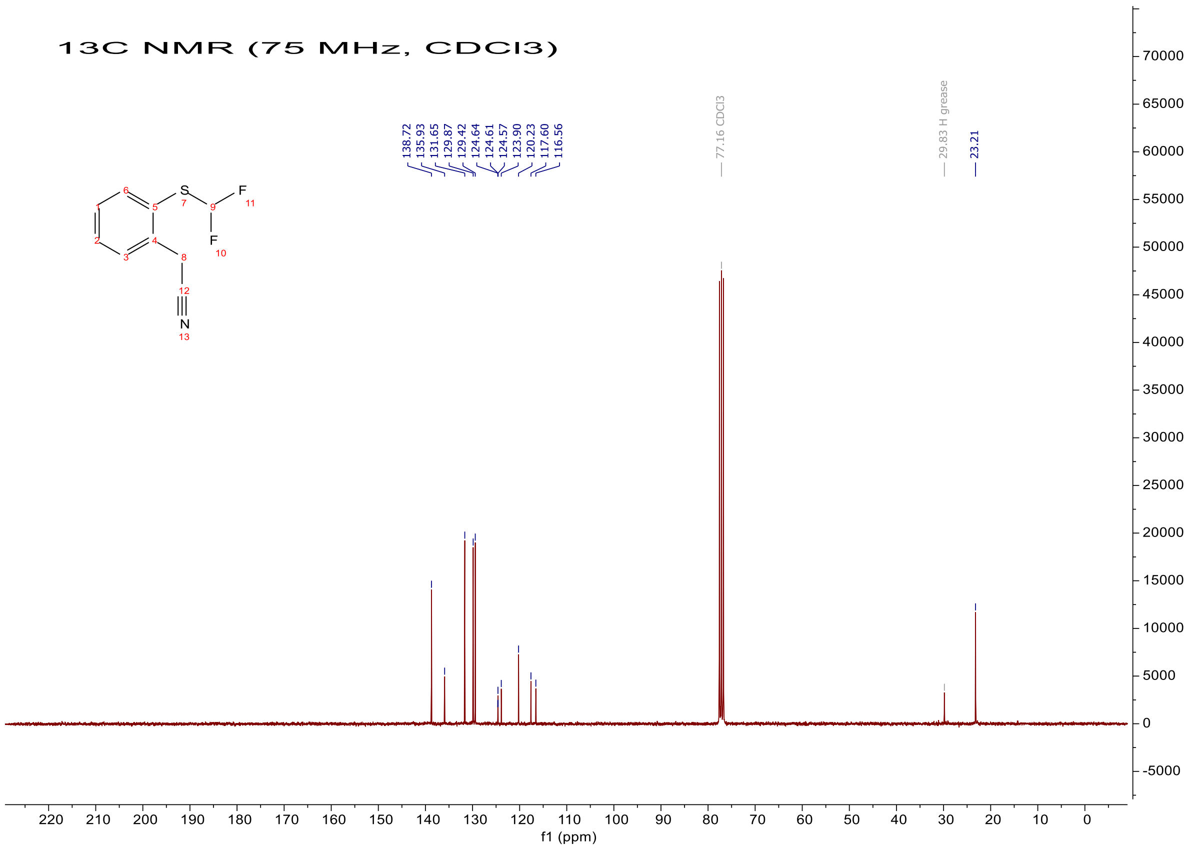
13C NMR (75 MHz, CDCl3)
138.72
135.93
131.65
129.87
129.42
124.64
124.61
124.57
123.90
120.23
117.60
116.56
77.16 CDCl3
29.83 H grease
23.21
f1 (ppm)

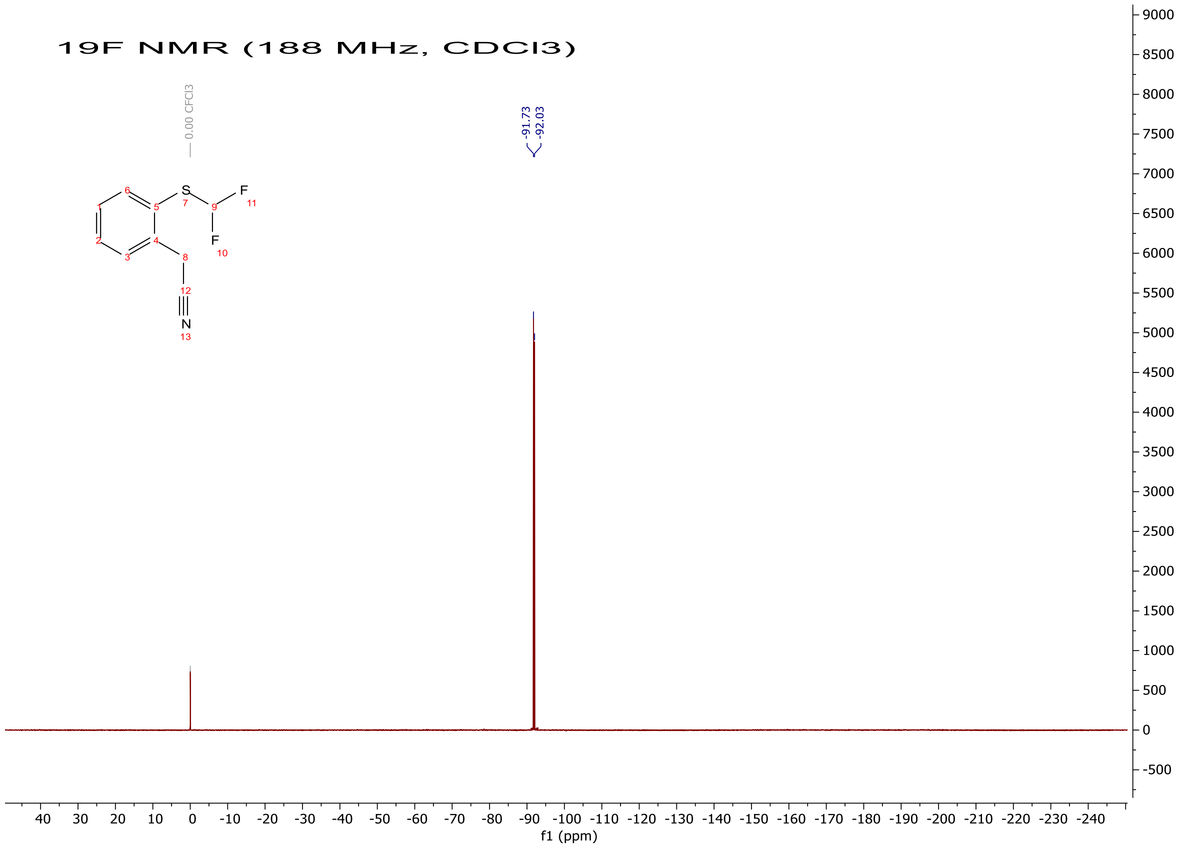
19F NMR (188 MHz, CDCl3)
0.00 CFCl3
-91.73
-92.03
f1 (ppm)

2-(2-((Bromodifluoromethyl)thio)phenyl)acetonitrile (2j)

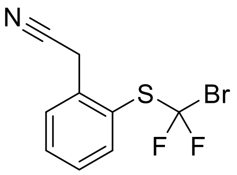

$C_9H_6BrF_2NS$
278.11

Reaction carried out following the general procedure 2 using ((bromodifluoromethyl)sulfinyl)benzene (128 mg, 0.50 mmol). NMR ^{19}F yield = 71%. Purified by preparative TLC (petroleum ether/diethyl ether 8:2), affording the product as an orange oil (92 mg, 0.33 mmol). Yield = 67%.

* **HRMS ASAP+TOF**: Calculated for $C_{18}H_{13}Br_2F_4N_2S_2[2M+H]^+$:554.8823 ; Found $[2M+H]^+$: 554.8822

* **^{1}H NMR (300 MHz, CDCl$_3$) δ (ppm):** 7.76 (dd, *J* = 8 Hz, 1 Hz, 1H), 7.68 (dd, *J* = 8 Hz, 1 Hz, 1H), 7.60 (td, *J* = 8 Hz, 1 Hz, 1H), 7.44 (td, *J* = 8 Hz, 1 Hz, 1H), 4.09 (s, 2H).

* **^{13}C NMR (75 MHz, CDCl$_3$) δ (ppm):** 139.3, 136.0, 132.8, 130.0, 129.6, 126.2, 118.6 (t, *J* = 339 Hz), 117.4, 23.1 (t, *J* = 1 Hz)

* **^{19}F NMR (282 MHz, CDCl$_3$) δ (ppm):** - 22.92 (s)

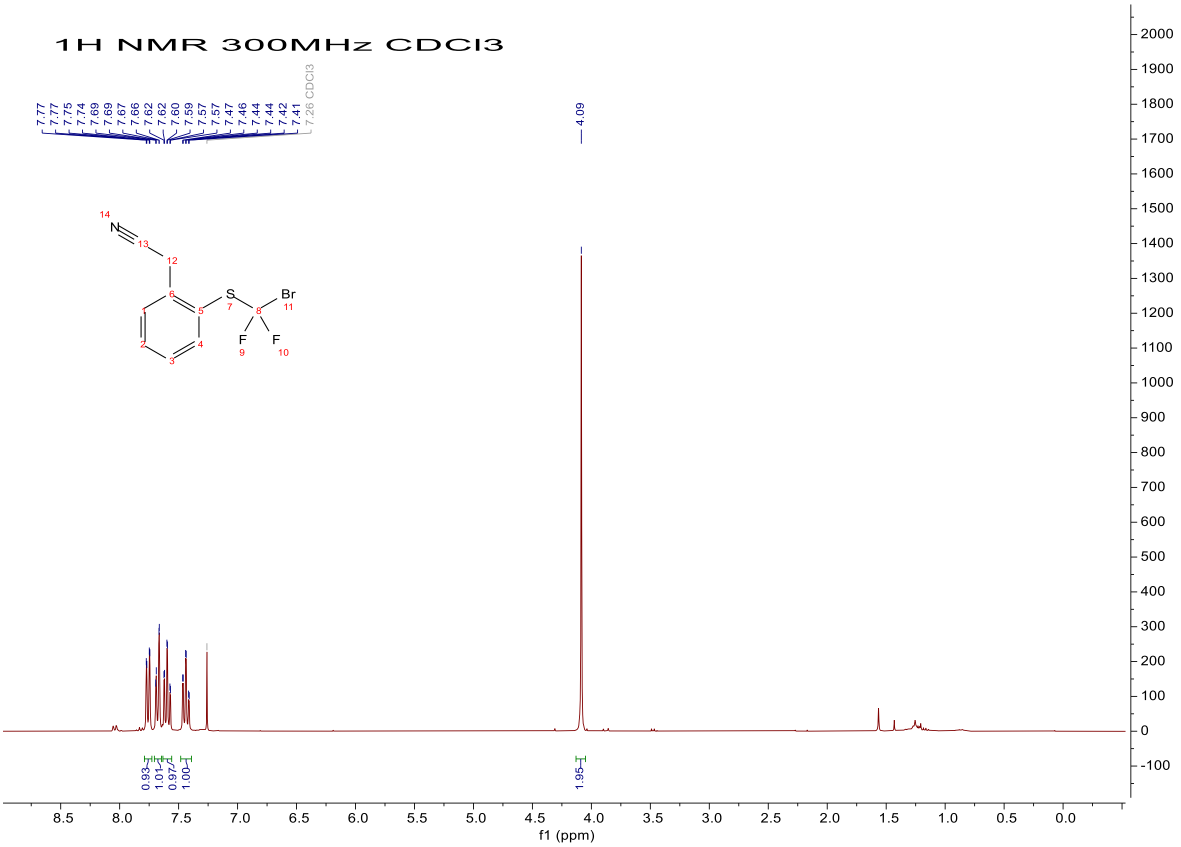

13C NMR 75 MHz CDCl3

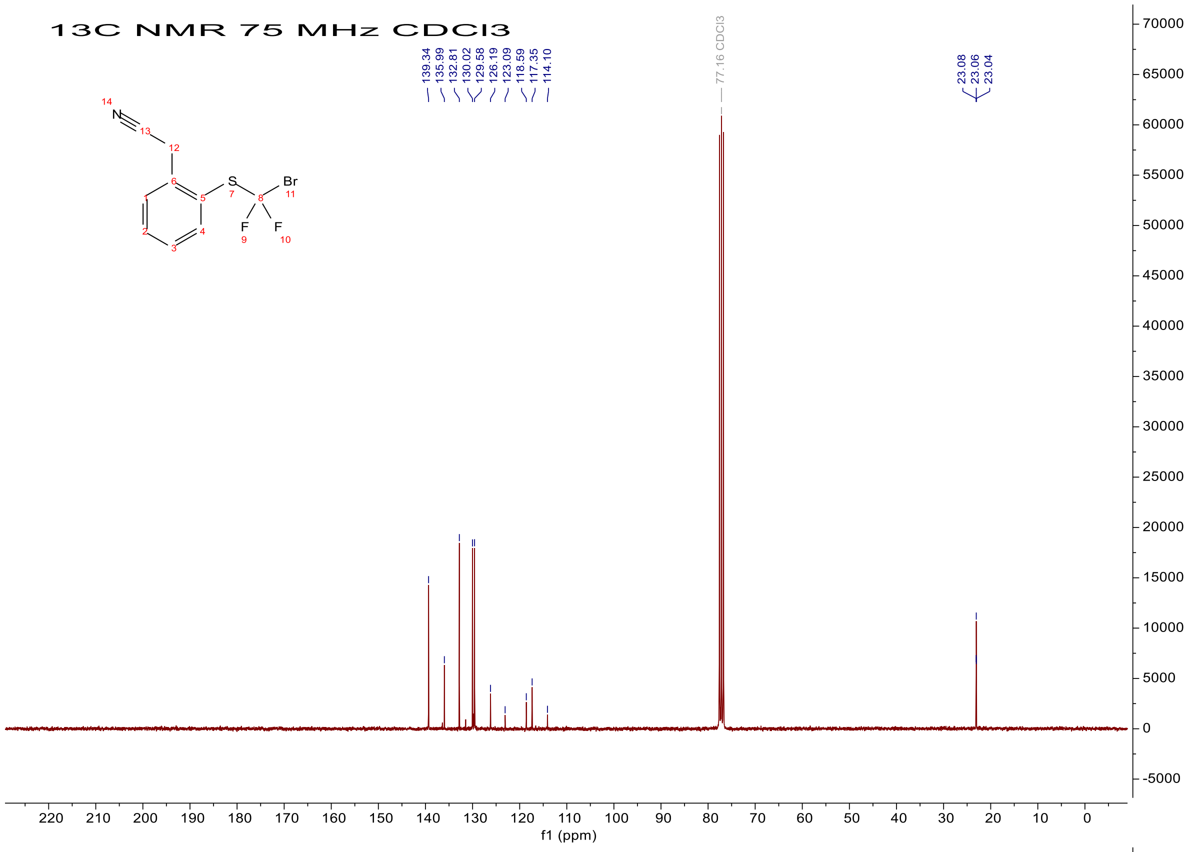

19F NMR 282 MHz CDCl3

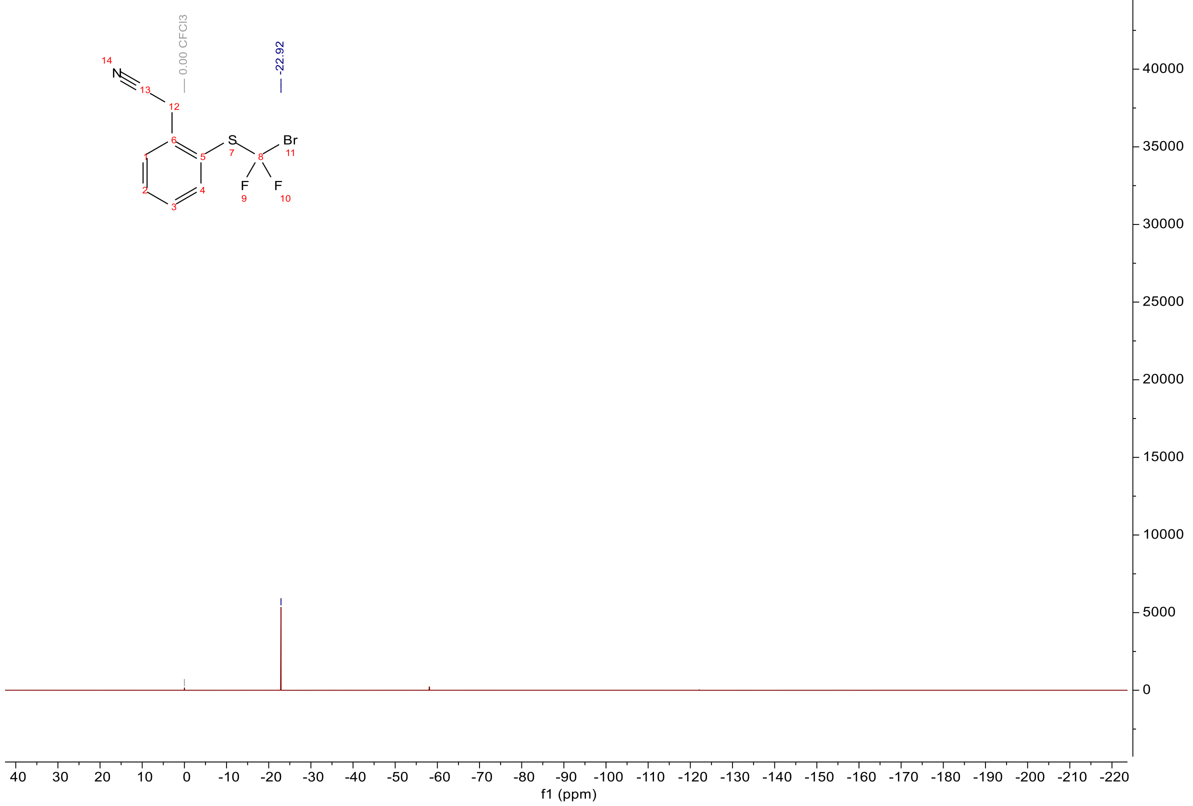

2-(2-((Trifluoromethyl)selanyl)phenyl)acetonitrile (2k)

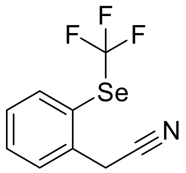

$C_9H_6F_3NSe$
264.12

Reaction carried out following the general procedure 2 using ((trifluoromethyl)seleninyl)benzene (93 mg, 0.39 mmol) with acetonitrile. NMR ^{19}F yield = 16%. Purified by flash chromatography (gradient: *n*-pentane/EtOAc 100:0 to 90:10), affording the product as an orange oil (15 mg, 0.06 mmol). Yield = 15%.

* **HRMS ASAP+TOF**: Calculated for $C_9H_7F_3NSe[M+H]^+$: pending; Found $[M+H]^+$: pending

* **^1H NMR (300 MHz, $CDCl_3$) δ (ppm):** 7.88 (dd, *J* = 8, 1 Hz, 1H), 7.68 (dd, *J* = 8, 1 Hz, 1H), 7.57 (td, *J* = 8, 1 Hz, 1H), 7.39 (td, *J* = 8, 1 Hz, 1H), 4.08 (s, 2H).

* **^{13}C NMR (75 MHz, $CDCl_3$) δ (ppm):** 140.3, 135.9, 132.4, 129.8, 129.7, 122.4 (d, *J* = 334 Hz), 117.5, 115.1, 25.4

* **^{19}F NMR (282 MHz, $CDCl_3$) δ (ppm):** -35.98 (s)

^{77}Se NMR (57 MHz, $CDCl_3$) δ (ppm): 501.99 (s)

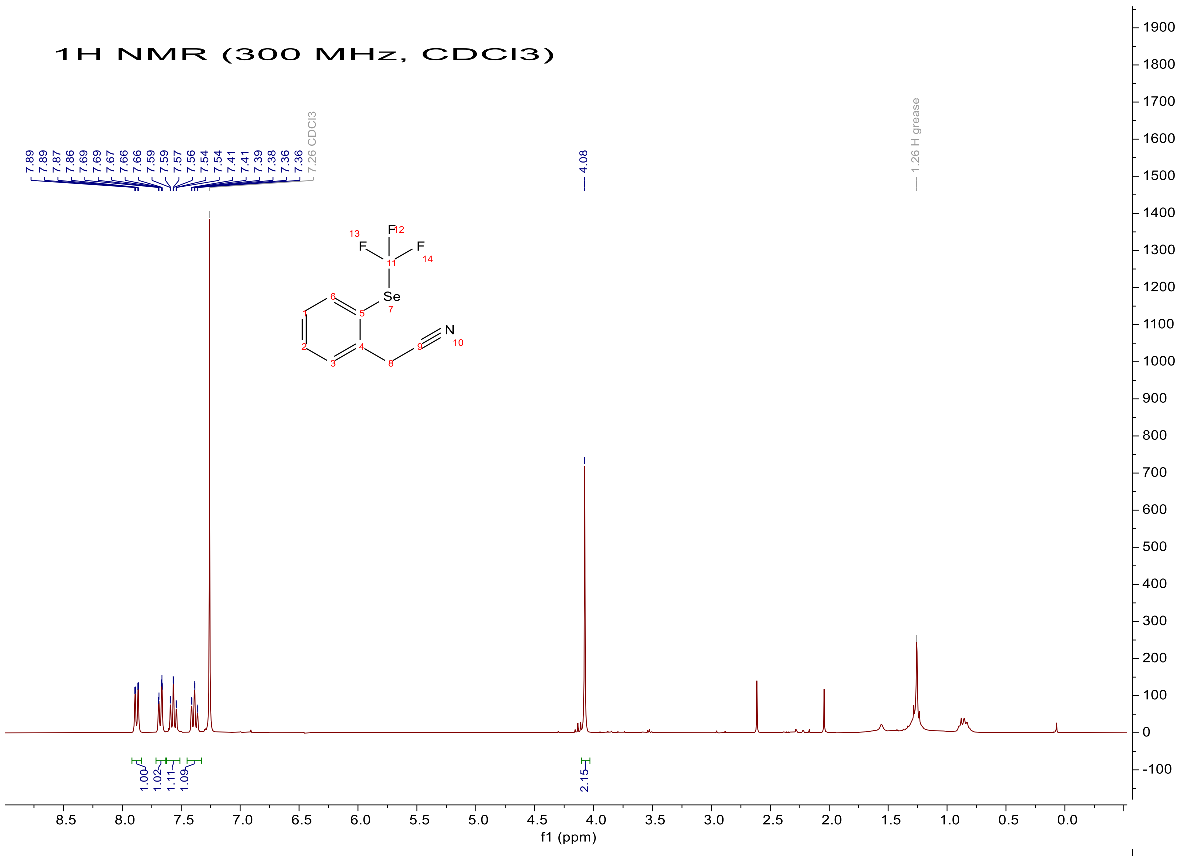
1H NMR (300 MHz, CDCl3)
7.89 7.89 7.87 7.86 7.69 7.69 7.67 7.66 7.59 7.59 7.57 7.56 7.54 7.54 7.41 7.39 7.38 7.36 7.36
7.26 CDCl3
4.08
1.26 H grease
1.00 1.02 1.11 1.09
2.15
f1 (ppm)

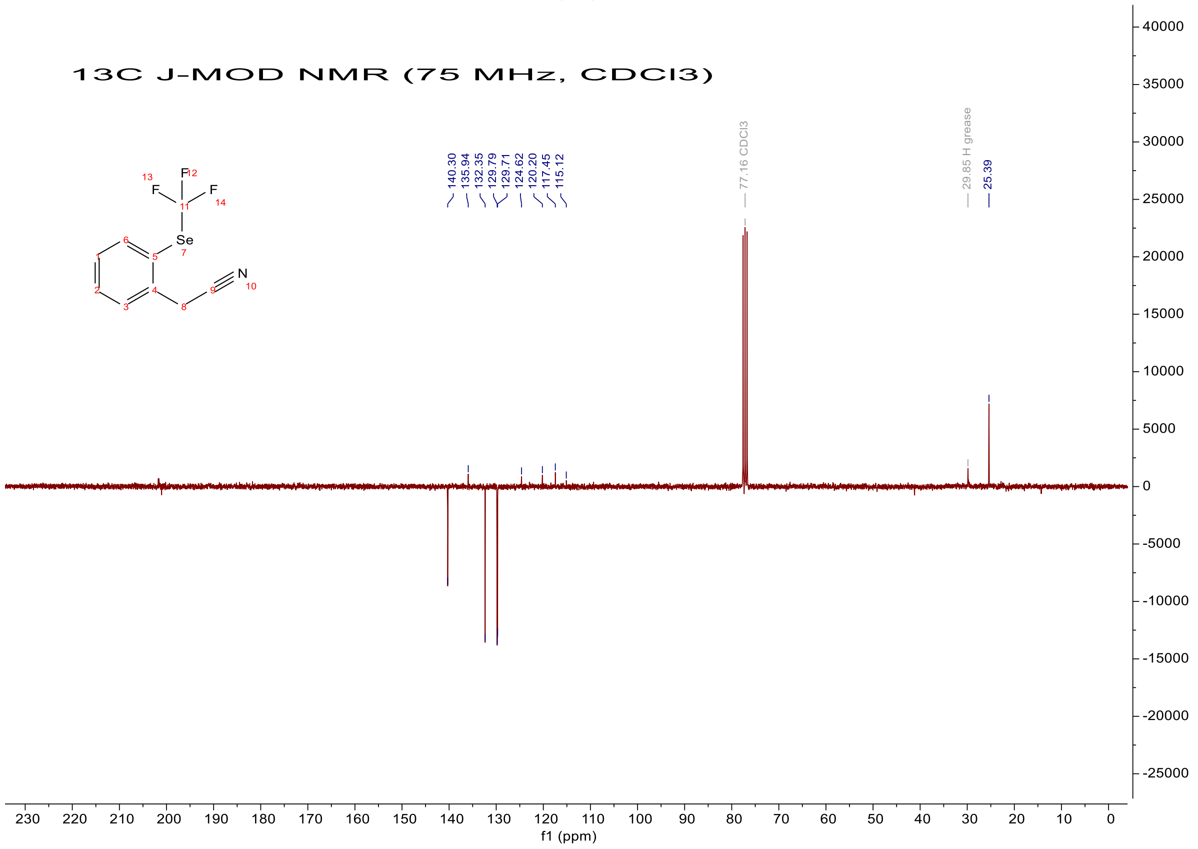
13C J-MOD NMR (75 MHz, CDCl3)
140.30 135.94 132.35 129.79 129.71 124.62 120.20 117.45 115.12
77.16 CDCl3
29.85 H grease
25.39
f1 (ppm)

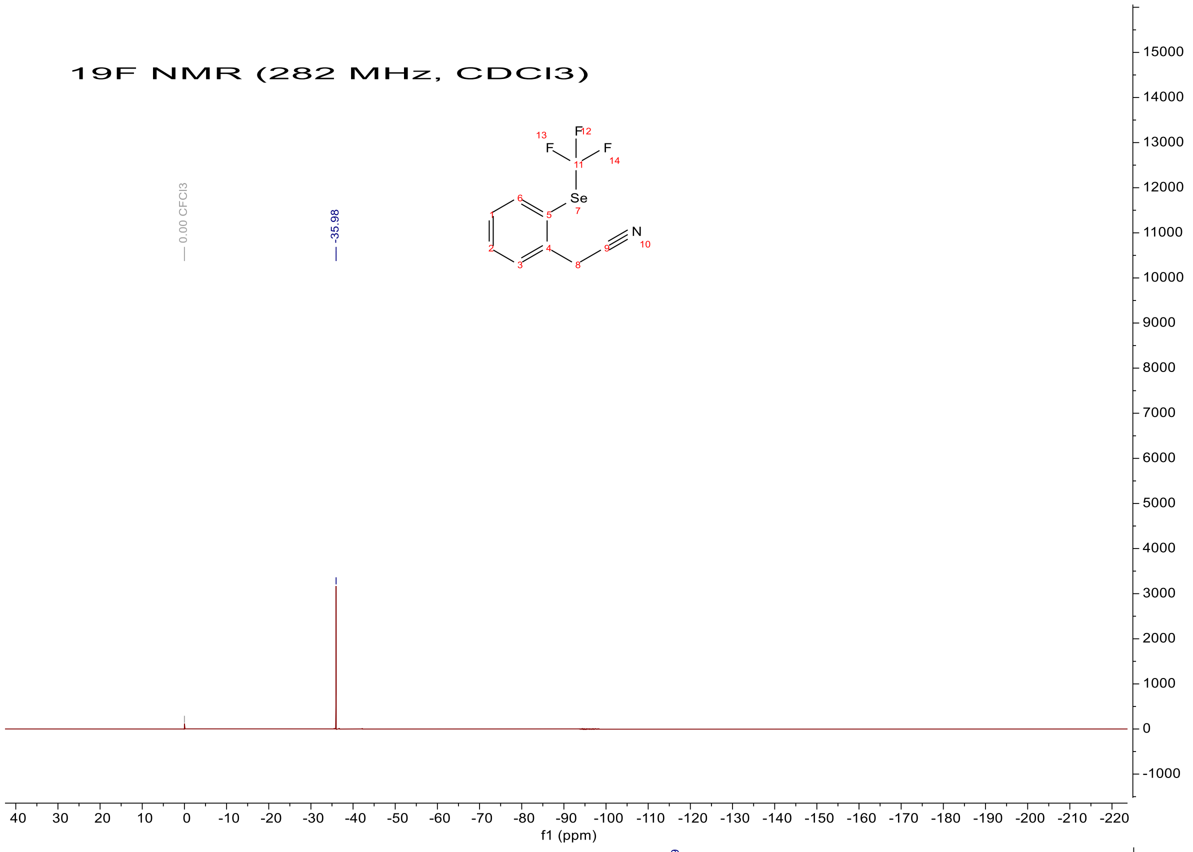
19F NMR (282 MHz, CDCl3)
0.00 CFCl3
-35.98
f1 (ppm)

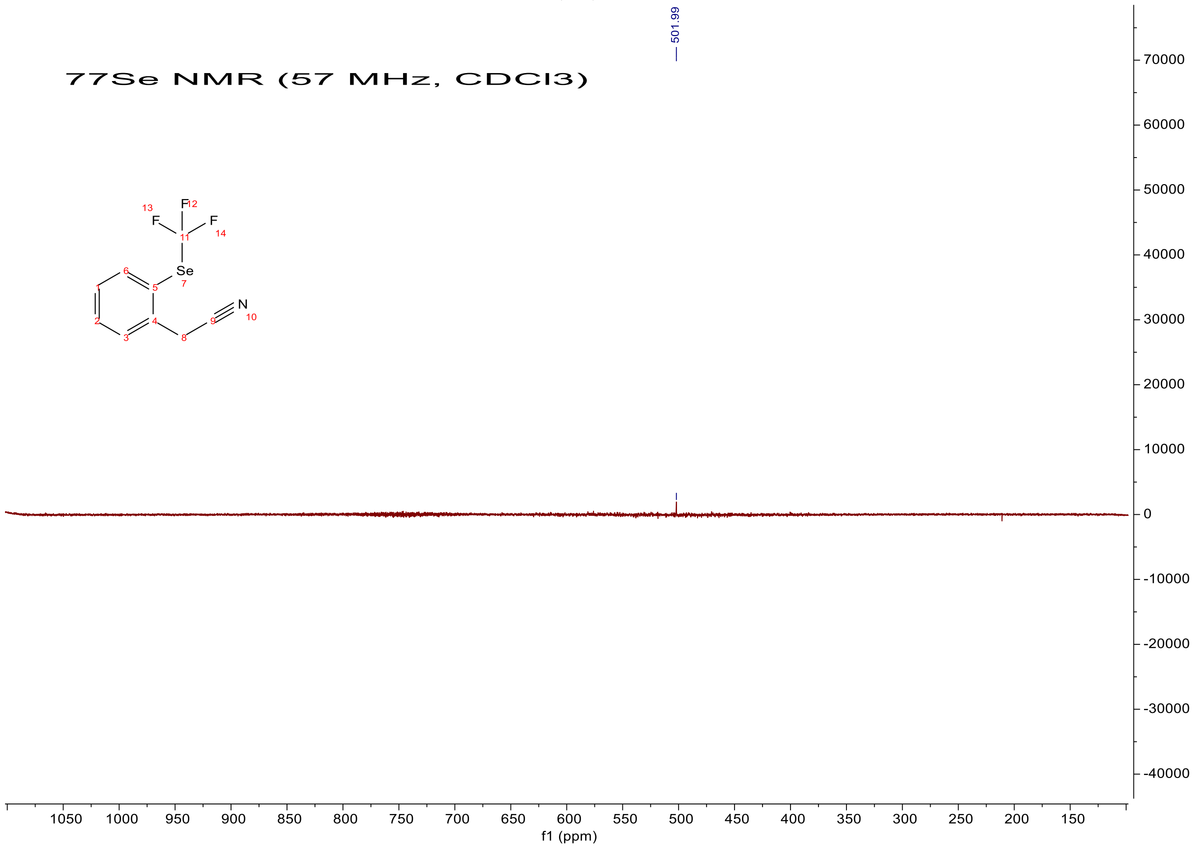
77Se NMR (57 MHz, CDCl3)
501.99
f1 (ppm)

2-(2-((Trifluoromethyl)thio)phenyl)propanenitrile (3a)

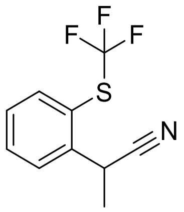

$C_{10}H_8F_3NS$
231.24

Reaction carried out following the general procedure 2 using propionitrile and ((trifluoromethyl)sulfinyl)benzene(97 mg, 0.5 mmol) and propionitrile. NMR ^{19}F yield = 87%. Purified by preparative TLC (petroleum ether/diethyl ether 7:3) affording the product as a yellow oil (83 mg, 0.36 mmol). Yield = 72%.

* **HRMS ASAP+TOF**: Calculated for $C_{10}H_9F_3NS[M+H]^+$:232.0408 ; Found $[M+H]^+$: 232.0412.

* **^1H NMR (300 MHz, $CDCl_3$) δ (ppm):** 7.76 – 7.70 (m, 2H), 7.60 (td, J = 8 Hz, 2 Hz, 1H), 7.41 (td, J = 8 Hz, 2 Hz, 1H), 4.69 (q, J = 7 Hz, 1H), 1.63 (d, J = 7 Hz, 3H).

* **^{13}C NMR (75 MHz, $CDCl_3$) *δ* (ppm):** 143.1, 139.2, 132.8, 129.4, 129.1 (q, J = 309 Hz), 128.7, 122.5 (q, J = 2 Hz), 121.4, 29.4, 21.4

* **^{19}F NMR (282 MHz, $CDCl_3$) δ (ppm):** -42.89 (s)

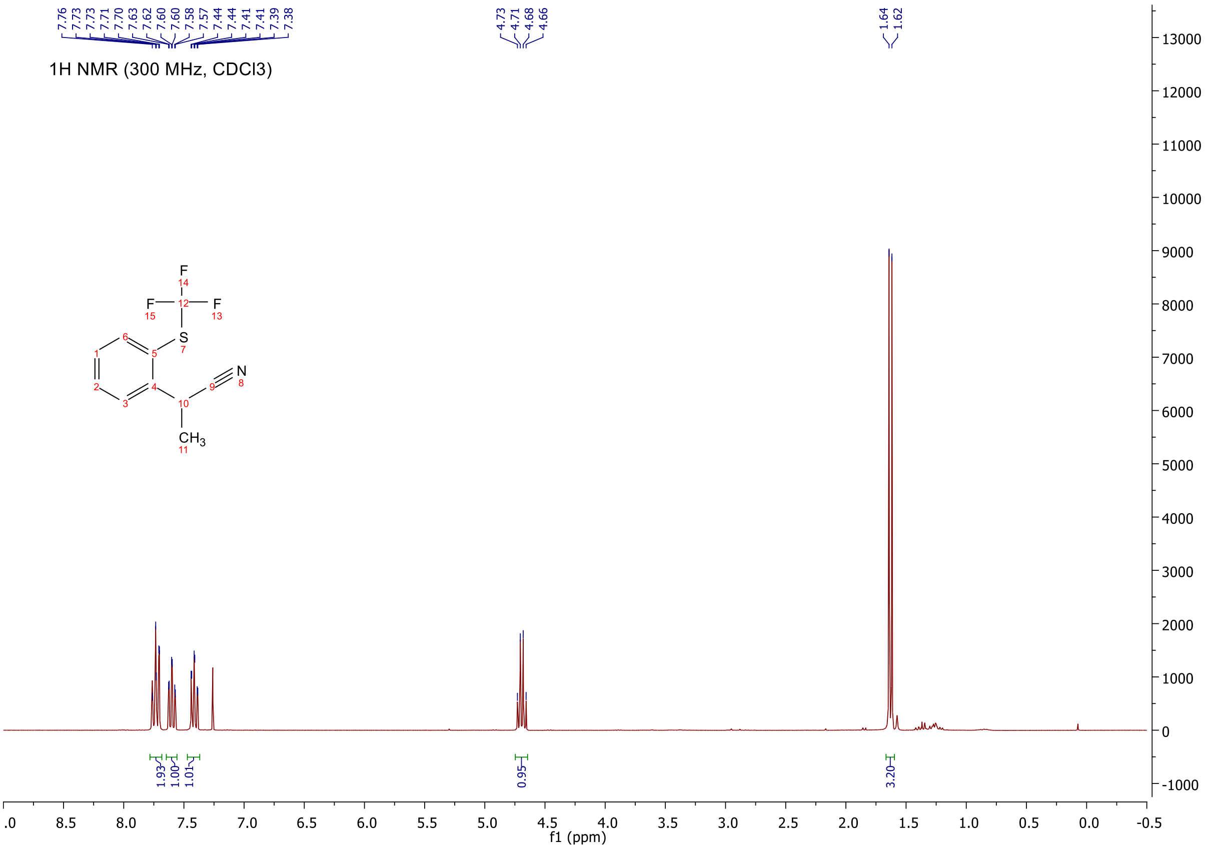

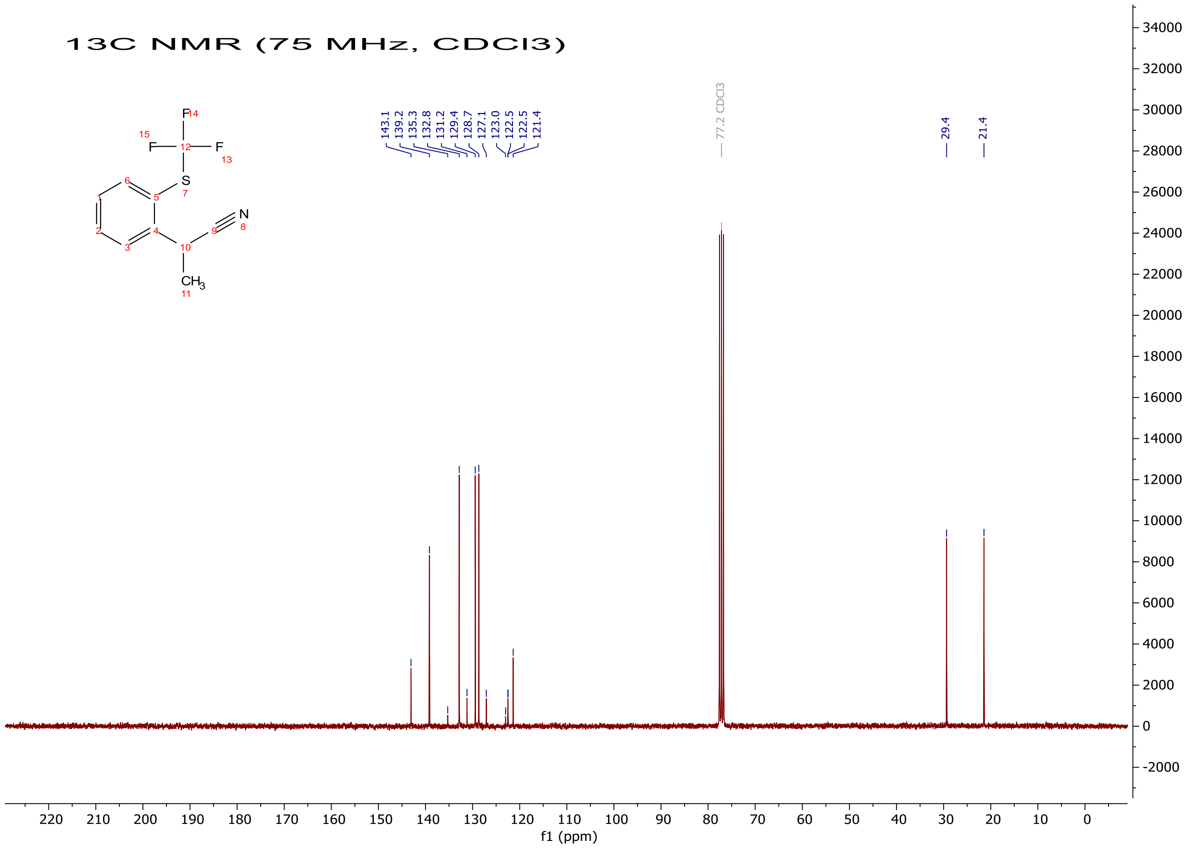
13C NMR (75 MHz, CDCl3)
143.1
139.2
135.3
132.8
131.2
129.4
128.7
127.1
123.0
122.5
122.5
121.4
77.2 CDCl3
29.4
21.4
f1 (ppm)

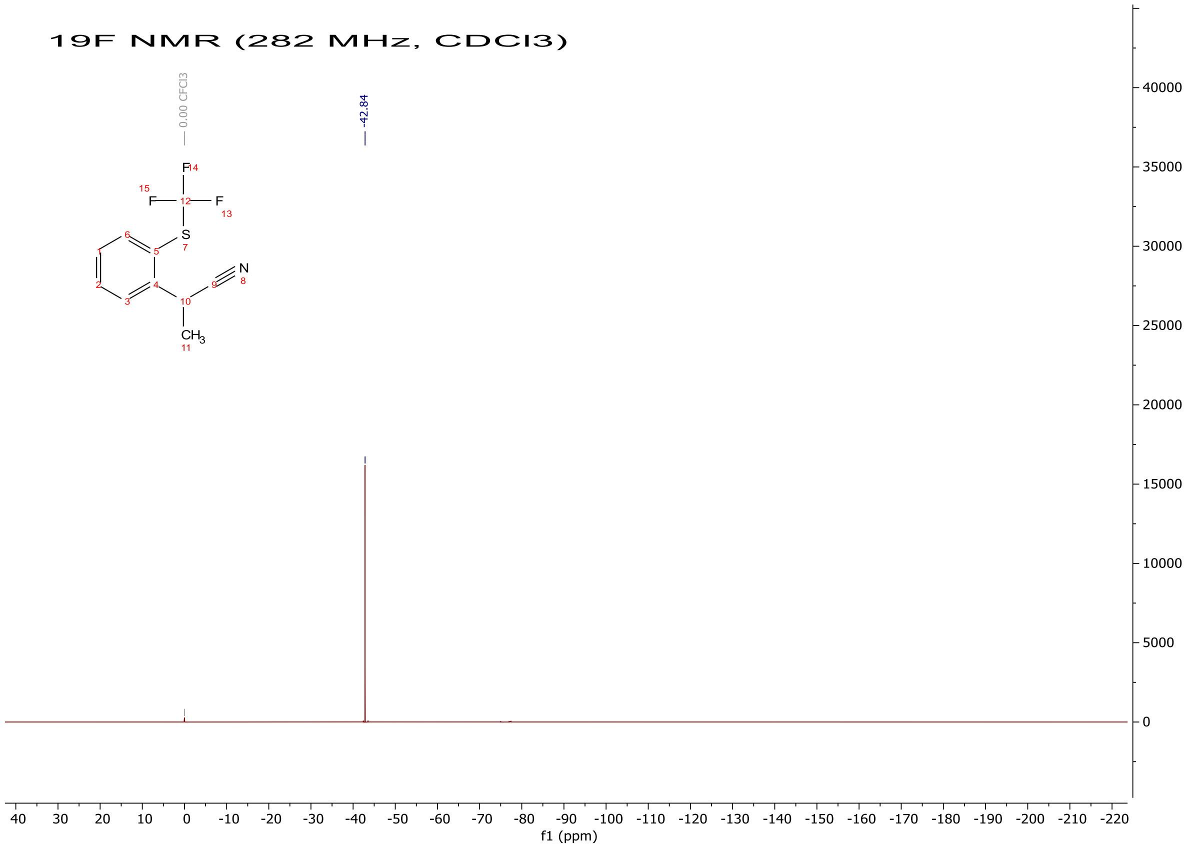
19F NMR (282 MHz, CDCl3)
0.00 CFCl3
-42.84
f1 (ppm)

2-(2-((Trifluoromethyl)thio)phenyl)butanenitrile (3b)

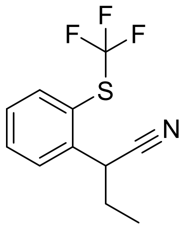

$C_{11}H_{10}F_3NS$
MW: 245.26

Reaction carried out following the general procedure 2 using ((trifluoromethyl)sulfinyl)benzene (97 mg, 0.5 mmol) and butyronitrile. NMR ^{19}F yield = 80%. Purified by : firstly, preparative TLC (petroleum ether/diethyl ether 7:3), secondly, flash chromatography (*n*-pentane/EtOAc 100:0 to 88:12 gradient) affording the product as a yellow oil (73 mg, 0.3 mmol). Yield = 60%.

* **HRMS ASAP+TOF**: Calculated for $C_{22}H_{21}F_6N_2S_2$ $[2M+H]^+$:491.1050; Found $[2M+H]^+$: 491.1049.

* **^{1}H NMR (300 MHz, CDCl$_3$) δ (ppm):** 7.75 (d, *J* = 8 Hz, 1H), 7.68 (d, *J* = 8 Hz, 1H), 7.58 (t, *J* = 8 Hz, 1H), 7.40 (t, *J* = 8 Hz, 1H), 4.54 (t, *J* = 7 Hz, 1H), 1.98 – 1.93 (m, 2H), 1.13 (t, *J* = 7 Hz, 3H).

* **^{13}C NMR (75 MHz, CDCl$_3$) *δ* (ppm):**

141.8, 139.1, 132.5, 129.3, 129.1 (q, *J* = 309 Hz), 122.9 (q, *J* = 2 Hz), 120.5, 36.8, 29.3, 11.7

* **^{19}F NMR (282 MHz, CDCl$_3$) δ (ppm):** -42.82 (s)

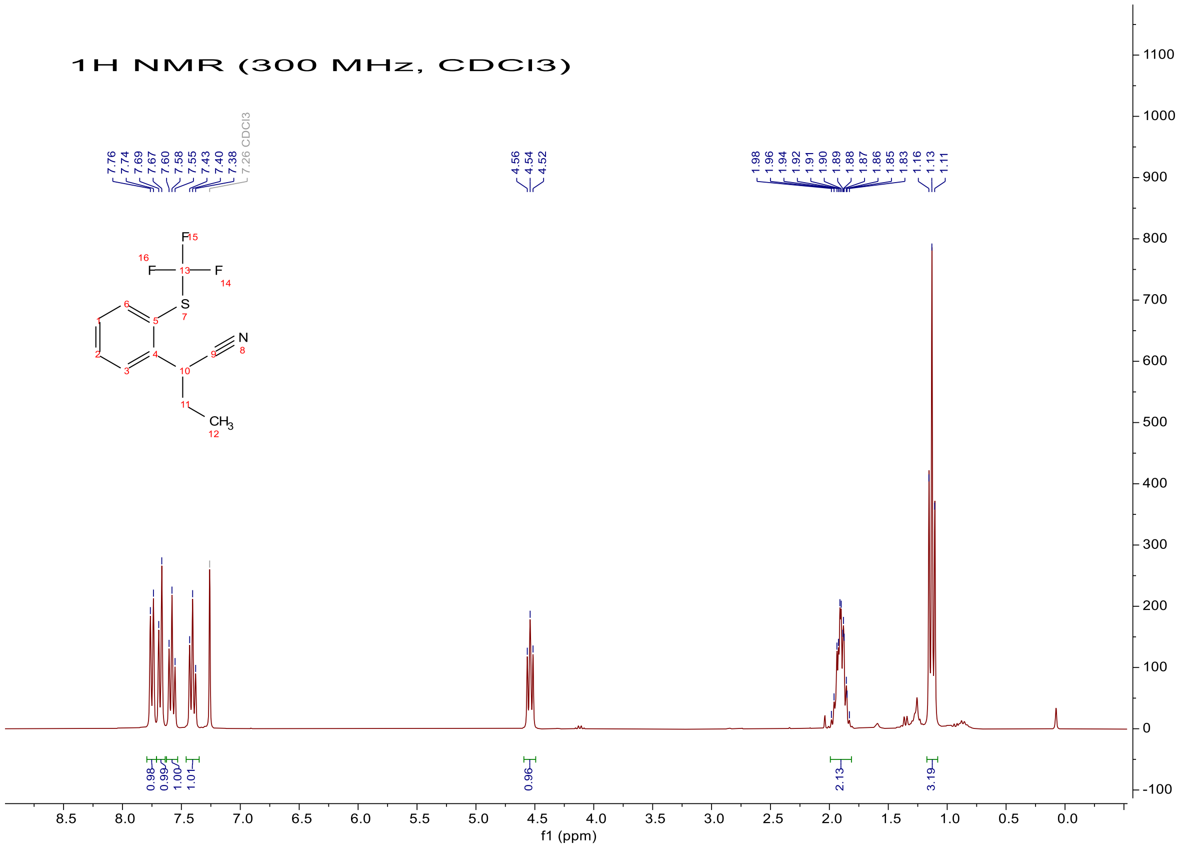
1H NMR (300 MHz, CDCl3)
f1 (ppm)

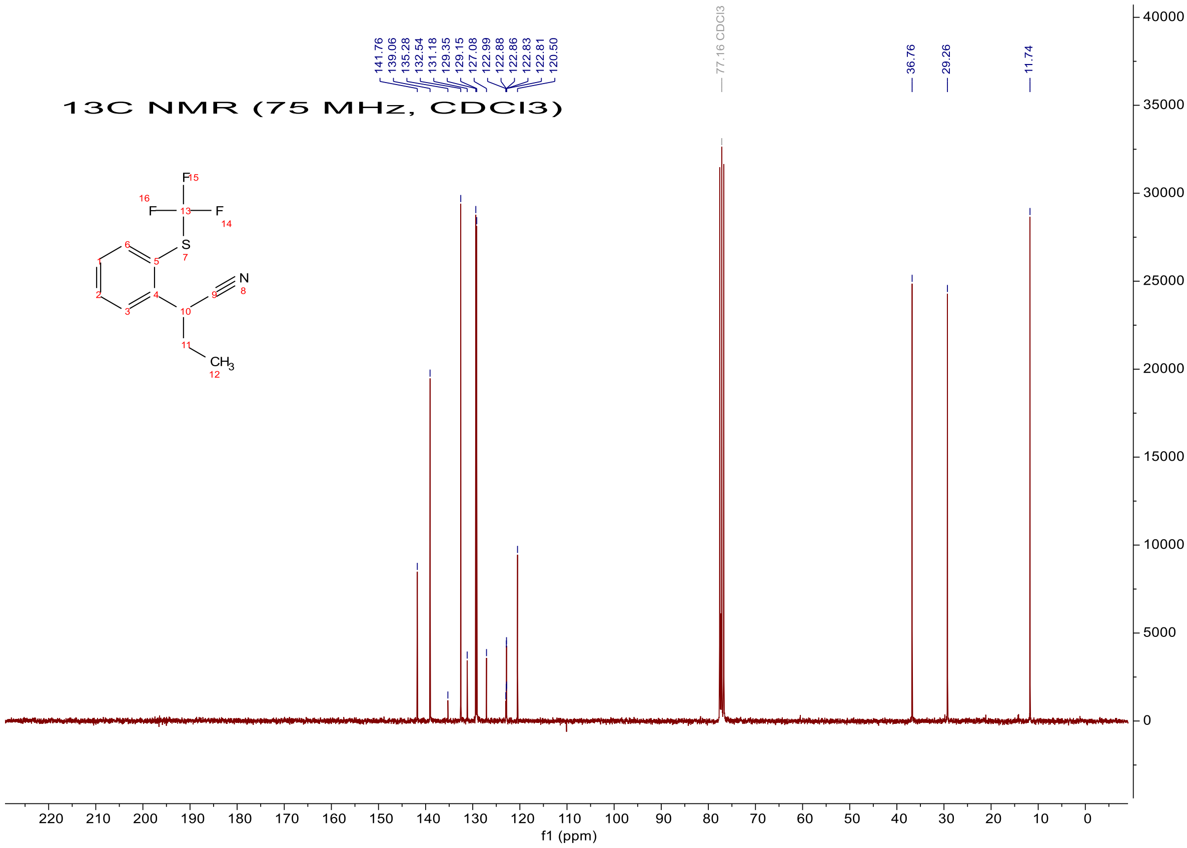
13C NMR (75 MHz, CDCl3)
f1 (ppm)

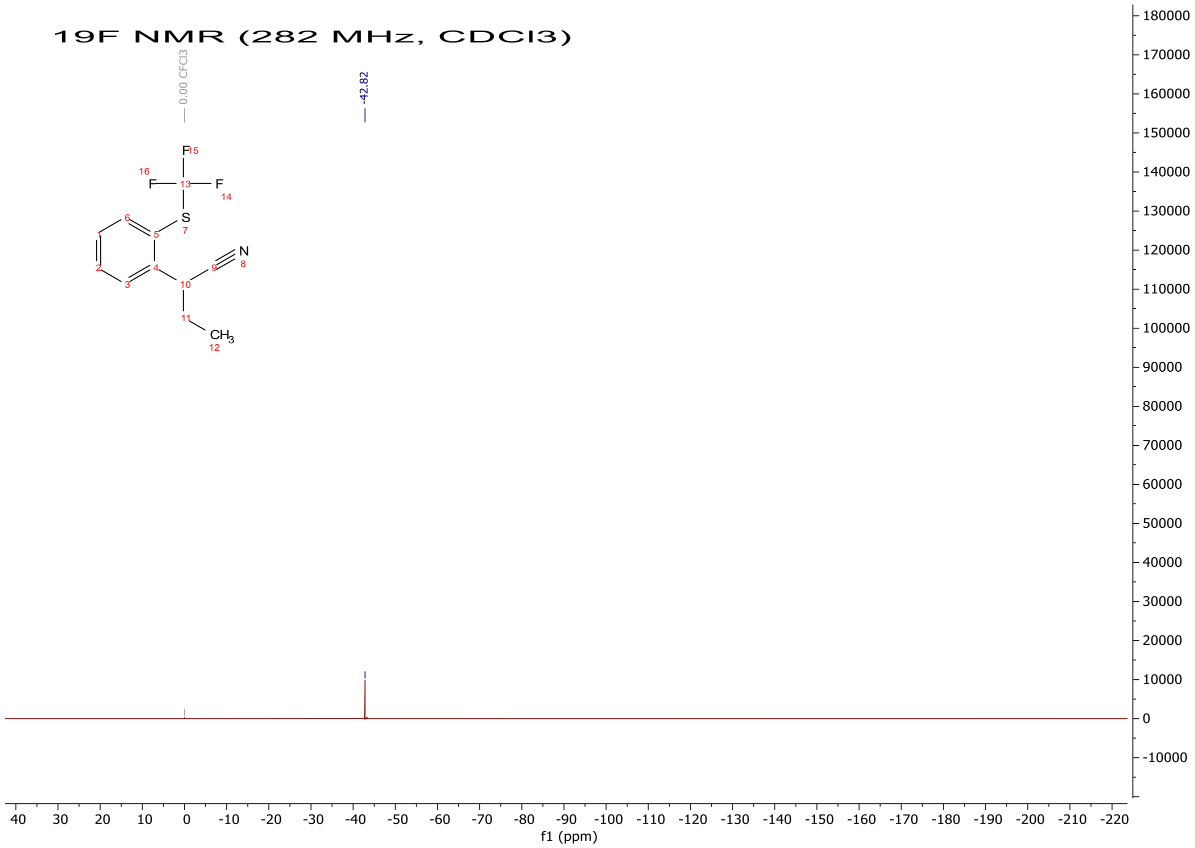

19F NMR (282 MHz, CDCl3)
0.00 CFCl3
-42.82
f1 (ppm)

2-Chloro-2-(2-((trifluoromethyl)thio)phenyl)acetonitrile (3d)

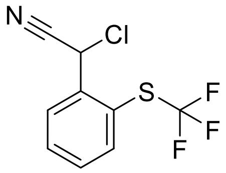

$C_9H_5ClF_3NS$
251.65

Reaction carried out following the general procedure 2 using ((trifluoromethyl)sulfinyl)benzene (97 mg, 0.5 mmol) with chloroacetonitrile. NMR ^{19}F yield = 28%. Purified by flash chromatography (gradient: petroleum ether/diethyl ether 100:0 to 90:10), affording the product as an orange oil (34 mg, 0.14 mmol). Yield = 27%.

* **HRMS ASAP+TOF**: Calculated for $C_9H_6ClF_3NS[M+H]^+$: 251.9862; Found $[M+H]^+$: 251.9850

* **^1H NMR (300 MHz, $CDCl_3$) δ (ppm):** 7.98 (dd, *J* = 8, 1 Hz, 1H), 7.81 (d, *J* = 8 Hz, 1H), 7.69 (td, *J* = 8, 1 Hz, 1H), 7.55 (td, *J* = 8, 2 Hz, 1H), 6.29 (s, 1H).

* **^{13}C NMR (75 MHz, $CDCl_3$) *δ* (ppm):** 139.2, 138.8, 133.1, 131.7, 130.0, 128.8 (q, *J* = 310 Hz), 123.3 (q, *J* = 2 Hz), 115.9, 41.9

* **^{19}F NMR (282 MHz, $CDCl_3$) δ (ppm):** -42.57 (s)

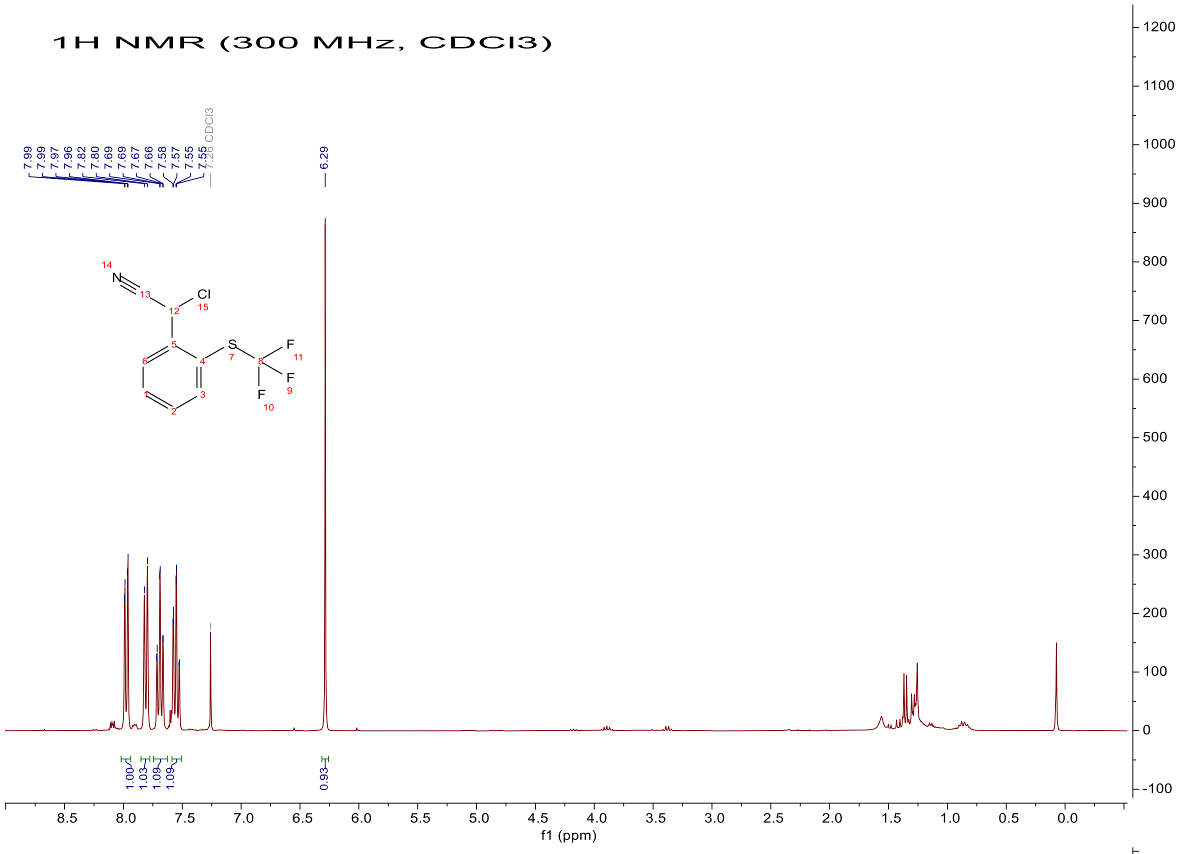
1H NMR (300 MHz, CDCl3)
7.99
7.99
7.97
7.96
7.82
7.80
7.69
7.69
7.67
7.66
7.58
7.57
7.55
7.55
7.26 CDCl3
6.29
1.00
1.03
1.09
1.09
0.93
f1 (ppm)

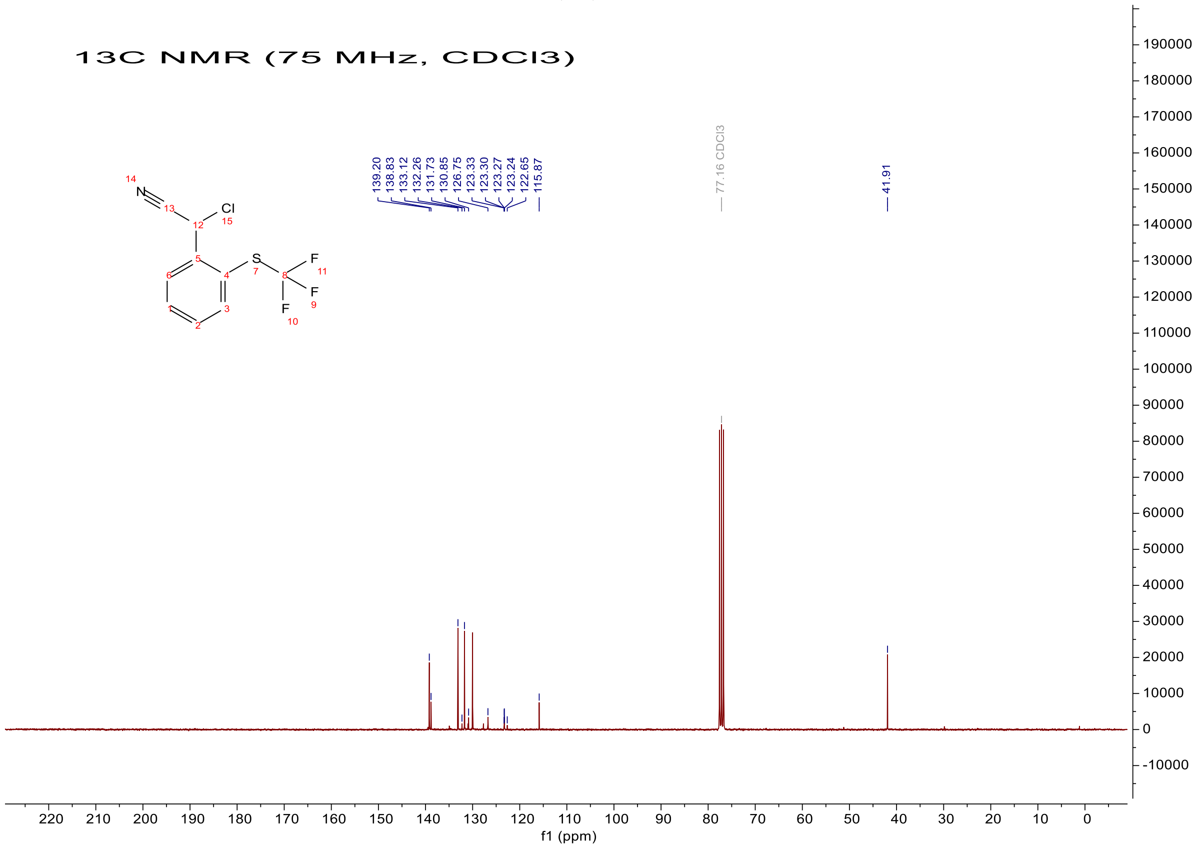
13C NMR (75 MHz, CDCl3)
139.20
138.83
133.12
132.26
131.73
130.85
126.75
123.33
123.30
123.27
123.24
122.65
115.87
77.16 CDCl3
41.91
f1 (ppm)

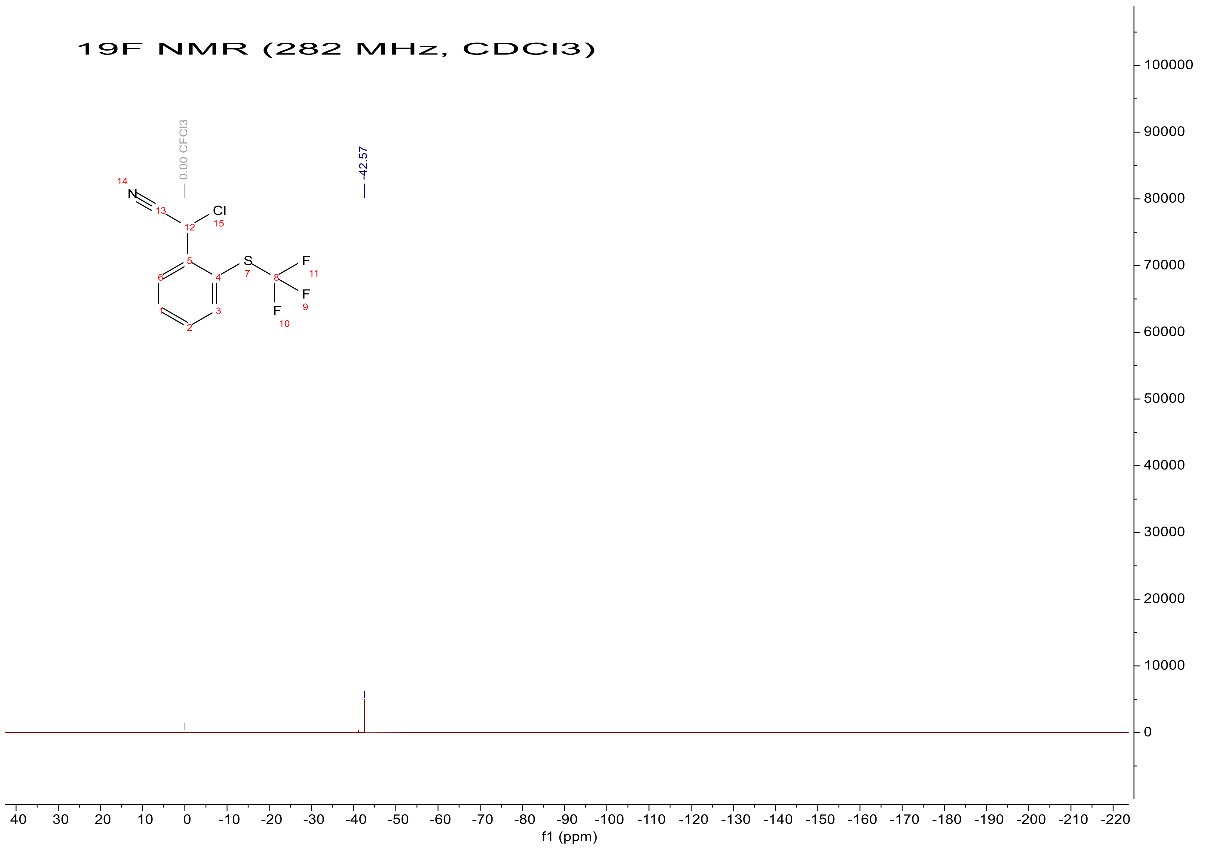
19F NMR (282 MHz, CDCl3)
0.00 CFCl3
-42.57
N
Cl
S
F
F
F
f1 (ppm)

5-Bromo-2-(2-((trifluoromethyl)thio)phenyl)pentanenitrile (3e)

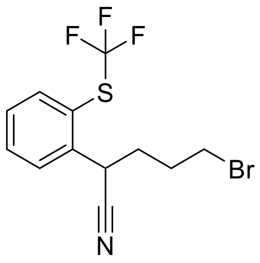

$C_{12}H_{11}BrF_3NS$
338.19

Reaction carried out following the general procedure 2 using (trifluoromethyl)sulfinyl)benzene (97 mg, 0.5 mmol) and 5-bromopentanenitrile. NMR ^{19}F yield = 52%. Purified by preparative TLC (petroleum ether/diethyl ether 8:2) affording the product as an orange oil (54 mg, 0.16 mmol). Yield = 32%.

* **HRMS ASAP+TOF**: Calculated for $C_{12}H_{12}BrF_3NS$ $[M+H]^+$: 337.9826 ; Found $[M+H]^+$: 337.9838.

* **^1H NMR (300 MHz, $CDCl_3$) δ (ppm):** 7.77 (d, *J* = 8 Hz, 1H), 7.70 (dd, *J* = 8, 2 Hz, 1H), 7.60 (td, *J* = 8, 2 Hz, 1H), 7.43 (td, *J* = 8, 2 Hz, 1H), 4.64 (t, *J* = 7 Hz, 1H), 3.44 (t, *J* = 6 Hz, 2H), 2.24 – 1.98 (m, 4H).

* **^{13}C NMR (75 MHz, $CDCl_3$) *δ* (ppm):** 141.3, 139.2, 132.8, 129.7, 129.1, 129.0 (q, *J* = 309 Hz), 122.9 (q, *J* = 2 Hz), 120.1, 34.6, 34.3, 32.0, 30.1

* **^{19}F NMR (188 MHz, $CDCl_3$) δ (ppm):** -42.72 (s)

1H NMR (300 MHz, CDCl3)

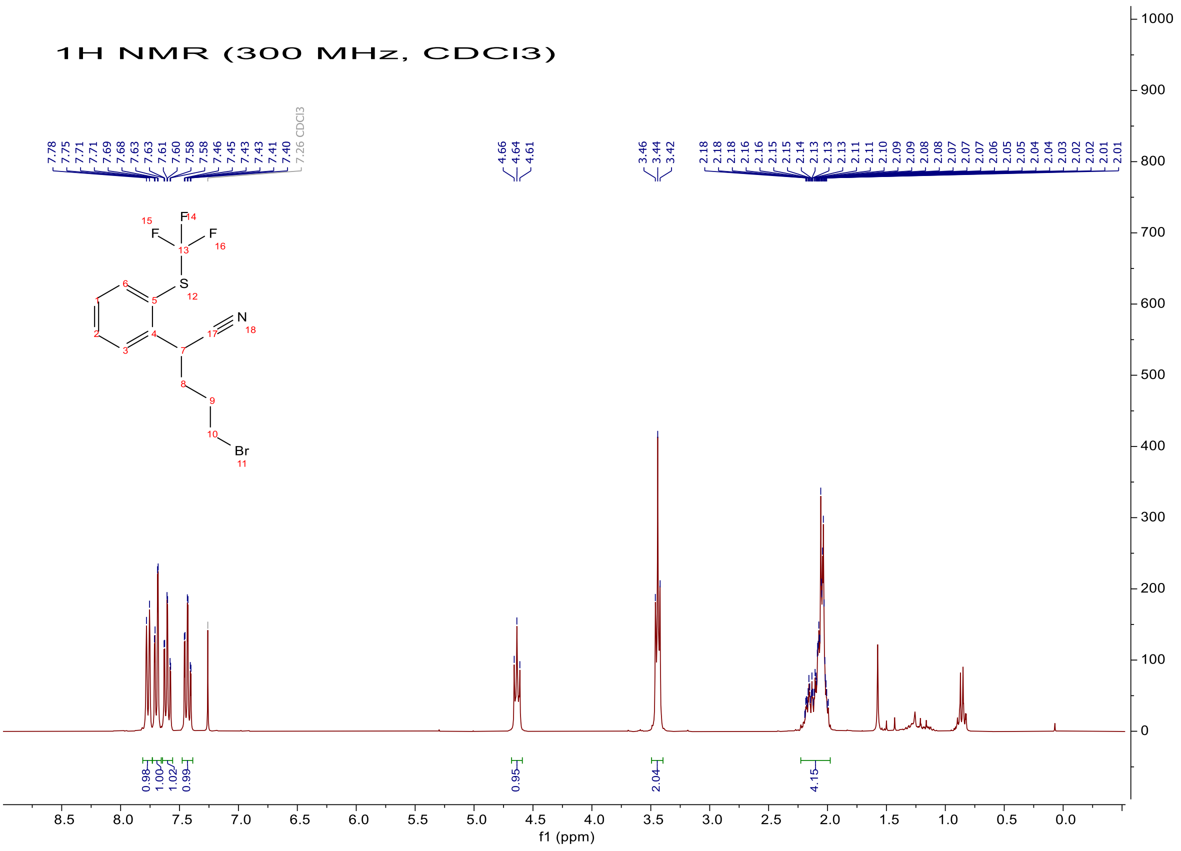

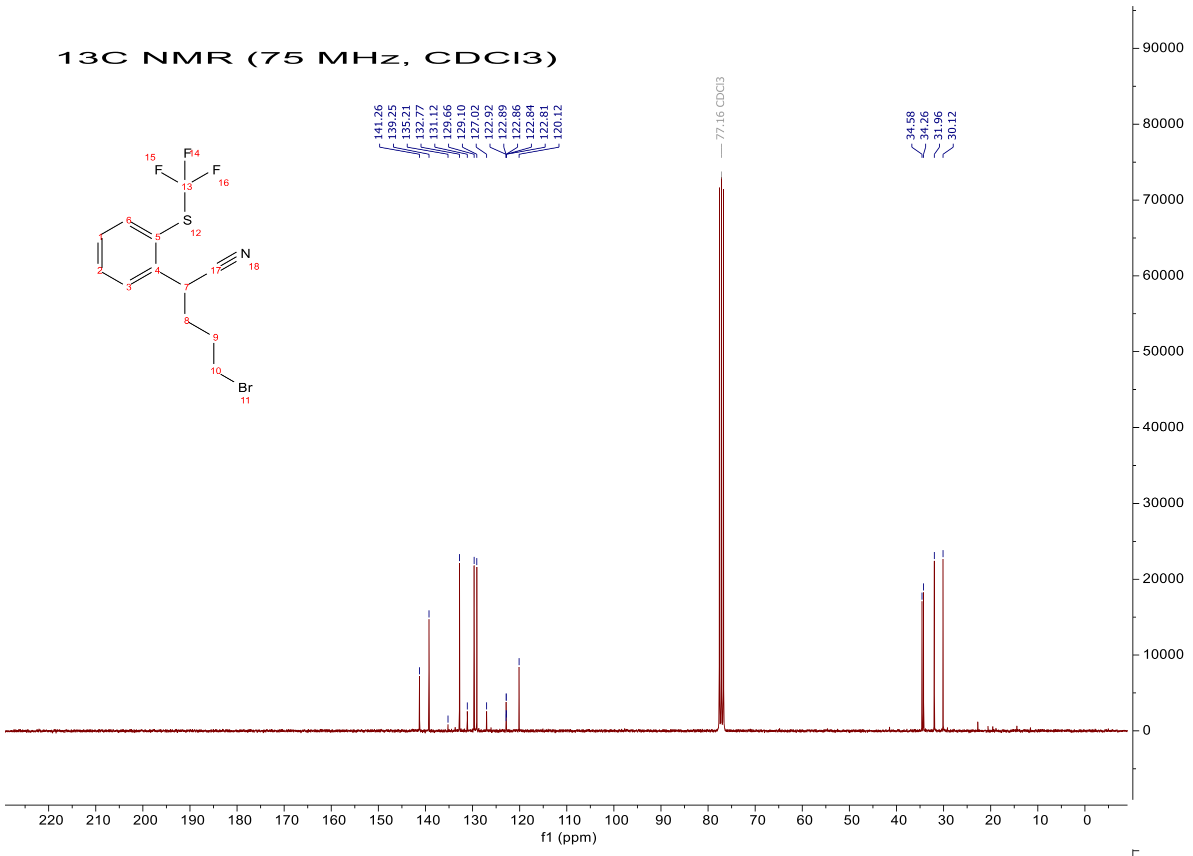
13C NMR (75 MHz, CDCl3)
141.26
139.25
135.21
132.77
131.12
129.66
129.10
127.02
122.92
122.89
122.86
122.84
122.81
120.12
77.16 CDCl3
34.58
34.26
31.96
30.12
F 14
F 15
F 16
13
S 12
N 18
17
Br 11
f1 (ppm)

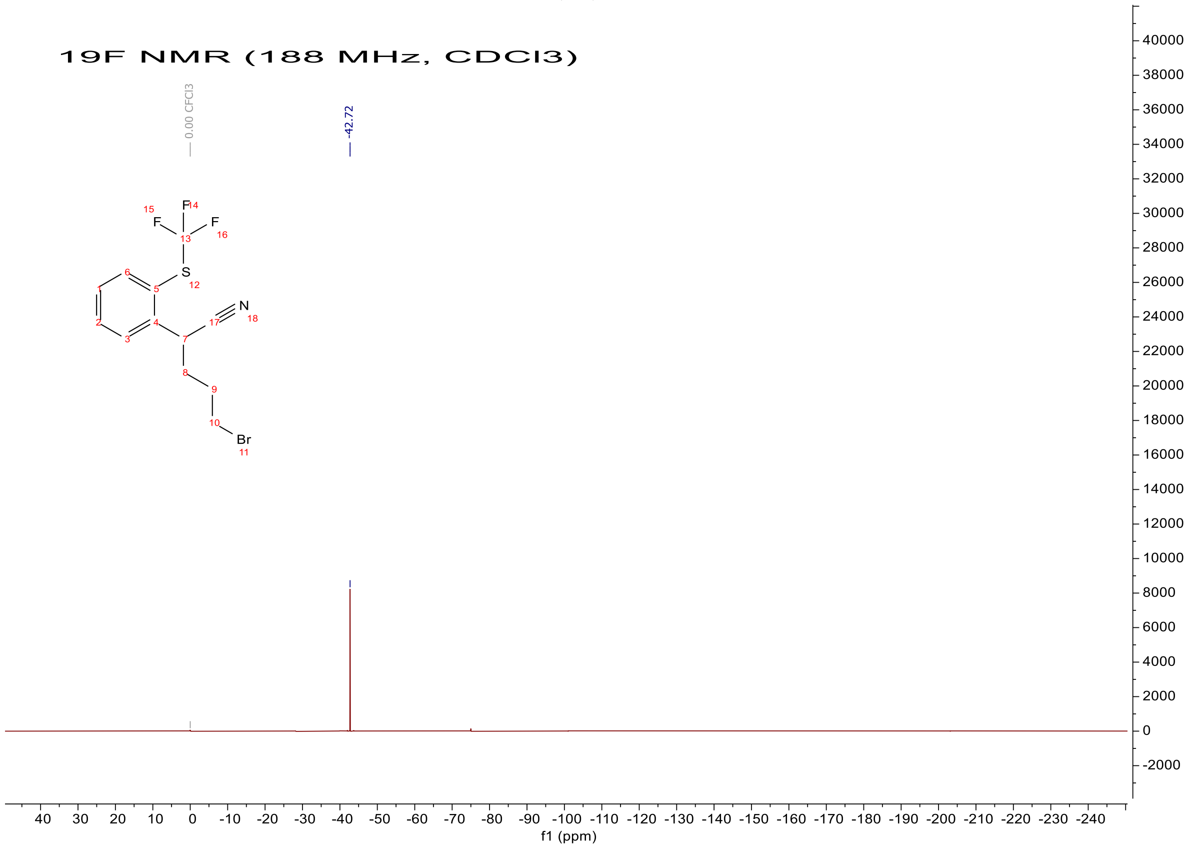
19F NMR (188 MHz, CDCl3)
0.00 CFCl3
-42.72
f1 (ppm)

2-(2-((Trifluoromethyl)thio)phenyl)hex-5-enenitrile (3f)

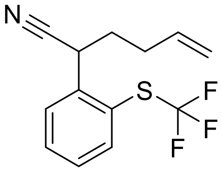

$C_{13}H_{12}F_3NS$
271.30

Reaction carried out following the general procedure 2 using ((Trifluoromethyl)sulfinyl)benzene (97 mg, 0.5 mmol) with hex-5-enenitrile. 0.3 mL of dichloromethane was used as solvent. NMR ^{19}F yield = 63%. Purified by preparative TLC (1st elution: petroleum ether/diethyl ether 7:3; 2nd elution: petroleum ether/diethyl ether 8:2; 3rd elution *n*-pentane/diethyl ether 9:1), affording the product as a yellow oil (79 mg, 0.29 mmol). Yield = 58%.

* **HRMS ASAP+TOF**: Calculated for $C_{23}H_{25}F_6N_2S_2$[2M+H]$^+$: 543.1363; Found [2M+H]$^+$: 543.1343

* **^{1}H NMR (300 MHz, CDCl$_3$) δ (ppm):** 7.75 (d, *J* = 8 Hz, 1H), 7.69 (dd, *J* = 8, 2 Hz, 1H), 7.59 (td, *J* = 8, 2 Hz, 1H), 7.41 (td, *J* = 8, 2 Hz, 1H), 5.86 – 5.72 (m, 1H), 5.18 – 5.06 (m, 2H), 4.62 (dd, *J* = 10, 5 Hz, 1H), 2.44 – 2.19 (m, 2H), 2.10 – 1.81 (m, 2H).

* **^{13}C NMR (75 MHz, CDCl$_3$) *δ* (ppm):** 141.8, 139.1, 135.8, 132.7, 129.4, 129.2, 129.1 (q, *J* = 308 Hz), 122.81 (q, *J* = 2 Hz), 120.4, 117.0, 34.9, 34.6, 31.4

* **^{19}F NMR (188 MHz, CDCl$_3$) δ (ppm):** -42.75 (s)

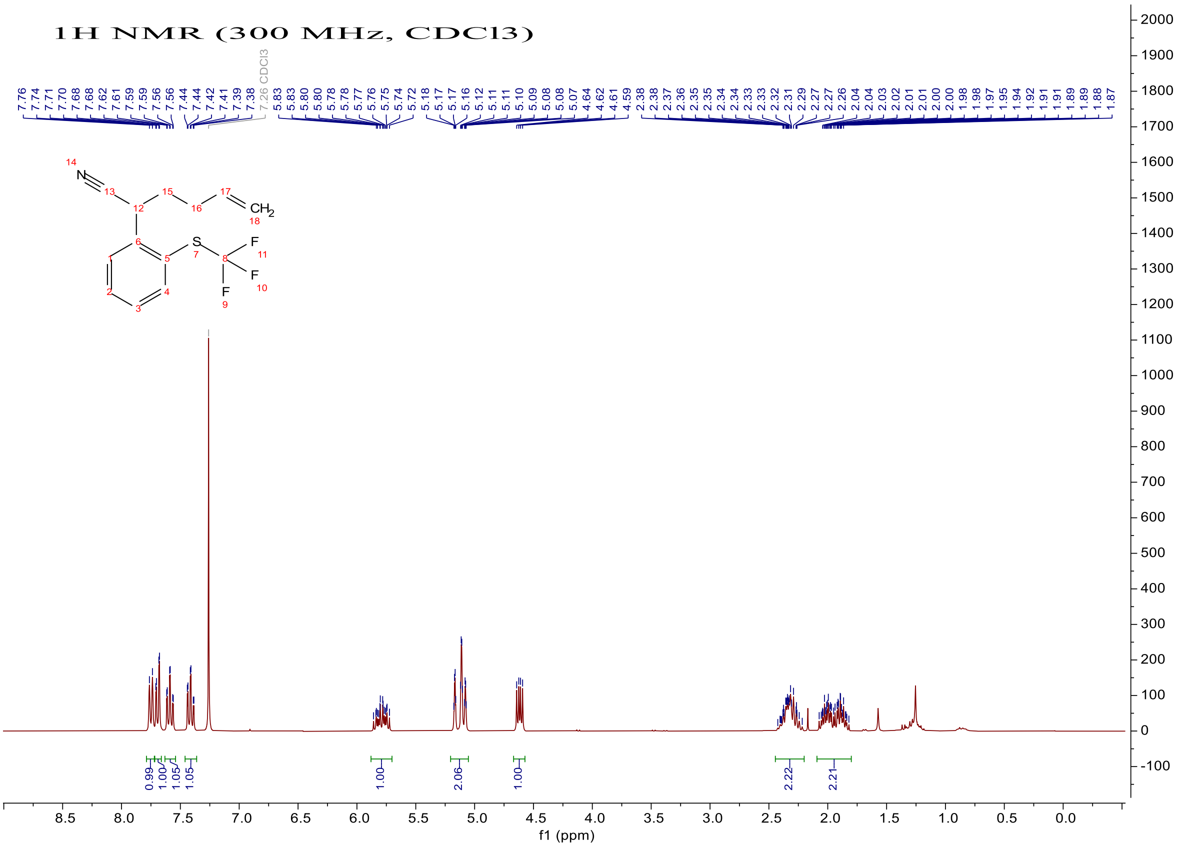
1H NMR (300 MHz, CDCl3)
7.76 7.74 7.71 7.70 7.68 7.68 7.62 7.61 7.59 7.59 7.56 7.56 7.44 7.44 7.42 7.41 7.39 7.38 7.26 CDCl3 5.83 5.83 5.80 5.80 5.78 5.78 5.77 5.76 5.75 5.74 5.72 5.18 5.17 5.17 5.16 5.12 5.11 5.11 5.10 5.09 5.08 5.08 5.07 4.64 4.62 4.61 4.59 2.38 2.38 2.37 2.36 2.35 2.35 2.34 2.34 2.33 2.33 2.32 2.31 2.29 2.27 2.27 2.26 2.04 2.04 2.03 2.02 2.01 2.01 2.00 2.00 1.98 1.98 1.97 1.95 1.94 1.92 1.91 1.91 1.89 1.88 1.87
0.99 1.00 1.05 1.05 1.00 2.06 1.00 2.22 2.21
f1 (ppm)

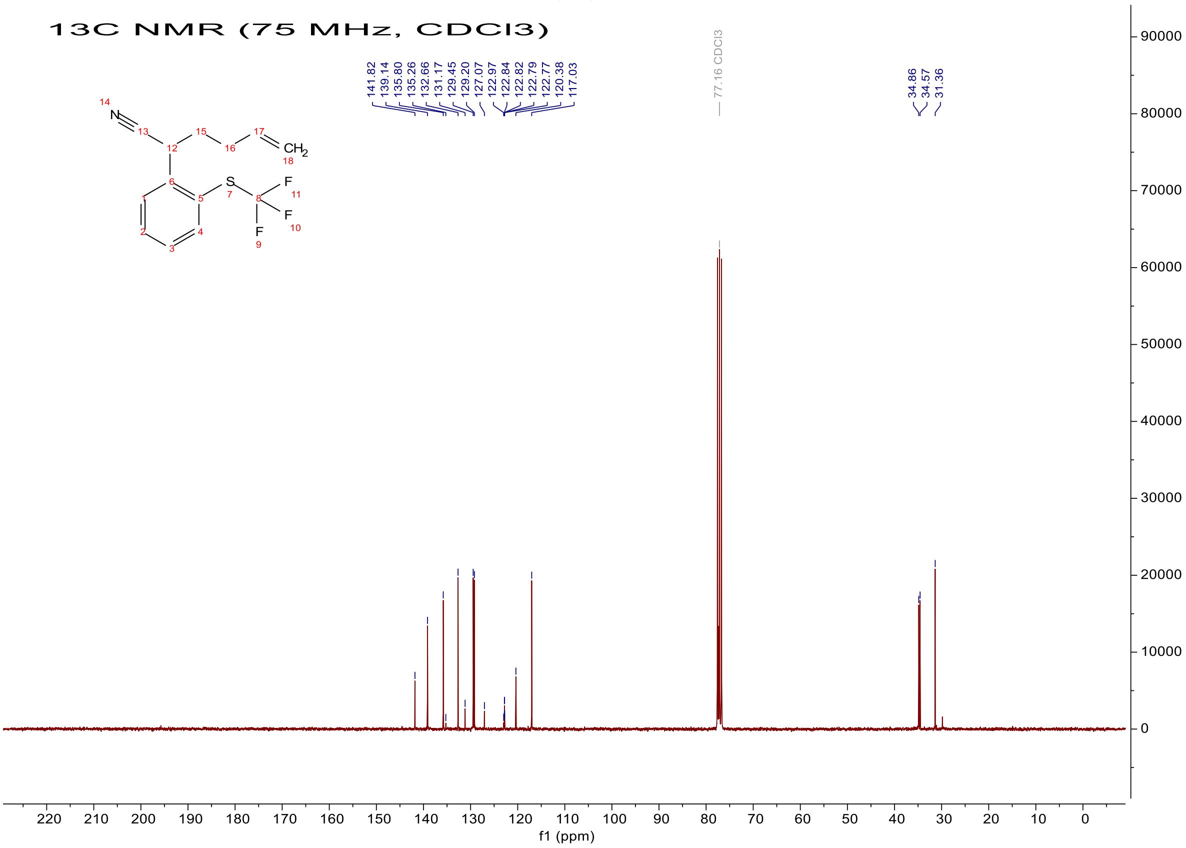
13C NMR (75 MHz, CDCl3)
141.82 139.14 135.80 135.26 132.66 131.17 129.45 129.20 127.07 122.97 122.84 122.82 122.79 122.77 120.38 117.03
77.16 CDCl3
34.86 34.57 31.36
f1 (ppm)

19F NMR (188 MHz, CDCl3)

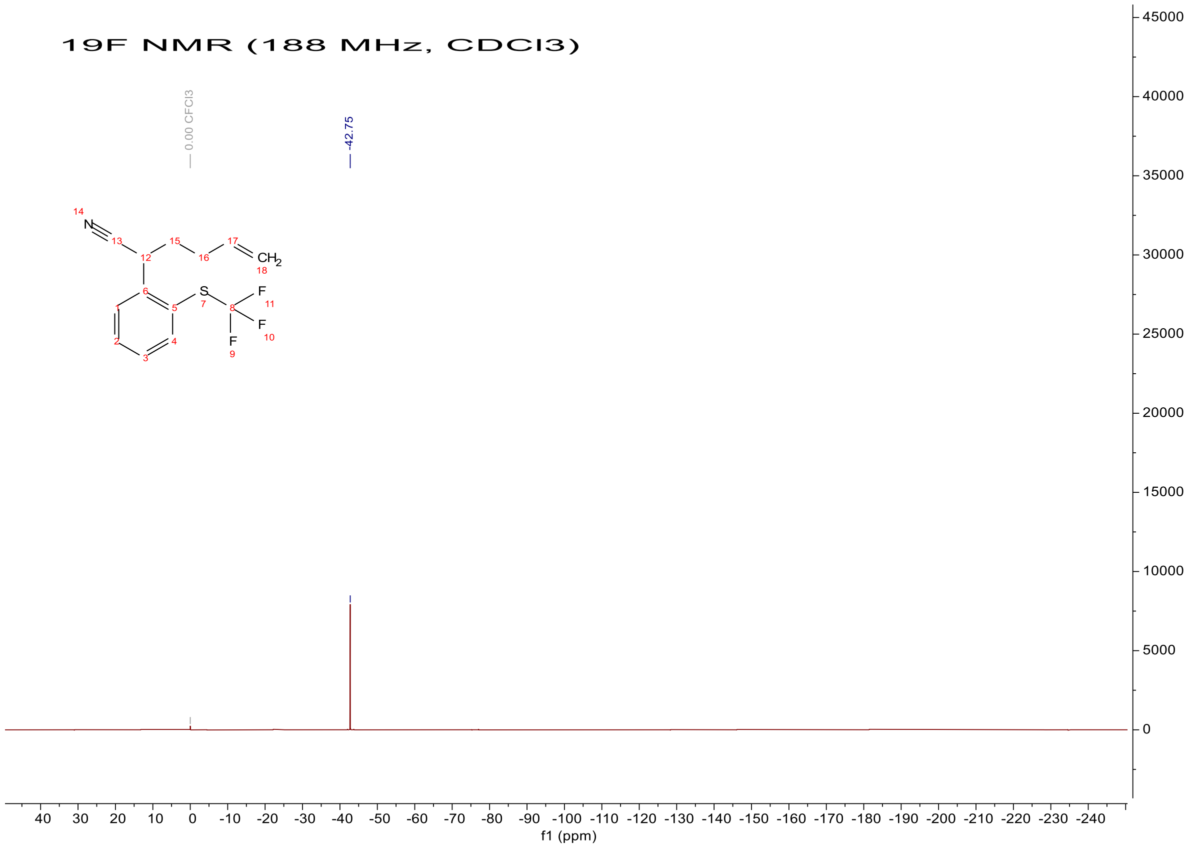

4-Chloro-2-(2-((trifluoromethyl)thio)phenyl)butanenitrile (3g)

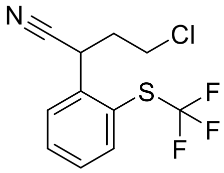

$C_{11}H_9ClF_3NS$
279.71

Reaction carried out following the general procedure 2 using ((trifluoromethyl)sulfinyl)benzene (97 mg, 0.5 mmol) with chlorobutyronitrile. NMR ^{19}F yield = 95%. Purified by flash chromatography (1st elution: gradient: petroleum ether/diethyl ether 100:0 to 80:20; 2nd elution: gradient: *n*-pentane/EtOAc 100:0 to 88:12), affording the product as an orange oil (131 mg, 0.47 mmol). Yield = 94%.

* **HRMS ASAP+TOF**: Calculated for $C_{11}H_{10}ClF_3NS[M+H]^+$: 280.0175; Found $[M+H]^+$: 280.0183

* **^{1}H NMR (300 MHz, $CDCl_3$) δ (ppm):** 7.78 (d, *J* = 8 Hz, 1H), 7.68 (dd, *J* = 8, 2 Hz, 1H), 7.60 (td, *J* = 8, 1 Hz, 1H), 7.44 (td, *J* = 8, 2 Hz, 1H), 4.94 (dd, *J* = 10, 6 Hz, 1H), 3.81 – 3.60 (m, 2H), 2.46 – 2.18 (m, 2H).

* **^{13}C NMR (75 MHz, $CDCl_3$) δ (ppm):** 140.5, 139.3, 132.8, 129.9, 129.2, 129.1 (q, *J* = 309 Hz), 123.2 (q, *J* = 2 Hz), 119.5, 41.2, 37.8, 32.7

* **^{19}F NMR (282 MHz, $CDCl_3$) δ (ppm):** -42.86 (s)

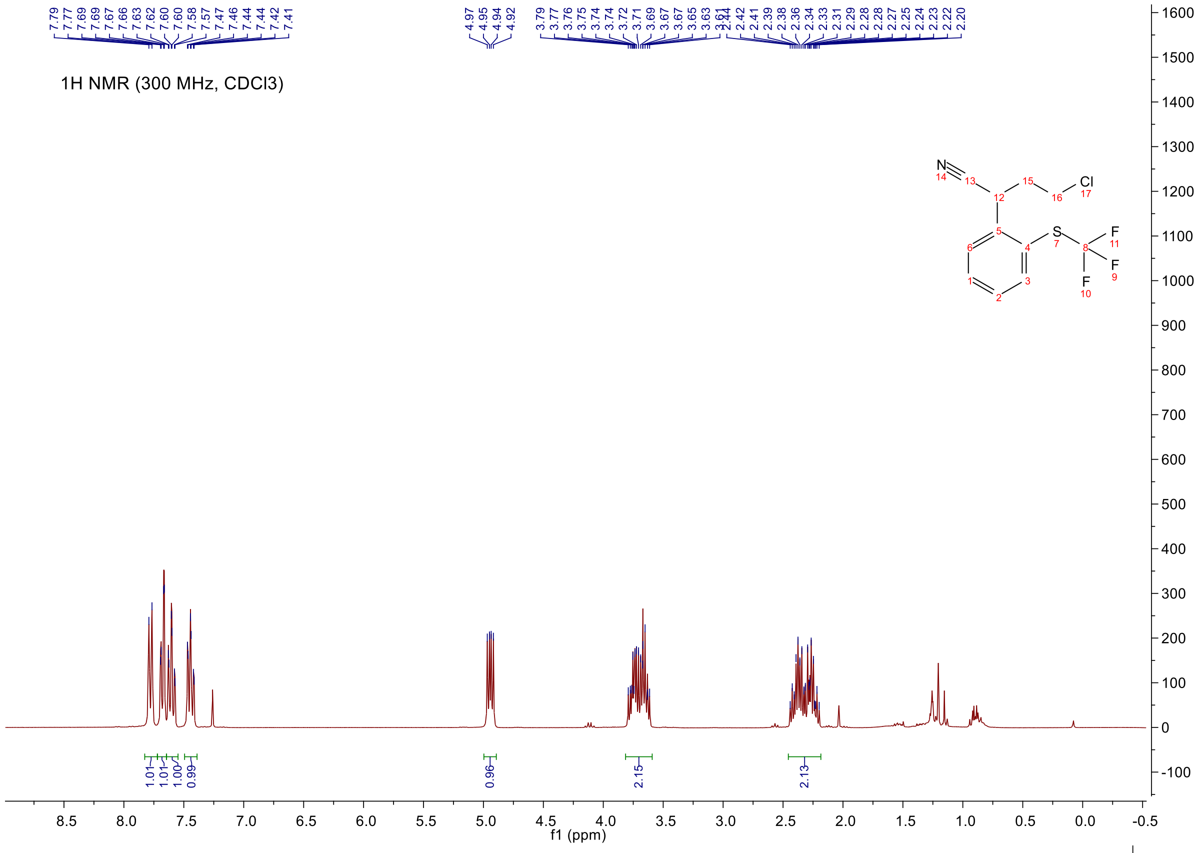

1H NMR (300 MHz, CDCl3)
7.79 7.77 7.69 7.69 7.67 7.66 7.63 7.62 7.60 7.60 7.58 7.57 7.47 7.46 7.44 7.44 7.42 7.41
4.97 4.95 4.94 4.92
3.79 3.77 3.76 3.75 3.74 3.74 3.72 3.71 3.69 3.67 3.67 3.65 3.63 3.61
2.44 2.42 2.41 2.39 2.38 2.36 2.34 2.33 2.31 2.29 2.28 2.28 2.27 2.25 2.24 2.23 2.22 2.20
1.01 1.01 1.00 0.99 0.96 2.15 2.13
f1 (ppm)

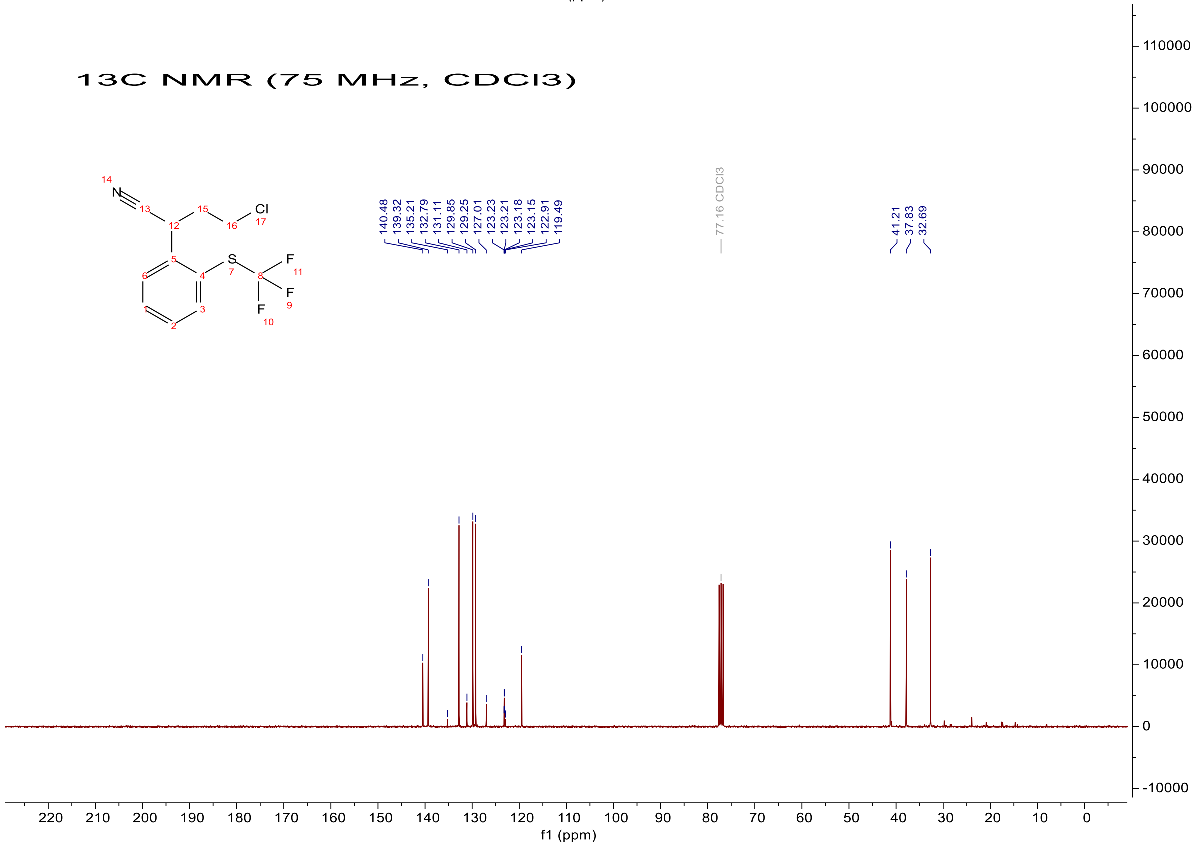

13C NMR (75 MHz, CDCl3)
140.48 139.32 135.21 132.79 131.11 129.85 129.25 127.01 123.23 123.21 123.18 123.15 122.91 119.49
77.16 CDCl3
41.21 37.83 32.69
f1 (ppm)

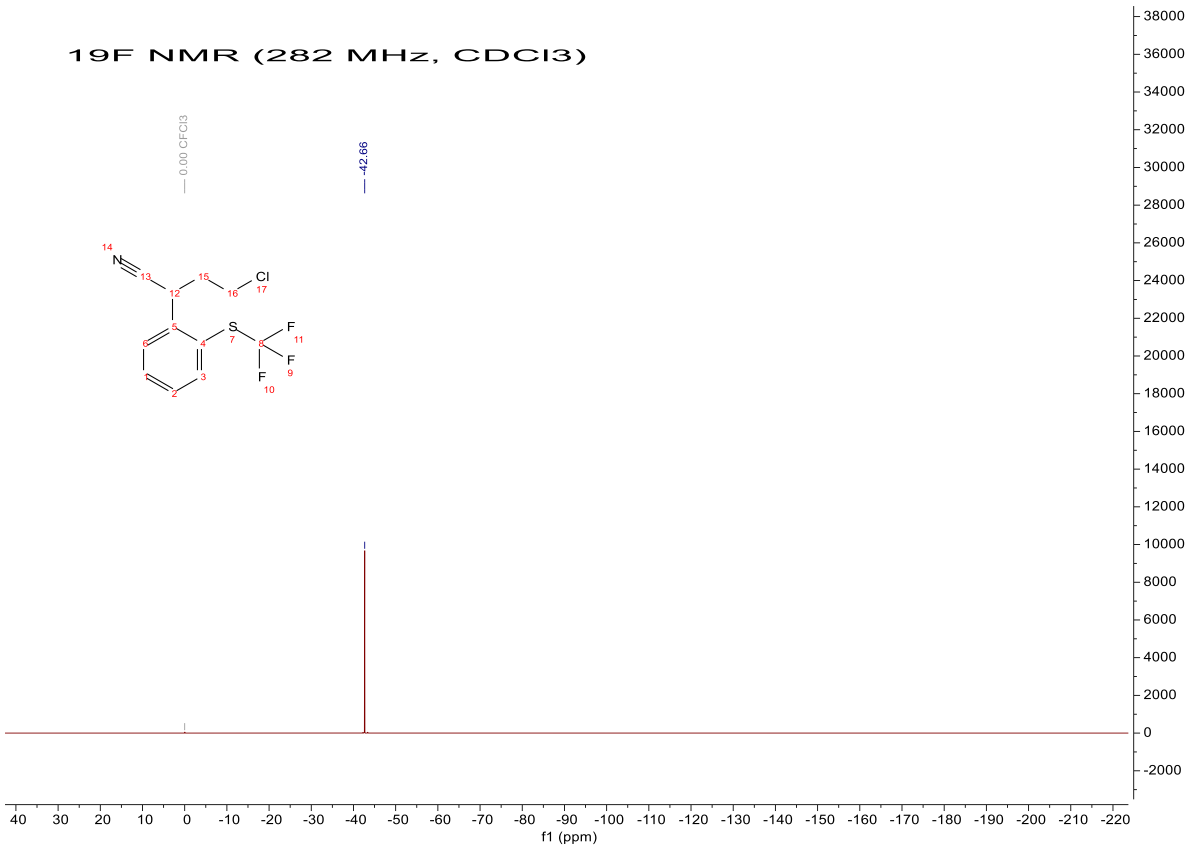

References

(1) Sokolenko, L. V.; Orlova, R. K.; Filatov, A. A.; Yagupolskii, Y. L.; Magnier, E.; Pégot, B.; Diter, P. *Molecules* **2019**, *24*, 1249. doi:10.3390/molecules24071249

(2) Wakselman, C.; Tordeux, M.; Freslon, C.; Saint-Jalmes, L. *Synlett* **2001**, *2001*, 0550–0552. doi:10.1055/s-2001-12316

(3) Ma, J.; Liu, Q.; Lu, G.; Yi, W. *Journal of Fluorine Chemistry* **2017**, *193*, 113–117. doi:10.1016/j.jfluchem.2016.11.010

(4) Air- and Light-Stable S-(Difluoromethyl)sulfonium Salts: C-Selective Electrophilic Difluoromethylation of β-Ketoesters and Malonates | Organic Letters https://pubs.acs.org/doi/10.1021/acs.orglett.8b03067 (accessed Apr 26, 2024)

(5) New Solid-Phase Bound Electrophilic Difluoromethylating Reagent | ACS Combinatorial Science https://pubs.acs.org/doi/10.1021/cc700148z (accessed Apr 26, 2024)

(6) de Zordo-Banliat, A.; Grollier, K.; Vanthuyne, N.; Floquet, S.; Billard, T.; Dagousset, G.; Pégot, B.; Magnier, E. *Angew. Chem. Int. Ed.* **2023**, *62*, e202300951. doi:10.1002/anie.202300951

(7) Macé, Y.; Urban, C.; Pradet, C.; Marrot, J.; Blazejewski, J.-C.; Magnier, E. *Eur. J. Org. Chem.* **2009**, *2009*, 3150–3153. doi:10.1002/ejoc.200900410
